# A Facile Synthesis of 2-Oxazolines via Dehydrative Cyclization Promoted by Triflic Acid

**DOI:** 10.3390/molecules27249042

**Published:** 2022-12-19

**Authors:** Tao Yang, Chengjie Huang, Jingyang Jia, Fan Wu, Feng Ni

**Affiliations:** 1 Institute of Drug Discovery Technology, Ningbo University, Ningbo 315211, China; 2 Qian Xuesen Collaborative Research Center of Astrochemistry and Space Life Sciences, Ningbo University, Ningbo 315211, China

**Keywords:** 2-oxazolines, dehydrative cyclization, green synthesis

## Abstract

2-oxazolines are common moieties in numerous natural products, pharmaceuticals, and functional copolymers. Current methods for synthesizing 2-oxazolines mainly rely on stoichiometric dehydration agents or catalytic dehydration promoted by specific catalysts. These conditions either generate stoichiometric amounts of waste or require forcing azeotropic reflux conditions. As such, a practical and robust method that promotes dehydrative cyclization while generating no byproducts would be attractive to oxazoline production. Herein, we report a triflic acid (TfOH)-promoted dehydrative cyclization of *N*-(2-hydroxyethyl)amides for synthesizing 2-oxazolines. This reaction tolerates various functional groups and generates water as the only byproduct. This method affords oxazoline with inversion of α-hydroxyl stereochemistry, suggesting that alcohol is activated as a leaving group under these conditions. Furthermore, the one-pot synthesis protocol of 2-oxazolines directly from carboxylic acids and amino alcohols is also provided.

## 1. Introduction

**Figure 1:**
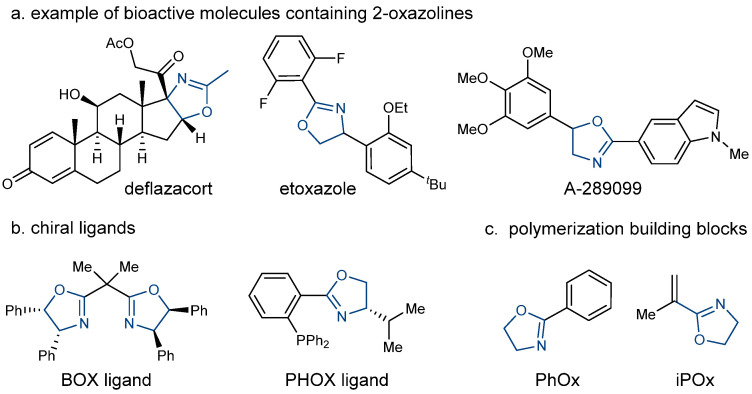
Functional molecules containing 2-oxazoline moiety.

2-oxazoline is a privileged structural motif in numerous bioactive molecules and pharmaceuticals [1,2,3,4,5,6,7] (Figure 1a) as well as functional copolymers [8,9,10,11,12,13,14,15] (Figure 1c). Various natural products and synthetic molecules that contain this structural unit possess biological activities, such as antibiotics [16,17], antineoplastics [18,19,20], anti-fungals [21], and anti-inflammatories [22], among others. Furthermore, 2-oxazolines have a wide range of synthetic applications, including protective groups for carboxylic acid and aldehyde, directing groups in C-H functionalization, and valuable chiral Box and Pybox ligands [23,24,25,26] (Figure 1b). These important applications have fueled the development of various approaches to the efficient construction of 2-oxazolines over the last few decades. The typical approaches involve coupling amino alcohols with carboxylic acid derivatives [27,28,29,30,31], nitriles [32,33,34], and aldehydes [35,36,37] in the presence of activation reagents, catalysts, or oxidants. Recently, the functionalization of alkenes with amides provided a valuable alternative approach to the 2-oxazoline synthesis [38,39,40,41,42]. Although these advances expanded the chemist’s toolbox for 2-oxazoline synthesis, developing practical and cost-effective new methods for constructing 2-oxazolines would complement current methods.

**Figure 2:**
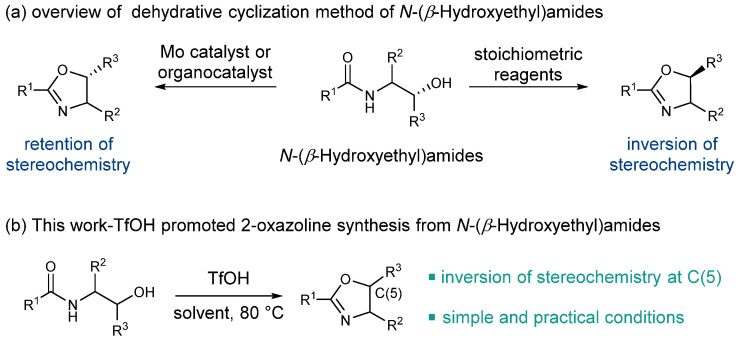
Dehydrative cyclization of *N*-(*β*-hydroxyethyl)amides.

Despite significant advances in 2-oxazoline synthesis, dehydrative cyclization of *N*-(*β*-hydroxyethyl)amides remains the most widely used method for producing 2-oxazolines. Numerous stoichiometric reagents, including DAST, XtalFluor-E, PPE, Ph_3_P/DEAD, and Burgess reagent, have proven to be efficient at forging the oxazoline moiety [43,44,45,46,47,48,49,50,51,52,53] (Figure 2a). These conditions generally require either harsh conditions or corrosive reagents, which may cause additional operating costs and stoichiometric byproduct generation. To address this issue, several groups have developed catalytic dehydrative approaches [54,55,56,57]. The Ishihara group reported a molybdenum complex-catalyzed dehydrative cyclization of *N*-(2-hydroxyethyl)amides [54,55]. Saito and co-workers demonstrated a phosphorus-based organocatalytic dehydrative cyclization approach [57] (Figure 2a). In addition, one example of cyclization catalyzed by sulfuric acid was also reported but under harsh high-temperature conditions [58]. While these methods avoid using stoichiometric dehydration agents and thus have higher atom economy, the requirement for specific catalysts and forcing azeotropic reflux conditions might limit their industrial application. As a result, a practical and robust method that promotes dehydrative cyclization while generating no byproducts would be attractive to oxazoline production. Herein, we report our effort in the TfOH-promoted synthesis of 2-oxazolines by dehydrative cyclization of *N*-(2-hydroxyethyl)amides (Figure 2b).

## 2. Results

### 2.1. Optimization of the Reaction Conditions

We began our reaction optimization by examining the cyclization reaction of *β*-hydroxyamide **1** in the presence of several organic acids in 1,2-dichloroethane (DCE) (Table 1, entries 1–3). It was found that TfOH in DCE at 80 °C effectively promoted the formation of the desired 2-oxazoline. The acidity of the acid seemed to be important, as weaker acids such as MsOH and TFA only afforded product in low yields (Table 1, entries 1–2). Stoichiometry optimization on acid (Table 1, entries 4–8) revealed that a 1.5 equivalent of TfOH was optimal (Table 1, entry 7). Several other solvents (Table 1, entries 9–11) gave similar results albeit in a slightly lower yield than DCE, suggesting no significant solvent effect for this transformation. In addition, running the reaction at lower temperatures afforded product **2** in lower yields (Table 1, entries 12–14).

### 2.2. Substrate Scope Studies

**Figure 3:**
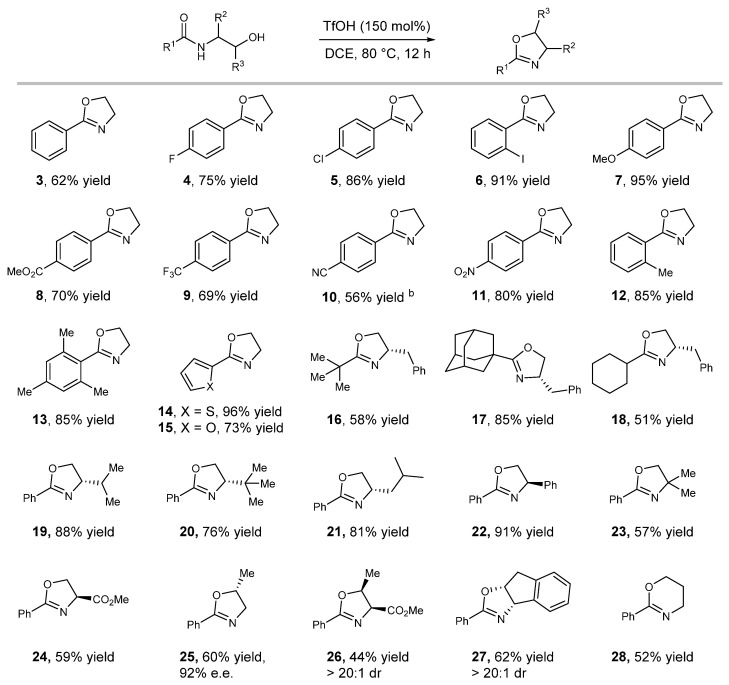
Scope of dehydrative cyclization reaction. Standard reaction conditions, 12 h. The yields shown here are isolated yield. ^b^ Running reaction at 60 °C instead of 80 °C.

With the optimized reaction conditions in hand, we then investigated the generality of this protocol. We initially tested a range of substrates derived from monosubstituted benzoic acid and ethanolamine. Functional groups, such as halides, ether, ester, CF_3_, and nitro, were well tolerated in standard reaction conditions and afforded the desired products in good to excellent yields (Figure 3, products **3**–**11**). Although generally unstable under acidic conditions in the presence of water, the substrate with the cyano group also gave product albeit in a lower yield. It appears that the steric hindrance had a minimal impact on the reactivity as evident by the similar yield observed in the reaction of the sterically hindered substrates (Figure 3, products **12** and **13**). *N-*(2-hydroxyethyl)amides derived from 2-thiophenecarboxylic acid and 2-furoic acid were also viable substrates, delivering the desired products **14** and **15** in 96% and 73% yield, respectively. *N-*(2-hydroxyethyl)amides derived from secondary and tertiary aliphatic acids proceeded smoothly under standard conditions affording the desired 2-oxazolines with moderate to good yields (Figure 3, products **16**–**18**). We then turned our attention to exploring the substrates derived from β-substituted 1,2-amino alcohols. The substrates derived from L-valinol, L-tert-Leucinol, L-Leucinol, D-Phenylglycinol, 2-amino-2-methyl-1-propanol, and D-serine methyl ester were all viable substrates and delivered the desired products in good to excellent yields (Figure 3, products **19**–**24**). Moreover, the substrates derived from (S)-(+)-1-Amino-2-propanol, L-Threonine methyl ester and (1S, 2R)-(−)-cis-1-amino-2-indanol that bear α-substitution, and α, β-disubstitution were also well tolerated in this protocol (Figure 3, products **25**–**27**). Notably, products **26** and **27** were isolated as a single diastereomer, and no other diastereomers were detected from crude NMR. Mechanistic studies suggested that products **25** and **26** were formed with an inversion of the stereochemistry at carbon β. Depending on the starting material, a product with a rigid backbone such as **27** can be generated with either inversion or retention of the stereochemistry at position β. In addition, 1,3-amino alcohol derivative afforded 5,6-dihydro-4H-1,3-oxazine in moderate yields (Figure 3, product **28**).

**Figure 4:**
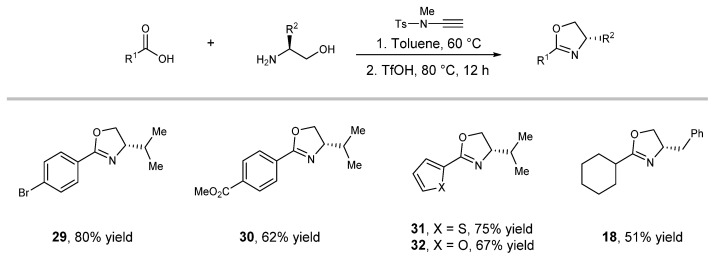
Scope of one-pot synthesis of 2-oxazolines. See the Supplementary Materials for details.

Given the robustness of this practical protocol, we envisioned the possibility of a one-pot synthesis of 2-oxazolines directly from the carboxylic acid and 1,2-amino alcohols. To construct a TfOH-friendly system, we tested the base-free ynamide invented by Zhao [59] as a coupling reagent. A variety of oxazolines were successfully synthesized in a one-pot fashion via in situ coupling of carboxylic acids with amino alcohols followed by cyclization under standard conditions. (Figure 4, products **29**–**32**, **18**).

### 2.3. Control Experiments and Mechanistic Studies

**Figure 5:**
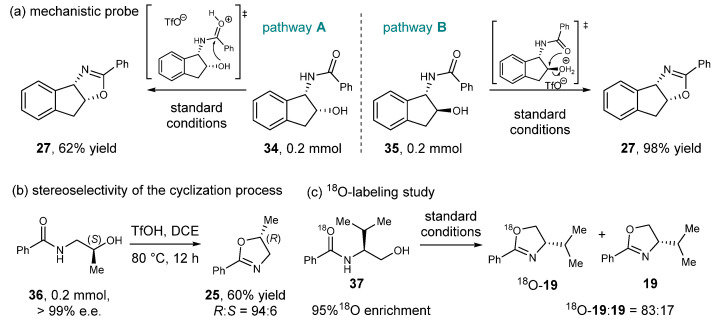
Mechanistic studies.

According to previous reports, this reaction has two possible pathways that result in products with opposite stereochemical outcomes. One pathway involves acid activation of the amide carbonyl group followed by nucleophilic attack of the hydroxyl group resulting in 2-oxazoline with retention of stereochemistry (Figure 5a, pathway A). Alcohol activation followed by intramolecular S_N_2-like substitution, on the other hand, would produce cyclized products with reversed *a*-hydroxyl stereochemistry (Figure 5a, pathway B). We then conducted several control experiments to study the reaction mechanism. Our studies started from treating sterically rigid *cis*-*β*-hydroxyl amide **34** and *trans*-*β*-hydroxyl amide **35** with standard conditions to probe the possible reaction pathway (Figure 5a). Surprisingly, the formation of product **27** was observed in both cases, suggesting that both pathways are operatable under standard conditions. While the higher yield obtained from **35** suggested that pathway B might be more favored, more information is required to gain a better understanding of the mechanism. We then subjected enantiopure *β*-hydroxyl amide **36** to the reaction conditions and analyzed the stereoselectivity using chiral HPLC (Figure 5b). 2-oxazoline **25** was obtained with stereochemical inversion as the major isomer (94:6 e.r.), which indicates that the pathway involving alcohol activation is more favored. We think that the erosion of optical purities observed in product **25** might result from a hybrid reaction pathway. To validate this hypothesis, we conducted the ^18^O labeling experiment. *N*-(2-hydroxyethyl)amides **37** with 95% ^18^O enrichment was smoothly converted to product, and the ratio of **^18^O**-**19** and **19** was 83:17 (Figure 5c). These data are consistent with the hypothesis of a hybrid mechanism, in which activation of the hydroxyl group is the dominant pathway under our reaction condition.

## 3. Conclusions

In conclusion, a practical and effective strategy for synthesizing 2-oxazolines via dehydrative cyclization of *N***-**(2-hydroxyethyl)amides has been developed. This efficient cyclization process was promoted by TfOH and had good functional group tolerance. Stereoselectivity and ^18^O labeling data suggested that the reaction might proceed through a hybrid mechanism, in which activation of the hydroxyl group is the dominant pathway. Notably, this robust reaction condition can be adapted to a one-pot reaction by directly utilizing readily available carboxylic acid and amino alcohols.

## Figures and Tables

**Table 1:** Optimization of the reaction conditions ^a^.

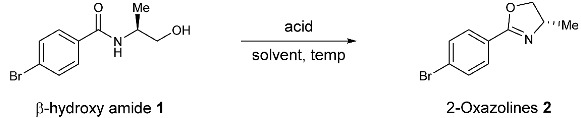				
Entry	Acid (Equiv.)	Solvent	Temperature	Yield ^b^
1	MsOH (1.0)	DCE	80 °C	16
2	TFA (1.0)	DCE	80 °C	9
3	TfOH (1.0)	DCE	80 °C	89
4	TfOH (0.2)	DCE	80 °C	14
5	TfOH (0.5)	DCE	80 °C	29
6	TfOH (1.2)	DCE	80 °C	94
7	TfOH (1.5)	DCE	80 °C	96 (88) ^c^
8	TfOH (2.0)	DCE	80 °C	86
9	TfOH (1.5)	Toluene	80 °C	92
10	TfOH (1.5)	PhCF_3_	80 °C	86
11	TfOH (1.5)	CH_3_CN	80 °C	95
12	TfOH (1.5)	DCE	70 °C	91
13	TfOH (1.5)	DCE	60 °C	85
14	TfOH (1.5)	DCE	25 °C	<5

^a^ Reaction conditions: *N*-(2-hydroxyethyl)amide 1 (0.2 mmol), acid (0.2–2.0 equiv) and solvent (1 mL) at 25–80 °C, t = 12h. ^b^ NMR yield using 1,3-benzodioxole as the internal standard; the NMR yield was calculated based on the ratio of CH_2_ signal (5.8 ppm) of 1,3-benzodioxole and CH signal of product **2** (4.5 ppm). ^c^ Isolated yield.

## Data Availability

The data presented in this study are available upon request from the authors.

## Supplementary Materials

The following supporting information can be downloaded at: https://www.mdpi.com/article/10.3390/molecules27249042/s1, Table S1: Reaction optimization; Figure S1: Control experiments on stereochemical outcome of C(4) position; Figure S2: The HPLC analysis of the (S)-oxazoline **19** and (R)-oxazoline **19**; Figure S3: Cis- and trans- 1-amino-2-indanol derived mechanistic probe; Figure S4: Stereochemical outcome of this protocol; Figure S5: HPLC analysis of oxazoline product **25**; Figure S6: 18O-labeling study of product **19**; Figure S7: HRMS data of the 18O-labeled N-(2-hydroxyethyl)amides and oxazoline **19**.

## Sample Availability

Samples of the compounds are not available from the authors.
