# Supplementary material for: A Facile Synthesis of 2-Oxazolines via Dehydrative Cyclization Promoted by Triflic Acid
Source: Molecules. 2022 Dec 19;27(24):9042. doi: 10.3390/molecules27249042 (PMC9781752; doi:10.3390/molecules27249042)

Supplementary Materials

A Facial Synthesis of 2-Oxazolines via Dehydrative Cyclization Promoted by Triflic Acid

Tao Yang [a,b], Chengjie Huang [a,b], Jingyang Jia [a,b], Fan Wu[*a,b], Feng Ni[*a,b]

[a] Institute of Drug Discovery Technology, Ningbo University, Ningbo, Zhejiang, 315211, P. R. China.

[b] Qian Xuesen Collaborative Research Center of Astrochemistry and Space Life Sciences, Ningbo University, Ningbo, Zhejiang, 315211, P. R. China.

E-mail: wufan@nbu.edu.cn (F. W.); nifeng@nbu.edu.cn (F. N.)

Content

S1. General Information ··1

S2. Optimization Experiments··2

S3. Control experiments and mechanistic studies ··· 3−5

S4. Dehydrative Cyclization Reaction and Spectral Characterization of the Products ·········6−19

S5. NMR spectrums ··20−52

S1. General Information

Commercial reagents were purchased from Adamas, Aldrich, Bide, Energy Chemical, and J&K chemical, and were used as received. Chromatographic purification of products was accomplished using flash chromatography on 200-300 mesh silica gel. ^{1}H, ^{13}C and ^{19}F NMR spectra were recorded on a Bruker instrument (500 MHz for ^{1}H NMR, 126 MHz for ^{13}C and 470 MHz for ^{19}F) and are internally referenced to chemical shift of residual solvent (for $CDCl_3$, 7.26 and 77.16 ppm, respectively). Data for ^{1}H NMR are reported as follows: chemicals shift (ppm), multiplicity (s = singlet, d = doublet, t = triplet, q = quartet, m = multiplet, br = broad), integration, coupling constant (Hz). ^{13}C spectra were recorded as chemical shifts in ppm and multiplicity where appropriate. ^{19}F NMR data were with complete proton decoupling and the chemical shifts were reported in ppm. High resolution mass spectroscopy (HR-MS) was performed on Thermo Q Exactive Plus (FTMS ESI) mass spectrometer and acetonitrile was used to dissolve the sample.

S2. Optimization Experiments

To an oven-dried 8 mL vial equipped with a magnetic stir bar was added (S)-4-bromo-N-(1-hydroxypropan-2-yl)benzamide (52 mg, 0.2 mmol) and solvent (1 mL), followed by the addition of acid (0.04-0.3 mmol). The reaction mixture was then stirred at the corresponding temperature for 12 hours. After cooling to room temperature, the reaction mixture was diluted with EtOAc (5 mL) and washed with 1M Na_2CO_3 solution (2 mL). The organic layer was separated, and the aqueous layer was extracted with EtOAc (2 mL×2). The combined organic layers were dried over Na_2SO_4 and concentrated under reduced pressure to afford the crude product. Then 1,3-benzodioxole (12.2 mg, 0.1 mmol) was added as an internal standard and subjected to ^{1}H NMR. The NMR yield was calculated based on the integration ratio of products and internal standards.

Table S1 Reaction optimization [a]

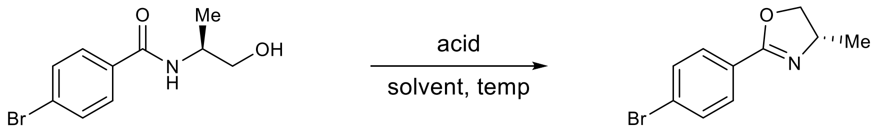

β-hydroxy amide **1** 2-Oxazolines **2**

Entry	acid (equiv.)	solvent	Temperature	yield (%)[b]
1	MsOH (1.0)	DCE	80°C	16
2	TFA (1.0)	DCE	80°C	9
3	TfOH (1.0)	DCE	80°C	89
4	TfOH (0.2)	DCE	80°C	14
5	TfOH (0.5)	DCE	80°C	29
6	TfOH (1.2)	DCE	80°C	94
7	TfOH (1.5)	DCE	80°C	96 (88)[c]
8	TfOH (2.0)	DCE	80°C	86
9	TfOH (1.5)	Toluene	80°C	92
10	TfOH (1.5)	$PhCF_3$	80°C	86
11	TfOH (1.5)	CH_3CN	80°C	95
12	TfOH (1.5)	DCE	70°C	91
13	TfOH (1.5)	DCE	60°C	85
14	TfOH (1.5)	DCE	25°C	<5

[a] Reaction conditions: *N*-(2-hydroxyethyl)amide 1 (0.2 mmol), acid (0.2–2.0 equiv) and solvent (1 mL) at 25–80 °C, t = 12h. [b] NMR yield using 1,3-benzodioxole as the internal standard; the NMR yield was calculated based on the ratio of CH_2 signal (5.8 ppm) of 1,3-benzodioxole and CH signal of product **2** (4.5 ppm). [c] Isolated yield.

S3. Control experiments and mechanistic studies

S3.1 Configuration of C(4) position

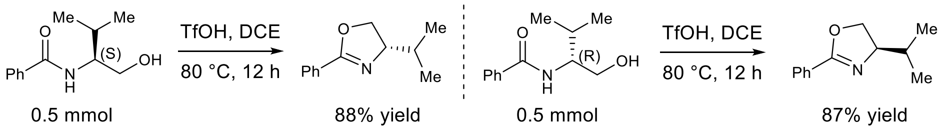

Figure S1 Control experiments on stereochemical outcome of C(4) position

Enantiopure (S)-N-(1-hydroxy-3-methylbutan-2-yl)benzamide or (R)-N-(1-hydroxy-3-methylbutan -2-yl)benzamide was subjected to standard reaction condition in a 0.5 mmol scale. Oxazoline product was purified by flash column chromatography (10% EtOAc/PE to 30% EtOAc/PE) to afford final product in 88% and 87% yield, respectively. HPLC analysis (OD-H, 2-propanol/n-hexane = 20/80, flow rate = 1.0 mL/min, λ = 258 nm) indicated that no erosion of optical purities on C(4) position.

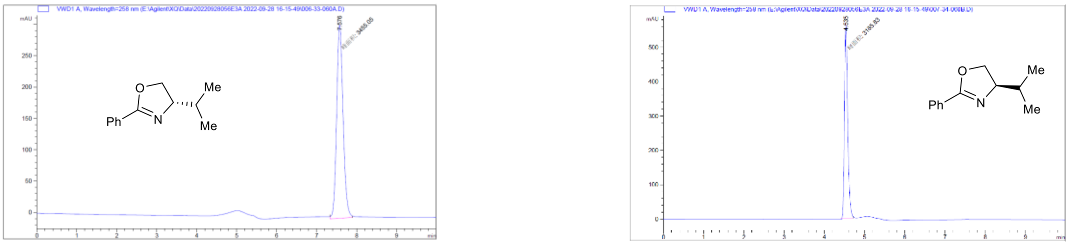

FigureS2 The HPLC analysis of the (S)-oxazoline **19** and (R)-oxazoline **19**

S3.2 Cis- and trans- 1-amino-2-indanol derived N-(2-hydroxyethyl)amides as mechanistic probe

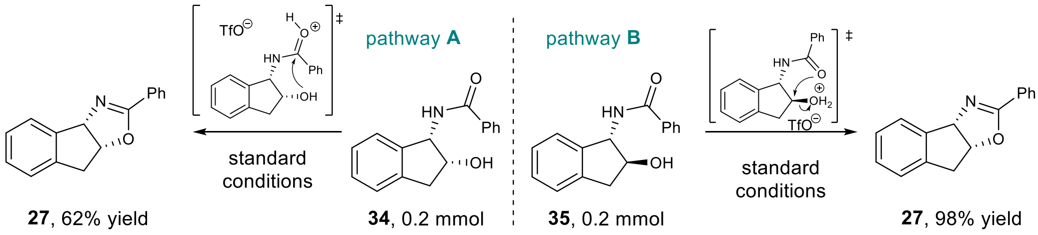

Figure S3 *Cis*- and *trans*- 1-amino-2-indanol derived mechanistic probe

N-(2-hydroxyethyl)amides **34** and **35** were synthesized from the amidation of (1S,2R)-(-)-cis-1-Amino-2-indanol and (1S,2S)-(+)-trans-1-amino-2-indanol, and treated with TfOH under standard reaction conditions. Product **27** was detected as the single diastereomer for both cases, albeit in different yield. Based on the observation that no optical erosion on C(4) position occur under standard conditions, this result suggest that both pathway A and pathway B are operatable in the reaction condition.

S3.3 Stereoselectivity of the cyclization process

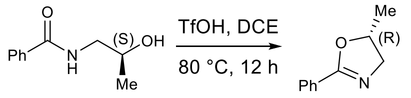

36, 0.2 mmol,
> 99% e.e.

25, 60% yield
R:S = 94:6

Figure S4 stereochemical outcome of this protocol

Enantiopure N-(2-hydroxyethyl)amide **36** was subjected to standard reaction condition in a 0.2 mmol scale. Oxazoline product was purified by flash column chromatography (20% EtOAc/PE to 30% EtOAc/PE) to afford final product in 60% yield. HPLC analysis (OD-H, 2-propanol/n-hexane = 5/95, flow rate = 1.0 mL/min, λ = 258 nm) indicated that the product is a mixture of enantiomers in 94:6 ratio.

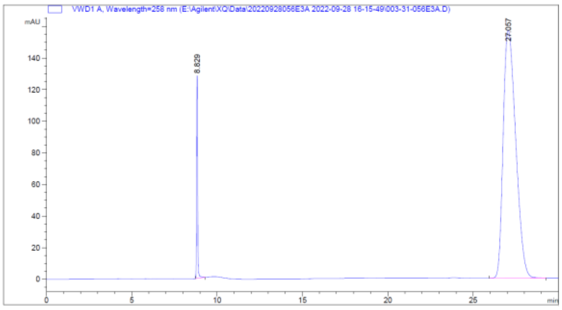

#	[min]		[min]	[mAU*s]	[mAU]	%
1	8.829	BB	0.0640	528.47803	128.38864	6.1965
2	27.057	BB	0.8093	8000.14941	156.80202	93.8035

Figure S5 HPLC analysis of oxazoline product **25**

S3.4 ^{18}O-labeling study

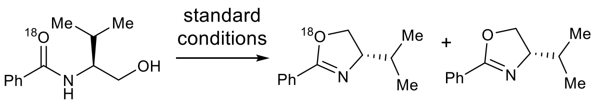

37 ^{18}O-**19** **19**

95% ^{18}O enrichment

^{18}O-**19**:**19** = 83:17

Figure S6 ^{18}O-labeling study of product **19**

^{18}O labeled N-(2-hydroxyethyl)amide **37** was synthesized following reported procedures[1], and subjected to standard reaction condition in a 0.2 mmol scale. Oxazoline product was purified by flash column chromatography (10% EtOAc/PE to 30% EtOAc/PE) to afford final product in 86% yield. HRMS analysis indicated that the product is the mixture of ^{18}O-**19** and **19** in about 83:17 ratio.

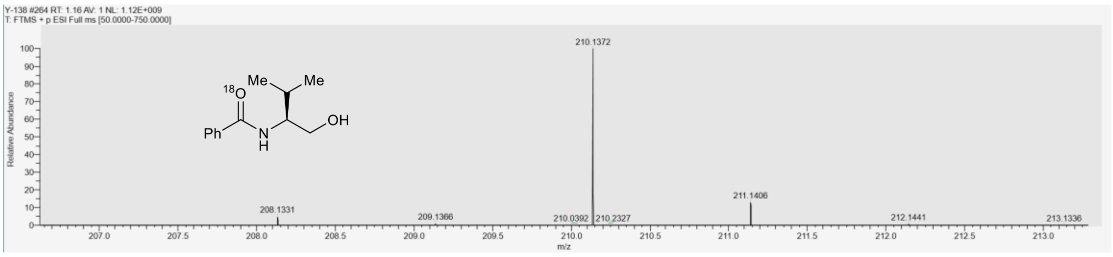

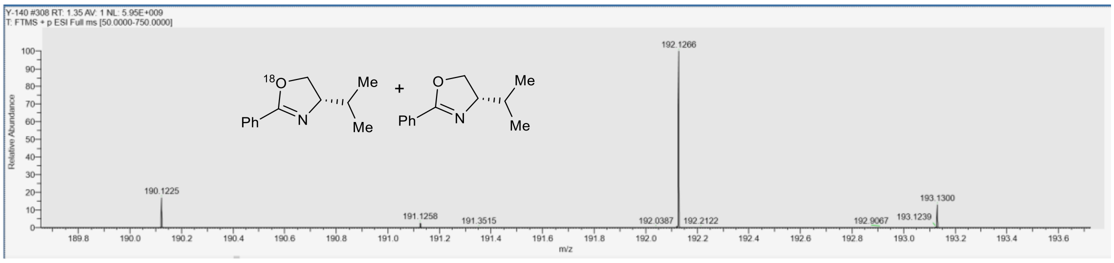

Figure S7 HRMS data of the ^{18}O-labeled *N*-(2-hydroxyethyl)amides and oxazoline **19**

S4. Dehydrative Cyclization of N-(2-hydroxyethyl)amides

S4.1 General Procedures

General Procedure A: dehydrative cyclization of N-(2-hydroxyethyl)amides

To an oven-dried 8 mL vial equipped with a magnetic stir bar was added N-(β-Hydroxyethyl)amides (0.2 mmol) and DCE (1 mL), followed by the addition of TfOH (27 µL, 0.3 mmol). The reaction mixture was then stirred at 80℃ for 12 hours. After cooling to room temperature, the reaction mixture was diluted with EtOAc (5 mL), washed with 1M Na_2CO_3 solution (2 mL). The organic layer was separated, and the aqueous layer was extracted with EtOAc (2 mL×2). The combined organic layers were dried over Na_2SO_4 and concentrated under reduced pressure to afford the crude product. The crude product was purified by column chromatography to afford pure product.

General Procedure B（one-pot）: one-pot synthesis of oxazoline from carboxylic acids and amino alcohols.

To an oven-dried 8 mL vial equipped with a magnetic stir bar was added carboxylic acid (0.2 mmol) and N-Ethynyl-N,4-dimethylbenzenesulfonamide (46 mg, 0.22 mmol), followed by the addition of Toluene (1 mL). The reaction mixture was stirred at 60℃ for 3 hours before the addition of corresponding amino alcohol (0.24 mmol). The resulting reaction mixture was stirred at this temperature for another 24 hours. After cooling to room temperature, TfOH (36 µL, 0.4 mmol) was added to the reaction mixture and stirred at 80℃ for 12 hours. The reaction mixture was cooled to room temperature and diluted with EtOAc (5 mL), washed with 1M Na_2CO_3 solution (2 mL). The organic layer was separated, and the aqueous layer was extracted with EtOAc (2 mL×2). The combined organic layers were dried over Na_2SO_4 and concentrated under reduced pressure to afford the crude product. The crude product was purified by column chromatography to afford pure product.

S4.2 Spectral Characterization of the Products

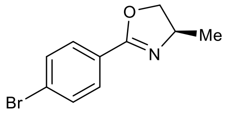

(R)-2-(4-bromophenyl)-4-methyl-4,5-dihydrooxazole (2): This compound was prepared according to the General Procedure A, using (R)-4-bromo-N-(1-hydroxypropan-2-yl)benzamide (52.0 mg, 0.2 mmol), trifluoromethanesulfonic acid (45.0 mg, 27μL, 0.3 mmol). After purification by column chromatography (SiO_2, 20% EtOAc in PE to 40% EtOAc in PE), the title compound was isolated as a white solid (42.1 mg, 88% yield). ^{1}H NMR (500 MHz, $CDCl_3$) δ 7.79 (d, *J* = 8.4 Hz, 2H), 7.53 (d, *J* = 8.4 Hz, 2H), 4.53 (dd, *J* = 9.4, 8.0 Hz, 1H), 4.43 – 4.32 (m, 1H), 3.96 (t, *J* = 8.0 Hz, 1H), 1.37 (d, *J* = 6.6 Hz, 3H).

^{13}C NMR (126 MHz, $CDCl_3$) δ 162.9, 131.7, 129.9, 126.9, 126.0, 74.4, 62.2, 21.5.

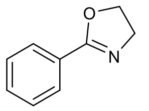

2-phenyl-4,5-dihydrooxazole (3): This compound was prepared according to the General Procedure A, using N-(2-hydroxyethyl)benzamide (33.0 mg, 0.2 mmol), trifluoromethanesulfonic acid (45.0 mg, 27μL, 0.3 mmol). After purification by column chromatography (SiO_2, 20% EtOAc in PE to 50% EtOAc in PE), the title compound was isolated as a white solid (18.3 mg, 62% yield).

^{1}H NMR (500 MHz, $CDCl_3$) δ 8.01 – 7.89 (m, 2H), 7.49 – 7.44 (m, 1H), 7.43 – 7.36 (m, 2H), 4.42 (t, *J* = 9.5 Hz, 2H), 4.05 (t, *J* = 9.5 Hz, 2H).

^{13}C NMR (126 MHz, $CDCl_3$) δ 164.8, 131.4, 128.5, 128.3, 127.8, 67.7, 55.0.

HRMS (ESI) *m/z* calcd for $C_9H_{10}NO^+$ [$(M+H)^+$] 148.0757, found 148.0755.

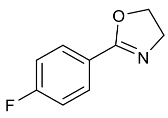

2-(4-fluorophenyl)-4,5-dihydrooxazole (4): This compound was prepared according to the General Procedure A, using 4-fluoro-N-(2-hydroxyethyl)benzamide (36.6 mg, 0.2 mmol), trifluoromethanesulfonic acid (45.0 mg, 27μL, 0.3 mmol). After purification by column chromatography (SiO_2, 20% EtOAc in PE to 50% EtOAc in PE), the title compound was isolated as a yellow solid (24.9 mg, 75% yield).

^{1}H NMR (500 MHz, $CDCl_3$) δ 8.02 – 7.87 (m, 2H), 7.16 – 7.01 (m, 2H), 4.42 (t, *J* = 9.5 Hz, 2H), 4.04 (t, *J* = 9.5 Hz, 2H).

^{13}C NMR (126 MHz, $CDCl_3$) δ 165.8, 163.9, 163.8, 130.6, 130.5, 124.1, 124.1, 115.7, 115.5, 67.9, 55.0.

^{19}F NMR (471 MHz, $CDCl_3$) δ -108.37.

HRMS (ESI) *m/z* calcd for $C_9H_9FNO^+$ $[(M+H)^+]$ 166.0663, found 166.0660.

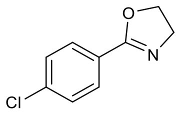

2-(4-chlorophenyl)-4,5-dihydrooxazole (5): This compound was prepared according to the General Procedure A, using 4-chloro-N-(2-hydroxyethyl)benzamide (39.9 mg, 0.2 mmol), trifluoromethanesulfonic acid (45.0 mg, 27μL, 0.3 mmol). After purification by column chromatography (SiO_2, 20% EtOAc in PE to 50% EtOAc in PE), the title compound was isolated as a off-white solid (31.1 mg, 86% yield).

^{1}H NMR (500 MHz, $CDCl_3$) δ 7.94 – 7.83 (m, 2H), 7.44 – 7.34 (m, 2H), 4.42 (t, *J* = 9.5 Hz, 2H), 4.05 (t, *J* = 9.5 Hz, 2H).

^{13}C NMR (126 MHz, $CDCl_3$) δ 164.0, 137.6, 129.6, 128.8, 126.4, 67.9, 55.1.

HRMS (ESI) *m/z* calcd for $C_9H_9ClNO^+$ $[(M+H)^+]$ 182.0367, found 182.0365.

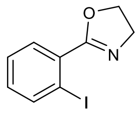

2-(2-iodophenyl)-4,5-dihydrooxazole (6): This compound was prepared according to the General Procedure A, using N-(2-hydroxyethyl)-2-iodobenzamide (58.2 mg, 0.2 mmol), trifluoromethanesulfonic acid (45.0 mg, 27μL, 0.3 mmol). After purification by column chromatography (SiO_2, 20% EtOAc in PE to 50% EtOAc in PE), the title compound was isolated as a white solid (49.7 mg, 91% yield).

^{1}H NMR (500 MHz, $CDCl_3$) δ 7.93 (dd, *J* = 7.9, 1.2 Hz, 1H), 7.62 (dd, *J* = 7.8, 1.8 Hz, 1H), 7.43 – 7.32 (m, 1H), 7.14 – 7.04 (m, 1H), 4.44 (t, *J* = 9.6 Hz, 2H), 4.09 (t, *J* = 9.6 Hz, 2H).

^{13}C NMR (126 MHz, $CDCl_3$) δ 165.0, 140.6, 133.6, 131.7, 130.8, 127.9, 94.7, 67.9, 55.3.

HRMS (ESI) *m/z* calcd for $C_9H_9INO^+$ $[(M+H)^+]$ 273.9723, found 273.9720.

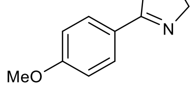

2-(4-methoxyphenyl)-4,5-dihydrooxazole (7): This compound was prepared according to the General Procedure A, using N-(2-hydroxyethyl)-4-methoxybenzamide (39.0 mg, 0.2 mmol), trifluoromethanesulfonic acid (45.0 mg, 27μL, 0.3 mmol). After purification by column chromatography (SiO_2, 30% EtOAc in PE to 70% EtOAc in PE), the title compound was isolated

as a off-white solid (33.8 mg, 95% yield).

^{1}H NMR (500 MHz, $CDCl_3$) δ 7.94 – 7.82 (m, 2H), 6.97 – 6.86 (m, 2H), 4.39 (t, *J* = 9.4 Hz, 2H), 4.01 (t, *J* = 9.4 Hz, 2H), 3.82 (s, 3H).

^{13}C NMR (126 MHz, $CDCl_3$) δ 164.7, 162.2, 130.0, 120.3, 113.8, 67.6, 55.4, 54.8.

HRMS (ESI) *m/z* calcd for $C_{10}H_{12}NO_2^+$ [(M+H)$^+$] 178.0863, found 178.0861.

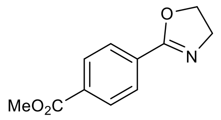

methyl 4-(4,5-dihydrooxazol-2-yl)benzoate (8): This compound was prepared according to the General Procedure A, using methyl 4-((2-hydroxyethyl)carbamoyl)benzoate (44.6 mg, 0.2 mmol), trifluoromethanesulfonic acid (45.0 mg, 27μL, 0.3 mmol). After purification by column chromatography (SiO_2, 20% EtOAc in PE to 60% EtOAc in PE), the title compound was isolated as a white solid (28.5 mg, 70% yield).

^{1}H NMR (500 MHz, $CDCl_3$) δ 8.12 – 8.03 (m, 2H), 7.99 (d, *J* = 8.5 Hz, 2H), 4.44 (t, *J* = 9.5 Hz, 2H), 4.07 (t, *J* = 9.6 Hz, 2H), 3.91 (s, 3H).

^{13}C NMR (126 MHz, $CDCl_3$) δ 166.6, 164.0, 132.6, 131.8, 129.7, 128.3, 68.0, 55.2, 52.4.

HRMS (ESI) *m/z* calcd for $C_{11}H_{12}NO_3^+$ [(M+H)$^+$] 206.0812, found 206.0810.

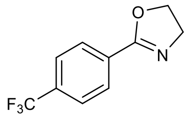

2-(4-(trifluoromethyl)phenyl)-4,5-dihydrooxazole (9): This compound was prepared according to the General Procedure A, using N-(2-hydroxyethyl)-4-(trifluoromethyl)benzamide (46.6 mg, 0.2 mmol), trifluoromethanesulfonic acid (45.0 mg, 27μL, 0.3 mmol). After purification by column chromatography (SiO_2, 20% EtOAc in PE to 60% EtOAc in PE), the title compound was isolated as a white solid (29.5 mg, 69% yield).

^{1}H NMR (500 MHz, $CDCl_3$) δ 8.05 (d, *J* = 8.1 Hz, 2H), 7.66 (d, *J* = 8.2 Hz, 2H), 4.46 (t, *J* = 9.6 Hz, 2H), 4.08 (t, *J* = 9.6 Hz, 2H).

^{13}C NMR (126 MHz, $CDCl_3$) δ 163.6, 133.0 (q, J_{C-F} = 32.8 Hz), 131.2, 128.7, 125.45 (q, J_{C-F} = 3.8 Hz), 123.9 (q, J_{C-F} = 273.4 Hz), 68.0, 55.2.

^{19}F NMR (471 MHz, $CDCl_3$) δ -62.96.

HRMS (ESI) *m/z* calcd for $C_{10}H_9F_3NO^+$ [(M+H)$^+$] 216.0631, found 216.0629.

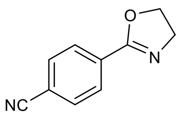

4-(4,5-dihydrooxazol-2-yl)benzonitrile (10): This compound was prepared according to the General Procedure A (60 °C instead of 80 °C), using 4-cyano-N-(2-hydroxyethyl)benzamide (38.0 mg, 0.2 mmol), trifluoromethanesulfonic acid (45.0 mg, 27μL, 0.3 mmol). After purification by column chromatography (SiO_2, 20% EtOAc in PE to 50% EtOAc in PE), the title compound was isolated as a white solid (19.4 mg, 56% yield).

^{1}H NMR (500 MHz, $CDCl_3$) δ 8.12 – 7.97 (m, 2H), 7.76 – 7.64 (m, 2H), 4.47 (t, *J* = 9.6 Hz, 2H), 4.10 (t, *J* = 9.6 Hz, 2H).

^{13}C NMR (126 MHz, $CDCl_3$) δ 163.3, 132.3, 132.0, 128.9, 118.4, 114.9, 68.2, 55.3.

HRMS (ESI) *m/z* calcd for $C_{10}H_9N_2O^+$ $[(M+H)^+]$ 173.0709, found 173.0708.

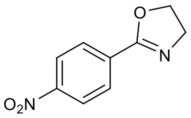

2-(4-nitrophenyl)-4,5-dihydrooxazole (11): This compound was prepared according to the General Procedure A, using N-(2-hydroxyethyl)-4-nitrobenzamide (42.0 mg, 0.2 mmol), trifluoromethanesulfonic acid (45.0 mg, 27μL, 0.3 mmol). After purification by column chromatography (SiO_2, 30% EtOAc in PE to 50% EtOAc in PE), the title compound was isolated as a yellow solid (30.6 mg, 80% yield).

^{1}H NMR (500 MHz, $CDCl_3$) δ 8.34 – 8.20 (m, 2H), 8.19 – 8.03 (m, 2H), 4.48 (t, *J* = 9.6 Hz, 2H), 4.10 (t, *J* = 9.6 Hz, 2H).

^{13}C NMR (126 MHz, $CDCl_3$) δ 163.0, 149.6, 133.6, 129.3, 123.6, 683, 55.3.

HRMS (ESI) *m/z* calcd for $C_9H_9N_2O_3^+$ $[(M+H)^+]$ 193.0608, found 193.0607.

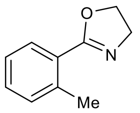

2-(o-tolyl)-4,5-dihydrooxazole (12): This compound was prepared according to the General Procedure A, using N-(2-hydroxyethyl)-2-methylbenzamide (35.8 mg, 0.2 mmol), trifluoromethanesulfonic acid (45.0 mg, 27μL, 0.3 mmol). After purification by column chromatography (SiO_2, 20% EtOAc in PE to 40% EtOAc in PE), the title compound was isolated as a colorless oil (27.3 mg, 85% yield).

^{1}H NMR (500 MHz, CDCl3) δ 7.79 (dd, *J* = 7.7, 1.5 Hz, 1H), 7.40 – 7.29 (m, 1H), 7.25 – 7.18 (m, 2H), 4.42 – 4.34 (m, 2H), 4.08 (t, *J* = 9.5 Hz, 2H), 2.58 (s, 3H).

^{13}C NMR (126 MHz, CDCl3) δ 165.2, 138.8, 131.3, 130.6, 129.9, 127.3, 125.7, 66.9, 55.4, 21.8.

HRMS (ESI) *m/z* calcd for $C_{10}H_{12}NO^+$ $[(M+H)^+]$ 162.0913, found 162.0911.

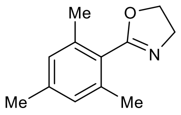

2-mesityl-4,5-dihydrooxazole (13): This compound was prepared according to the General Procedure A, using N-(2-hydroxyethyl)-2,4,6-trimethylbenzamide (41.5 mg, 0.2 mmol), trifluoromethanesulfonic acid (45.0 mg, 27μL, 0.3 mmol). After purification by column chromatography (SiO_2, 10% EtOAc in PE to 30% EtOAc in PE), the title compound was isolated as a white solid (32.0 mg, 85% yield).

^{1}H NMR (500 MHz, $CDCl_3$) δ 6.85 (s, 2H), 4.38 (t, *J* = 9.5 Hz, 2H), 4.08 (t, *J* = 9.5 Hz, 2H), 2.29 (s, 6H), 2.27 (s, 3H).

^{13}C NMR (126 MHz, $CDCl_3$) δ 165.2, 139.2, 137.0, 128.3, 126.1, 67.2, 55.3, 21.3, 19.8.

HRMS (ESI) *m/z* calcd for $C_{12}H_{16}NO^+$ $[(M+H)^+]$ 190.1226, found 190.1224.

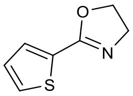

2-(thiophen-2-yl)-4,5-dihydrooxazole (14): This compound was prepared according to the General Procedure A, using N-(2-hydroxyethyl)thiophene-2-carboxamide (34.2 mg, 0.2 mmol), trifluoromethanesulfonic acid (45.0 mg, 27μL, 0.3 mmol). After purification by column chromatography (SiO_2, 20% EtOAc in PE to 40% EtOAc in PE), the title compound was isolated as a white solid (29.4 mg, 96% yield).

^{1}H NMR (500 MHz, $CDCl_3$) δ 7.58 (dd, *J* = 3.8, 1.1 Hz, 1H), 7.43 (dd, *J* = 5.1, 1.2 Hz, 1H), 7.06 (dd, *J* = 5.0, 3.7 Hz, 1H), 4.41 (t, *J* = 9.4 Hz, 2H), 4.02 (t, *J* = 9.4 Hz, 2H).

^{13}C NMR (126 MHz, $CDCl_3$) δ 160.5, 130.4, 130.3, 129.9, 127.7, 68.2, 55.0.

HRMS (ESI) *m/z* calcd for $C_7H_8NOS^+$ $[(M+H)^+]$ 154.0321, found 154.0320.

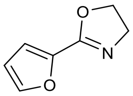

2-(furan-2-yl)-4,5-dihydrooxazole (15): This compound was prepared according to the General Procedure A, using N-(2-hydroxyethyl)furan-2-carboxamide (31.0 mg, 0.2 mmol),

trifluoromethanesulfonic acid (45.0 mg, 27μL, 0.3 mmol). After purification by column chromatography (SiO_2, 30% EtOAc in PE to 50% EtOAc in PE), the title compound was isolated as a yellow solid (20.0 mg, 73% yield).

^{1}H NMR (500 MHz, $CDCl_3$) δ 7.52 (d, *J* = 1.8 Hz, 1H), 6.93 (d, *J* = 3.5 Hz, 1H), 6.46 (dd, *J* = 3.5, 1.8 Hz, 1H), 4.39 (t, *J* = 9.4 Hz, 2H), 4.03 (t, *J* = 9.4 Hz, 2H).

^{13}C NMR (126 MHz, $CDCl_3$) δ 157.1, 145.3, 143.1, 114.3, 111.6, 67.8, 54.9.

HRMS (ESI) *m/z* calcd for $C_7H_8NO_2^+$ [$(M+H)^+$] 138.0550, found 138.0549.

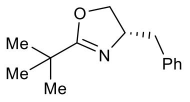

(S)-4-benzyl-2-(tert-butyl)-4,5-dihydrooxazole (16): This compound was prepared according to the General Procedure A, using (S)-N-(1-hydroxy-3-phenylpropan-2-yl)pivalamide (47.1 mg, 0.2 mmol), trifluoromethanesulfonic acid (45.0 mg, 27μL, 0.3 mmol). After purification by column chromatography (SiO_2, 10% EtOAc in PE to 30% EtOAc in PE), the title compound was isolated as a colorless oil (25.4mg, 58% yield).

^{1}H NMR (500 MHz, $CDCl_3$) δ 7.35 – 7.29 (m, 2H), 7.28 – 7.19 (m, 3H), 4.45 – 4.34 (m, 1H), 4.15 (dd, *J* = 9.3, 8.4 Hz, 1H), 4.00 (dd, *J* = 8.5, 6.7 Hz, 1H), 3.12 (dd, *J* = 13.7, 4.5 Hz, 1H), 2.68 (dd, *J* = 13.6, 8.5 Hz, 1H), 1.22 (s, 9H).

^{13}C NMR (126 MHz, $CDCl_3$) δ 174.5, 137.9, 129.6, 128.5, 126.5, 71.5, 66.9, 41.6, 33.2, 27.9.

HRMS (ESI) *m/z* calcd for $C_{14}H_{20}NO^+$ [$(M+H)^+$] 218.1539, found 218.1536.

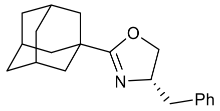

(S)-2-((3S,5S,7S)-adamantan-1-yl)-4-benzyl-4,5-dihydrooxazole (17): This compound was prepared according to the General Procedure A, using (3S,5S,7S)-N-((S)-1-hydroxy-3-phenylpropan-2-yl)adamantane-1-carboxamide (62.7 mg, 0.2 mmol), trifluoromethanesulfonic acid (45.0 mg, 27μL, 0.3 mmol). After purification by column chromatography (SiO_2, 10% EtOAc in PE to 30% EtOAc in PE), the title compound was isolated as a colorless oil (50.1 mg, 85% yield).

^{1}H NMR (500 MHz, $CDCl_3$) δ 7.30 (t, *J* = 7.4 Hz, 2H), 7.21 (dd, *J* = 7.6, 5.9 Hz, 3H), 4.41 – 4.31 (m, 1H), 4.10 (t, *J* = 8.9 Hz, 1H), 3.94 (dd, *J* = 8.5, 6.8 Hz, 1H), 3.11 (dd, *J* = 13.7, 4.6 Hz, 1H), 2.64 (dd, *J* = 13.7, 8.6 Hz, 1H), 2.05 – 1.99 (m, 3H), 1.94 – 1.83 (m, 6H), 1.79 – 1.69 (m, 6H).

^{13}C NMR (126 MHz, $CDCl_3$) δ 173.9, 138.0, 129.6, 128.5, 126.5, 71.1, 66.7, 41.8, 39.6, 36.7, 35.2, 28.0.

HRMS (ESI) *m/z* calcd for $C_{20}H_{26}NO^+$ $[(M+H)^+]$ 296.2009, found 296.2004.

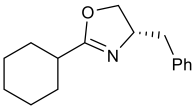

(S)-4-benzyl-2-cyclohexyl-4,5-dihydrooxazole (18): This compound was prepared according to the General Procedure A, using (S)-N-(1-hydroxy-3-phenylpropan-2-yl)cyclohexanecarboxamide (52.3 mg, 0.2 mmol), trifluoromethanesulfonic acid (45.0 mg, 27μL, 0.3 mmol). After purification by column chromatography (SiO_2, 10% EtOAc in PE to 30% EtOAc in PE), the title compound was isolated as a white solid (24.8 mg, 51% yield).

This compound was also prepared according to General Procedure B, using cyclohexanecarboxylic acid (25.6 mg, 0.2 mmol), N-ethynyl-N,4-dimethylbenzenesulfonamide (46.0 mg, 0.22 mmol), (S)-2-amino-3-phenylpropan-1-ol (36.3 mg, 0.24 mmol), trifluoromethanesulfonic acid (45.0 mg, 27μL, 0.3 mmol). After purification by column chromatography (SiO_2, 5% EtOAc in PE to 20% EtOAc in PE), the title compound was isolated as a white solid (25.0 mg, 51% yield).

^1H NMR (500 MHz, $CDCl_3$) δ 7.28 (t, J = 7.4 Hz, 2H), 7.25 – 7.15 (m, 3H), 4.42 – 4.30 (m, 1H), 4.11 (t, J = 8.9 Hz, 1H), 3.92 (dd, J = 8.5, 6.9 Hz, 1H), 3.08 (dd, J = 13.7, 4.8 Hz, 1H), 2.63 (dd, J = 13.7, 8.6 Hz, 1H), 2.30 – 2.21 (m, 1H), 1.94 – 1.85 (m, 2H), 1.79 – 1.72 (m, 2H), 1.69 – 1.62 (m, 1H), 1.45 – 1.35 (m, 2H), 1.31 – 1.21 (m, 3H).

^{13}C NMR (126 MHz, $CDCl_3$) δ 171.5, 138.0, 129.5, 128.6, 126.5, 71.3, 67.0, 41.8, 37.5, 30.0, 29.9, 26.0, 25.8, 25.7.

HRMS (ESI) *m/z* calcd for $C_{16}H_{22}NO^+$ $[(M+H)^+]$ 244.1696, found 244.1692.

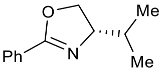

(S)-4-isopropyl-2-phenyl-4,5-dihydrooxazole (19): This compound was prepared according to the General Procedure A, using (S)-N-(1-hydroxy-3-methylbutan-2-yl)benzamide (41.5 mg, 0.2 mmol), trifluoromethanesulfonic acid (45.0 mg, 27μL, 0.3 mmol). After purification by column chromatography (SiO_2, 5% EtOAc in PE to 20% EtOAc in PE), the title compound was isolated as a colorless oil (33.2 mg, 88% yield).

^{1}H NMR (500 MHz, $CDCl_3$) δ 8.14 – 7.90 (m, 2H), 7.49 – 7.44 (m, 1H), 7.44 – 7.33 (m, 2H), 4.46 – 4.35 (m, 1H), 4.21 – 4.05 (m, 2H), 1.93 – 1.81 (m, 1H), 1.03 (d, *J* = 6.8 Hz, 3H), 0.92 (d, *J* = 6.8 Hz, 3H).

^{13}C NMR (126 MHz, $CDCl_3$) δ 163.5, 131.3, 128.4, 128.4, 128.0, 72.7, 70.2, 32.9, 19.1, 18.2.

HRMS (ESI) *m/z* calcd for $C_{12}H_{16}NO^+$ $[(M+H)^+]$ 190.1226, found 190.1225.

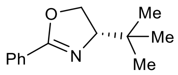

(S)-4-(tert-butyl)-2-phenyl-4,5-dihydrooxazole (20): This compound was prepared according to the General Procedure A, using (S)-N-(1-hydroxy-3,3-dimethylbutan-2-yl)benzamide (44.3 mg, 0.2 mmol), trifluoromethanesulfonic acid (45.0 mg, 27μL, 0.3 mmol). After purification by column chromatography (SiO_2, 5% EtOAc in PE to 20% EtOAc in PE), the title compound was isolated as a colorless oil (31.0 mg, 76% yield).

^{1}H NMR (500 MHz, $CDCl_3$) δ 8.02 – 7.91 (m, 2H), 7.49 – 7.43 (m, 1H), 7.43 – 7.36 (m, 2H), 4.34 (dd, *J* = 10.1, 8.6 Hz, 1H), 4.24 (dd, *J* = 8.6, 7.7 Hz, 1H), 4.05 (dd, *J* = 10.1, 7.7 Hz, 1H), 0.96 (s, 9H).

^{13}C NMR (126 MHz, $CDCl_3$) δ 163.4, 131.3, 128.4, 128.4, 128.1, 76.3, 68.8, 34.2, 26.0.

HRMS (ESI) *m/z* calcd for $C_{13}H_{18}NO^+$ $[(M+H)^+]$ 204.1383, found 204.1380.

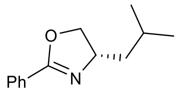

(S)-4-isobutyl-2-phenyl-4,5-dihydrooxazole (21): This compound was prepared according to the General Procedure A, using (S)-N-(1-hydroxy-4-methylpentan-2-yl)benzamide (44.3 mg, 0.2 mmol), trifluoromethanesulfonic acid (45.0 mg, 27μL, 0.3 mmol). After purification by column chromatography (SiO_2, 5% EtOAc in PE to 20% EtOAc in PE), the title compound was isolated as a colorless oil (33.0 mg, 81% yield).

^{1}H NMR (500 MHz, $CDCl_3$) δ 8.00 – 7.89 (m, 2H), 7.48 – 7.43 (m, 1H), 7.42 – 7.36 (m, 2H), 4.50 (dd, *J* = 9.3, 8.0 Hz, 1H), 4.37 – 4.28 (m, 1H), 3.99 (t, *J* = 8.0 Hz, 1H), 1.87 – 1.76 (m, 1H), 1.76 – 1.67 (m, 1H), 1.42 – 1.34 (m, 1H), 0.98 (t, *J* = 7.0 Hz, 6H).

^{13}C NMR (126 MHz, $CDCl_3$) δ 163.4, 131.3, 128.4, 128.3, 128.1, 73.2, 65.3, 45.7, 25.6, 23.1, 22.8.

HRMS (ESI) *m/z* calcd for $C_{13}H_{18}NO^+$ $[(M+H)^+]$ 204.1383, found 204.1381.

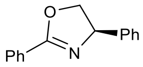

(R)-2,4-diphenyl-4,5-dihydrooxazole (22): This compound was prepared according to the General Procedure A, using (R)-N-(2-hydroxy-1-phenylethyl)benzamide (48.3mg, 0.2 mmol), trifluoromethanesulfonic acid (45.0 mg, 27μL, 0.3 mmol). After purification by column chromatography (SiO_2, 10% EtOAc in PE to 30% EtOAc in PE), the title compound was isolated as a yellow oil (40.6 mg, 91% yield).

^{1}H NMR (500 MHz, $CDCl_3$) δ 8.11 – 8.02 (m, 2H), 7.54 – 7.50 (m, 1H), 7.45 (dd, *J* = 8.3, 6.8 Hz, 2H), 7.39 – 7.35 (m, 2H), 7.34 – 7.27 (m, 3H), 5.40 (dd, *J* = 10.2, 8.2 Hz, 1H), 4.80 (dd, *J* = 10.1, 8.3 Hz, 1H), 4.29 (t, *J* = 8.3 Hz, 1H).

^{13}C NMR (126 MHz, $CDCl_3$) δ 164.9, 142.5, 131.7, 128.9, 128.6, 128.5, 127.8, 127.7, 126.9, 75.0, 70.2.

HRMS (ESI) *m/z* calcd for $C_{15}H_{14}NO^+$ [$(M+H)^+$] 224.1070, found 224.1067.

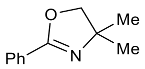

4,4-dimethyl-2-phenyl-4,5-dihydrooxazole (23): This compound was prepared according to the General Procedure A, using N-(1-hydroxy-2-methylpropan-2-yl)benzamide (38.6 mg, 0.2 mmol), trifluoromethanesulfonic acid (45.0 mg, 27μL, 0.3 mmol). After purification by column chromatography (SiO_2, 20% EtOAc in PE to 40% EtOAc in PE), the title compound was isolated as a colorless oil (20.1 mg, 57% yield).

^{1}H NMR (500 MHz, $CDCl_3$) δ 7.99 – 7.90 (m, 2H), 7.49 – 7.43 (m, 1H), 7.43 – 7.36 (m, 2H), 4.10 (s, 2H), 1.38 (s, 6H).

^{13}C NMR (126 MHz, $CDCl_3$) δ 162.3, 131.3, 128.4, 128.4, 128.1, 79.3, 67.7, 28.5.

HRMS (ESI) *m/z* calcd for $C_{11}H_{14}NO^+$ [$(M+H)^+$] 176.1070, found 176.1068.

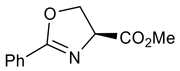

methyl (S)-2-phenyl-4,5-dihydrooxazole-4-carboxylate (24): This compound was prepared according to the General Procedure A, using methyl benzoyl-L-serinate (44.6 mg, 0.2 mmol), trifluoromethanesulfonic acid (45.0 mg, 27μL, 0.3 mmol). After purification by column chromatography (SiO_2, 20% EtOAc in PE to 40% EtOAc in PE), the title compound was isolated as a yellow oil (24.0 mg, 59% yield).

^{1}H NMR (500 MHz, $CDCl_3$) δ 8.03 – 7.93 (m, 2H), 7.55 – 7.46 (m, 1H), 7.45 – 7.37 (m, 2H), 4.95 (dd, *J* = 10.6, 8.0 Hz, 1H), 4.69 (dd, *J* = 8.7, 8.0 Hz, 1H), 4.59 (dd, *J* = 10.6, 8.7 Hz, 1H), 3.81 (s,

3H).

^{13}C NMR (126 MHz, $CDCl_3$) δ 171.8, 166.5, 132.0, 128.7, 128.5, 127.0, 69.7, 68.7, 52.9.

HRMS (ESI) *m/z* calcd for $C_{11}H_{12}NO_3^+$ $[(M+H)^+]$ 206.0812, found 206.0810.

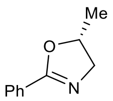

(S)-5-methyl-2-phenyl-4,5-dihydrooxazole (25): This compound was prepared according to the General Procedure A, using(S)-N-(2-hydroxypropyl)benzamide (35.8 mg, 0.2 mmol), trifluoromethanesulfonic acid (45.0 mg, 27μL, 0.3 mmol). After purification by column chromatography (SiO_2, 20% EtOAc in PE to 50% EtOAc in PE), the title compound was isolated as a colorless oil (19.3 mg, 60% yield).

^{1}H NMR (500 MHz, $CDCl_3$) δ 8.01 – 7.87 (m, 2H), 7.50 – 7.43 (m, 1H), 7.43 – 7.36 (m, 2H), 4.93 – 4.77 (m, 1H), 4.14 (dd, *J* = 14.4, 9.4 Hz, 1H), 3.61 (dd, *J* = 14.4, 7.4 Hz, 1H), 1.42 (d, *J* = 6.3 Hz, 3H).

^{13}C NMR (126 MHz, $CDCl_3$) δ 164.1, 131.4, 128.4, 128.2, 128.1, 76.4, 61.6, 21.3.

HRMS (ESI) *m/z* calcd for $C_{10}H_{12}NO^+$ $[(M+H)^+]$ 162.0913, found 162.0911.

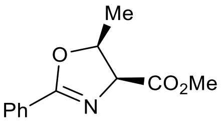

methyl (4R,5R)-5-methyl-2-phenyl-4,5-dihydrooxazole-4-carboxylate (26): This compound was prepared according to the General Procedure A, using methyl benzoyl-L-threoninate (47.5 mg, 0.2 mmol), trifluoromethanesulfonic acid (45.0 mg, 27μL, 0.3 mmol). After purification by column chromatography (SiO_2, 20% EtOAc in PE to 40% EtOAc in PE), the title compound was isolated as a colorless oil (19.1 mg, 44% yield).

^{1}H NMR (500 MHz, $CDCl_3$) δ 7.98 (dd, *J* = 8.3, 1.4 Hz, 2H), 7.49 (t, *J* = 7.4 Hz, 1H), 7.40 (dd, *J* = 8.3, 7.0 Hz, 2H), 5.04 – 4.93 (m, 1H), 4.46 (d, *J* = 7.4 Hz, 1H), 3.80 (s, 3H), 1.53 (d, *J* = 6.3 Hz, 3H).

^{13}C NMR (126 MHz, $CDCl_3$) δ 171.7, 165.7, 131.9, 128.7, 128.5, 127.4, 79.0, 75.3, 52.8, 21.1.

HRMS (ESI) *m/z* calcd for $C_{12}H_{14}NO_3^+$ $[(M+H)^+]$ 220.0968, found 220.0966.

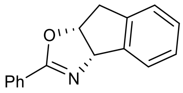

(3aS,8aR)-2-phenyl-3a,8a-dihydro-8H-indeno[1,2-d]oxazole (27): This compound was prepared according to the General Procedure A, using N-((1S,2R)-2-hydroxy-2,3-dihydro-1H-inden-1-yl)benzamide (50.7 mg, 0.2 mmol), trifluoromethanesulfonic acid (45.0 mg, 27μL, 0.3 mmol). After purification by column chromatography (SiO_2, 10% EtOAc in PE to 30% EtOAc in PE), the title compound was isolated as a yellow solid (29.1 mg, 62% yield).

^{1}H NMR (500 MHz, $CDCl_3$) δ 8.00 – 7.89 (m, 2H), 7.62 – 7.55 (m, 1H), 7.47 – 7.40 (m, 1H), 7.37 (dd, *J* = 8.3, 6.8 Hz, 2H), 7.31 – 7.24 (m, 3H), 5.74 (d, *J* = 7.9 Hz, 1H), 5.53 – 5.45 (m, 1H), 3.50 (dd, *J* = 17.9, 6.8 Hz, 1H), 3.37 (dd, *J* = 17.9, 1.8 Hz, 1H).

^{13}C NMR (126 MHz, $CDCl_3$) δ 164.1, 142.1, 139.9, 131.4, 128.6, 128.5, 128.3, 128.0, 127.6, 125.7, 125.4, 76.9, 39.9.

HRMS (ESI) *m/z* calcd for $C_{16}H_{14}NO^+$ $[(M+H)^+]$ 236.1070, found 236.1067.

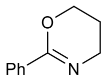

2-phenyl-5,6-dihydro-4H-1,3-oxazine (28): This compound was prepared according to the General Procedure A, usingN-(3-hydroxypropyl)benzamide (35.8 mg, 0.2 mmol), trifluoromethanesulfonic acid (45.0 mg, 27μL, 0.3 mmol). After purification by column chromatography (SiO_2, 20% EtOAc in PE to 50% EtOAc in PE), the title compound was isolated as a yellow oil (16.8 mg, 52% yield).

^{1}H NMR (500 MHz, $CDCl_3$) δ 7.93 – 7.86 (m, 2H), 7.42 – 7.39 (m, 1H), 7.38 – 7.32 (m, 2H), 4.36 (t, *J* = 5.4 Hz, 2H), 3.60 (t, *J* = 5.9 Hz, 2H), 2.01 – 1.96 (m, 2H).

^{13}C NMR (126 MHz, $CDCl_3$) δ 155.8, 134.2, 130.4, 128.1, 127.0, 65.3, 42.8, 22.1.

HRMS (ESI) *m/z* calcd for $C_{10}H_{12}NO^+$ $[(M+H)^+]$ 162.0913, found 162.0912.

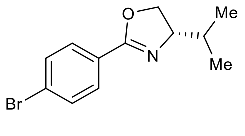

(S)-2-(4-bromophenyl)-4-isopropyl-4,5-dihydrooxazole (29): This compound was prepared according to the General Procedure B, using 4-bromobenzoic acid (40.2 mg, 0.2 mmol), N-ethynyl-N,4-dimethylbenzenesulfonamide (46.0 mg, 0.22 mmol), (S)-2-amino-3-methylbutan-1-ol (24.8 mg, 0.24 mmol), trifluoromethanesulfonic acid (45.0 mg, 27μL, 0.3 mmol). After purification by column chromatography (SiO_2, 5% EtOAc in PE to 20% EtOAc in PE), the title compound was isolated as a white solid (42.6 mg, 80% yield).

^{1}H NMR (500 MHz, $CDCl_3$) δ 7.86 – 7.76 (m, 2H), 7.53 (d, *J* = 8.3 Hz, 2H), 4.40 (dd, *J* = 9.1, 7.9 Hz, 1H), 4.17 – 4.04 (m, 2H), 1.90 – 1.79 (m, 1H), 1.01 (d, *J* = 6.8 Hz, 3H), 0.91 (d, *J* = 6.8 Hz, 3H).

^{13}C NMR (126 MHz, $CDCl_3$) δ 162.8, 131.7, 129.9, 127.0, 126.0, 72.8, 70.4, 32.9, 19.0, 18.2.

HRMS (ESI) *m/z* calcd for $C_{12}H_{15}BrNO^+$ [(M+H)$^+$] 268.0332, found 268.0328.

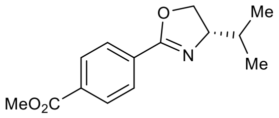

methyl (S)-4-(4-isopropyl-4,5-dihydrooxazol-2-yl)benzoate (30): This compound was prepared according to the General Procedure B, using 4-(methoxycarbonyl)benzoic acid (36.0 mg, 0.2 mmol), N-ethynyl-N,4-dimethylbenzenesulfonamide (46.0 mg, 0.22 mmol), (S)-2-amino-3-methylbutan-1-ol (24.8 mg, 0.24 mmol), trifluoromethanesulfonic acid (45.0 mg, 27μL, 0.3 mmol). After purification by column chromatography (SiO_2, 10% EtOAc in PE to 30% EtOAc in PE), the title compound was isolated as a white solid (31.1 mg, 62% yield).

^{1}H NMR (500 MHz, $CDCl_3$) δ 8.05 (d, *J* = 8.6 Hz, 2H), 8.00 (d, *J* = 8.5 Hz, 2H), 4.46 – 4.38 (m, 1H), 4.18 – 4.08 (m, 2H), 3.92 (s, 3H), 1.90 – 1.82 (m, 1H), 1.02 (d, *J* = 6.7 Hz, 3H), 0.92 (d, *J* = 6.8 Hz, 3H).

^{13}C NMR (126 MHz, $CDCl_3$) δ 166.6, 162.7, 132.4, 132.1, 129.6, 128.3, 127.4, 72.9, 70.5, 52.4, 32.9, 19.0, 18.2.

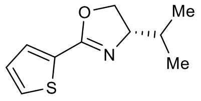

(S)-4-isopropyl-2-(thiophen-2-yl)-4,5-dihydrooxazole (31): This compound was prepared according to the General Procedure B, using thiophene-2-carboxylic acid (25.6 mg, 0.2 mmol), N-ethynyl-N,4-dimethylbenzenesulfonamide (46.0 mg, 0.22 mmol), (S)-2-amino-3-methylbutan-1-ol (24.8 mg, 0.24 mmol), trifluoromethanesulfonic acid (45.0 mg, 27μL, 0.3 mmol). After purification by column chromatography (SiO_2, 10% EtOAc in PE to 30% EtOAc in PE), the title compound was isolated as a colorless oil (29.1mg, 75% yield).

^{1}H NMR (500 MHz, $CDCl_3$) δ 7.59 (dd, *J* = 3.7, 1.2 Hz, 1H), 7.42 (dd, *J* = 5.0, 1.3 Hz, 1H), 7.06 (dd, *J* = 5.0, 3.7 Hz, 1H), 4.38 (dd, *J* = 9.0, 7.6 Hz, 1H), 4.16 – 4.05 (m, 2H), 1.92 – 1.81 (m, 1H), 1.01 (d, *J* = 6.7 Hz, 3H), 0.91 (d, *J* = 6.8 Hz, 3H).

^{13}C NMR (126 MHz, $CDCl_3$) δ 159.2, 130.6, 130.3, 129.7, 127.7, 72.8, 70.5, 32.8, 19.1, 18.1.

HRMS (ESI) *m/z* calcd for $C_{10}H_{14}NOS^+$ $[(M+H)^+]$ 196.0791, found 196.0789.

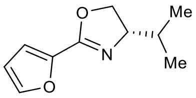

(S)-2-(furan-2-yl)-4-isopropyl-4,5-dihydrooxazole (32): This compound was prepared according to the General Procedure B, using furan-2-carboxylic acid (22.4 mg, 0.2 mmol), N-ethynyl-N,4-dimethylbenzenesulfonamide (46.0 mg, 0.22 mmol), (S)-2-amino-3-methylbutan-1-ol (24.8 mg, 0.24 mmol), trifluoromethanesulfonic acid (45.0 mg, 27μL, 0.3 mmol). After purification by column chromatography (SiO_2, 10% EtOAc in PE to 30% EtOAc in PE), the title compound was isolated as a yellow oil (24.1 mg, 67% yield).

^{1}H NMR (500 MHz, $CDCl_3$) δ 7.51 (dd, *J* = 1.9, 0.8 Hz, 1H), 6.92 (dd, *J* = 3.5, 0.8 Hz, 1H), 6.46 (dd, *J* = 3.5, 1.8 Hz, 1H), 4.43 – 4.33 (m, 1H), 4.11 – 4.05 (m, 2H), 1.89 – 1.79 (m, 1H), 1.01 (d, *J* = 6.8 Hz, 3H), 0.91 (d, *J* = 6.8 Hz, 3H).

^{13}C NMR (126 MHz, $CDCl_3$) δ 155.9, 145.2, 143.1, 114.3, 111.6, 72.8, 70.4, 32.8, 19.1, 18.3.

HRMS (ESI) *m/z* calcd for $C_{10}H_{14}NO_2^+$ $[(M+H)^+]$ 180.1019, found 180.1017.

NMR Data

2, ^{1}H NMR (500 MHz, $CDCl_3$)

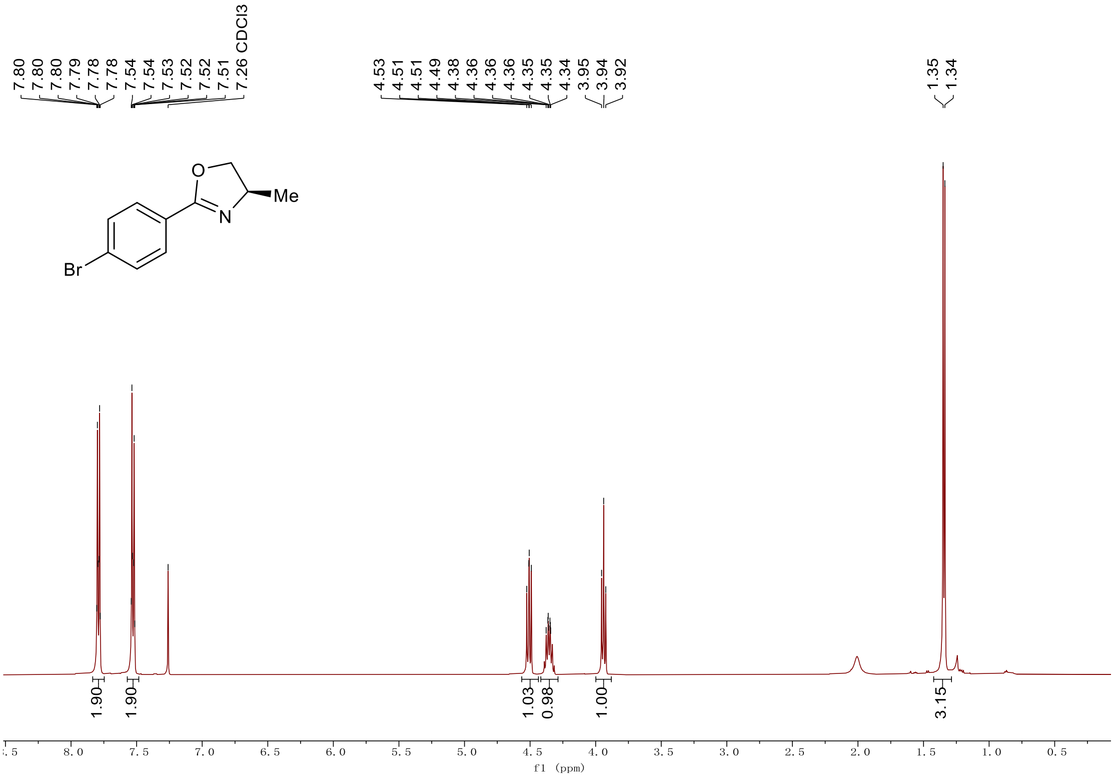

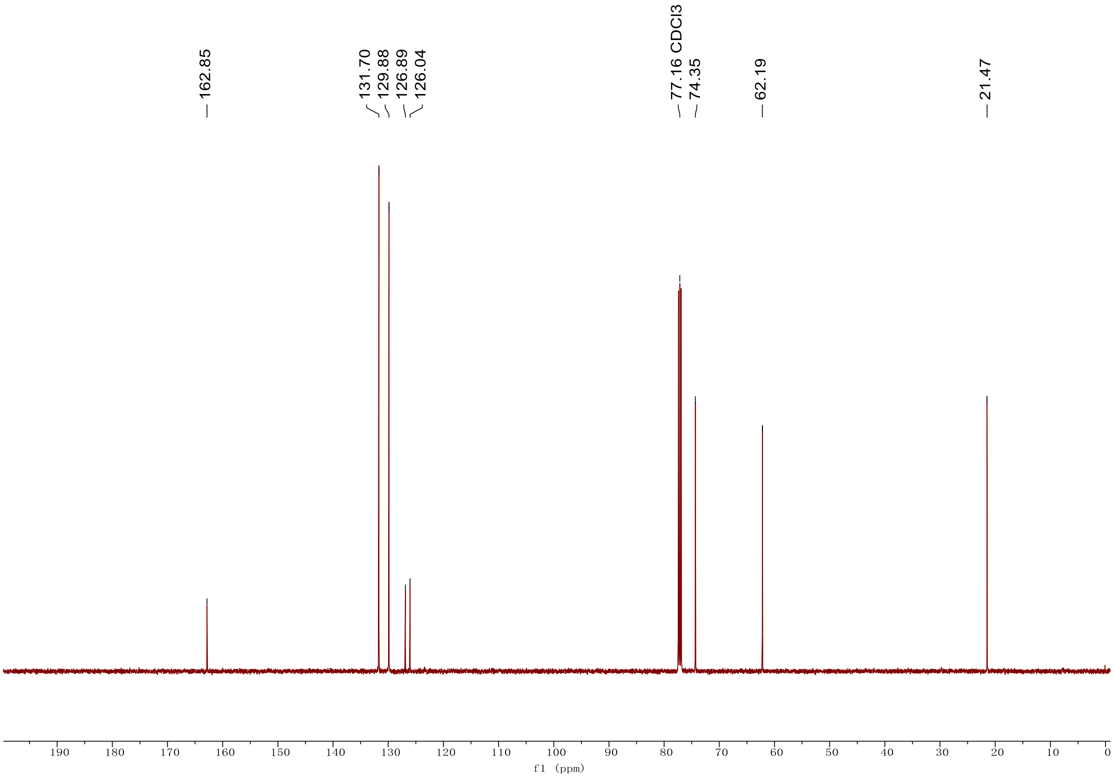

3, ^{1}H NMR (500 MHz, $CDCl_3$)

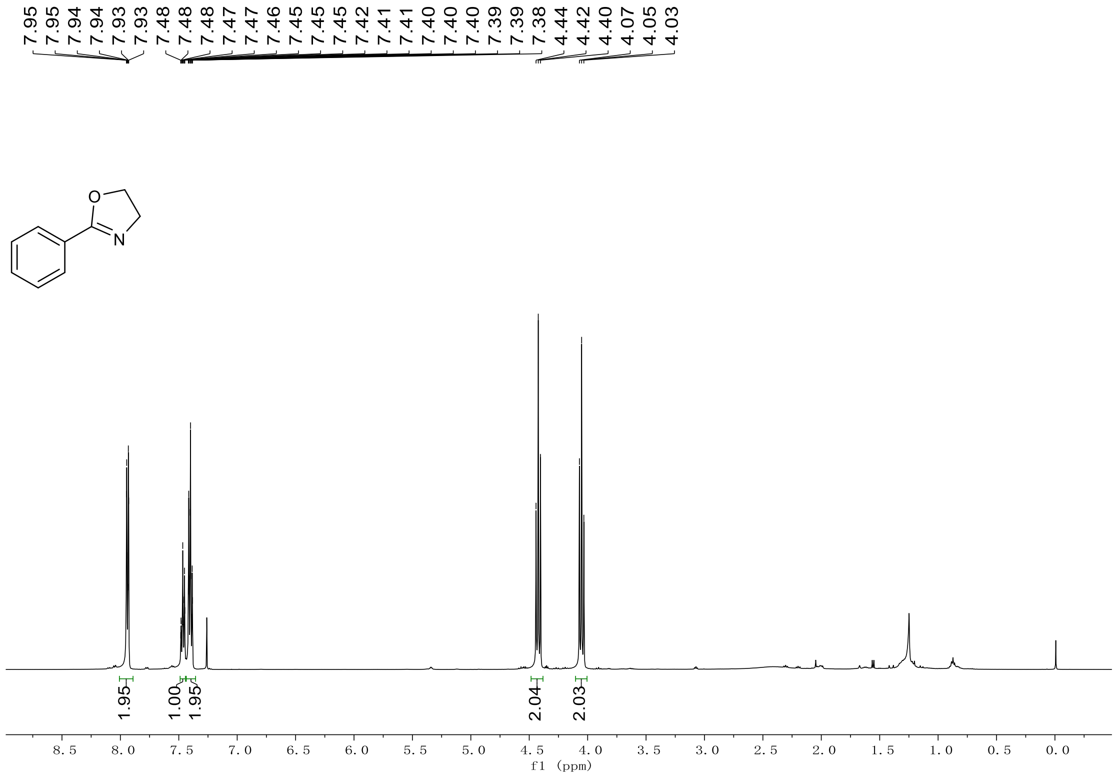

3, ^{13}C NMR (126 MHz, $CDCl_3$)

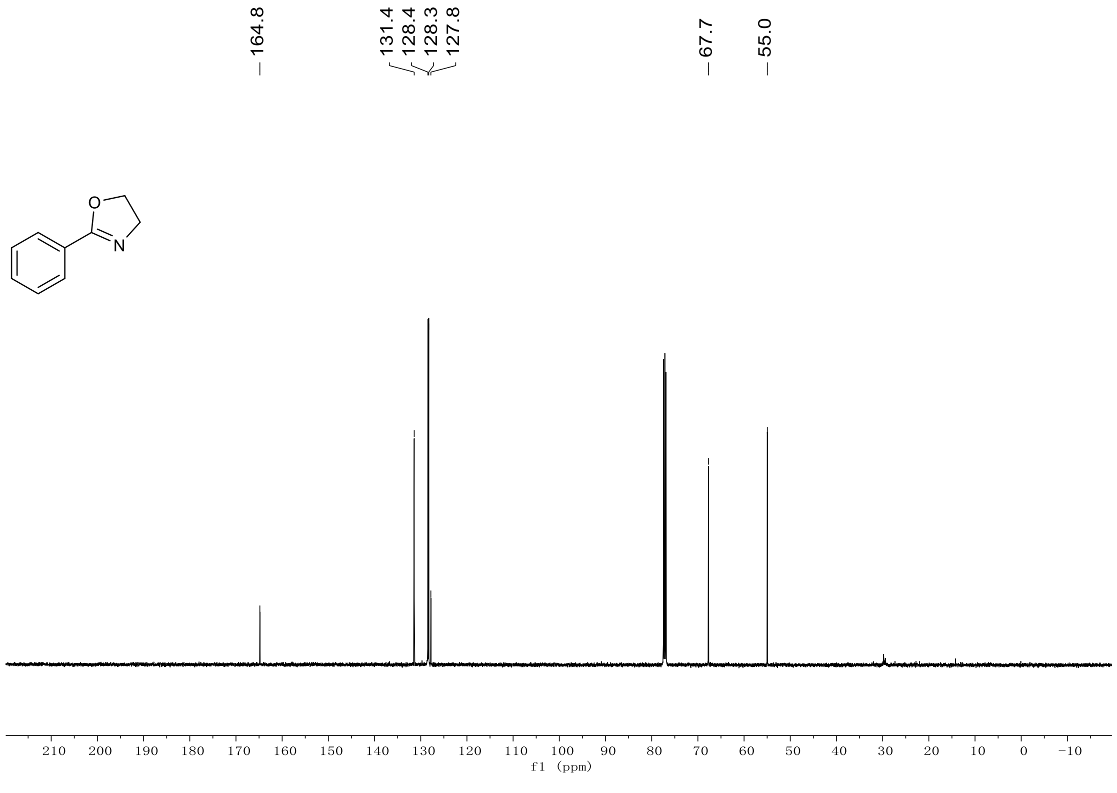

4, ^{1}H NMR (500 MHz, $CDCl_3$)

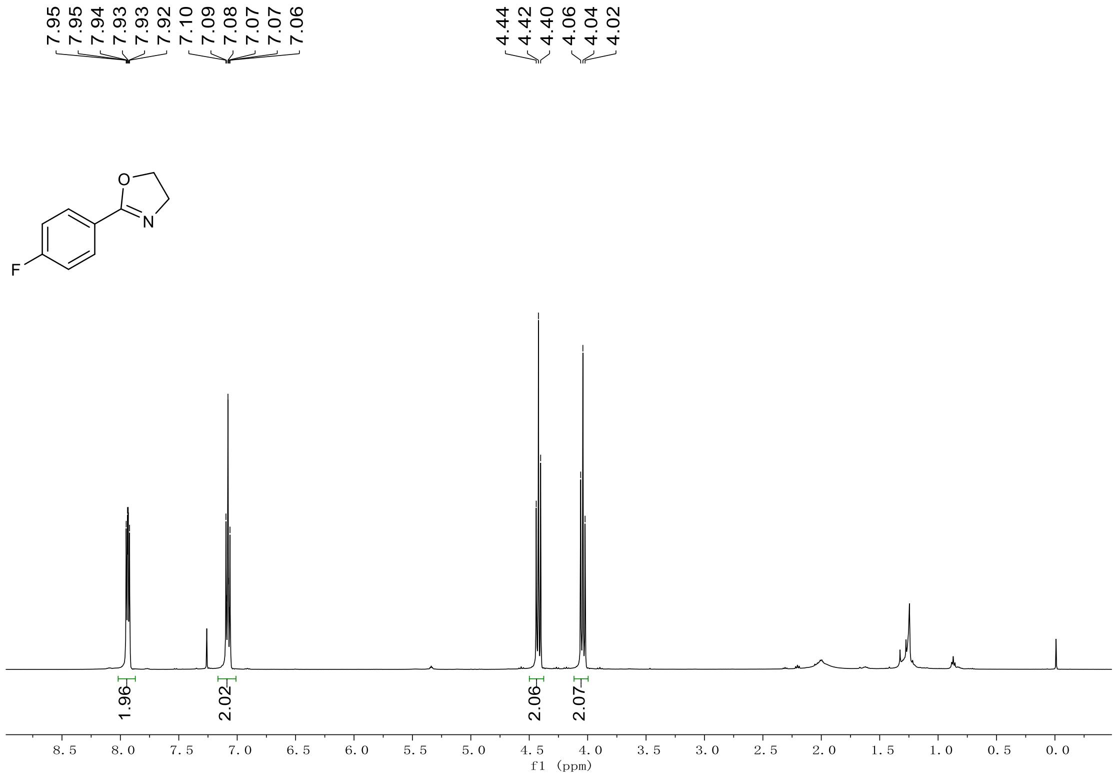

4, ^{13}C NMR (126 MHz, $CDCl_3$)

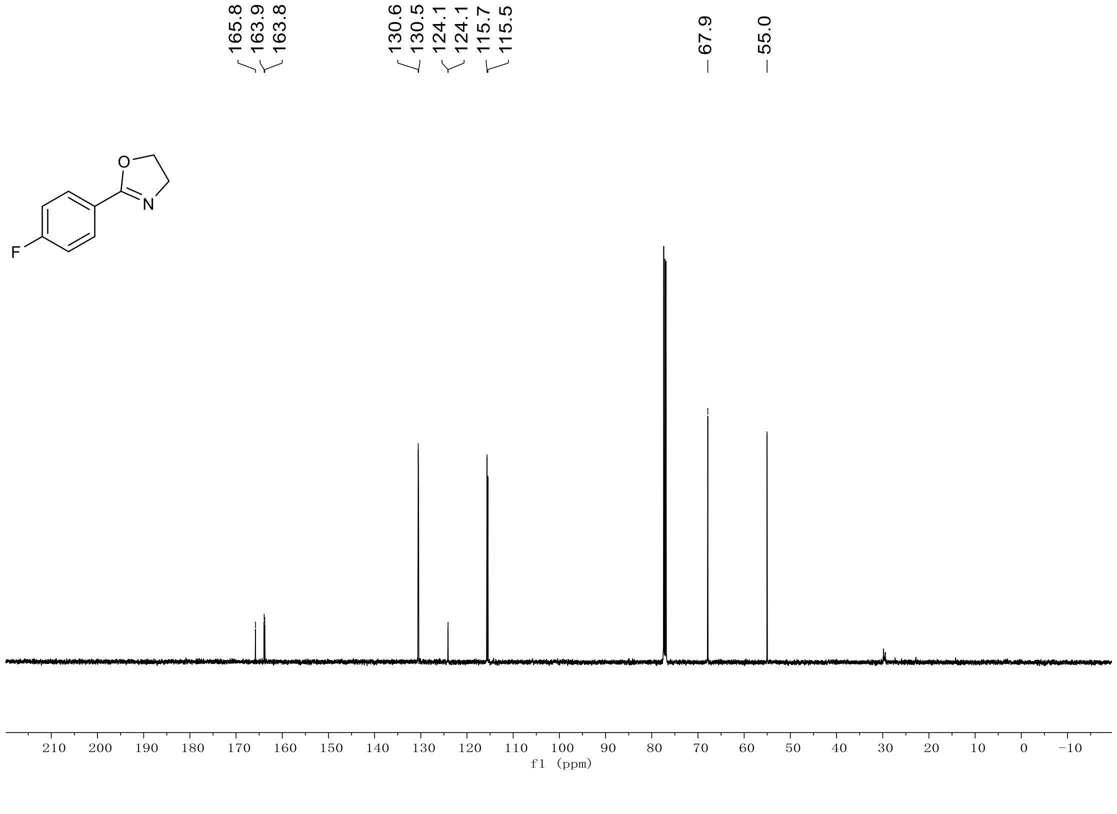

4, ^{19}F NMR (471 MHz, $CDCl_3$)

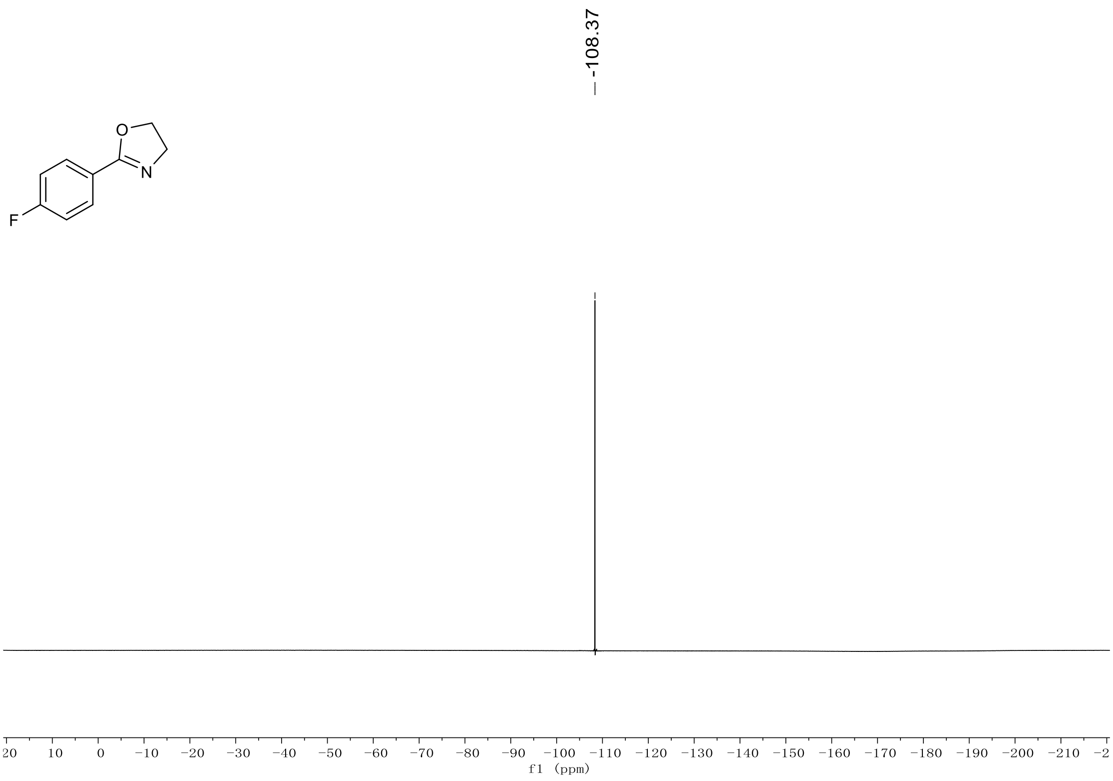

5, ^{1}H NMR (500 MHz, $CDCl_3$)

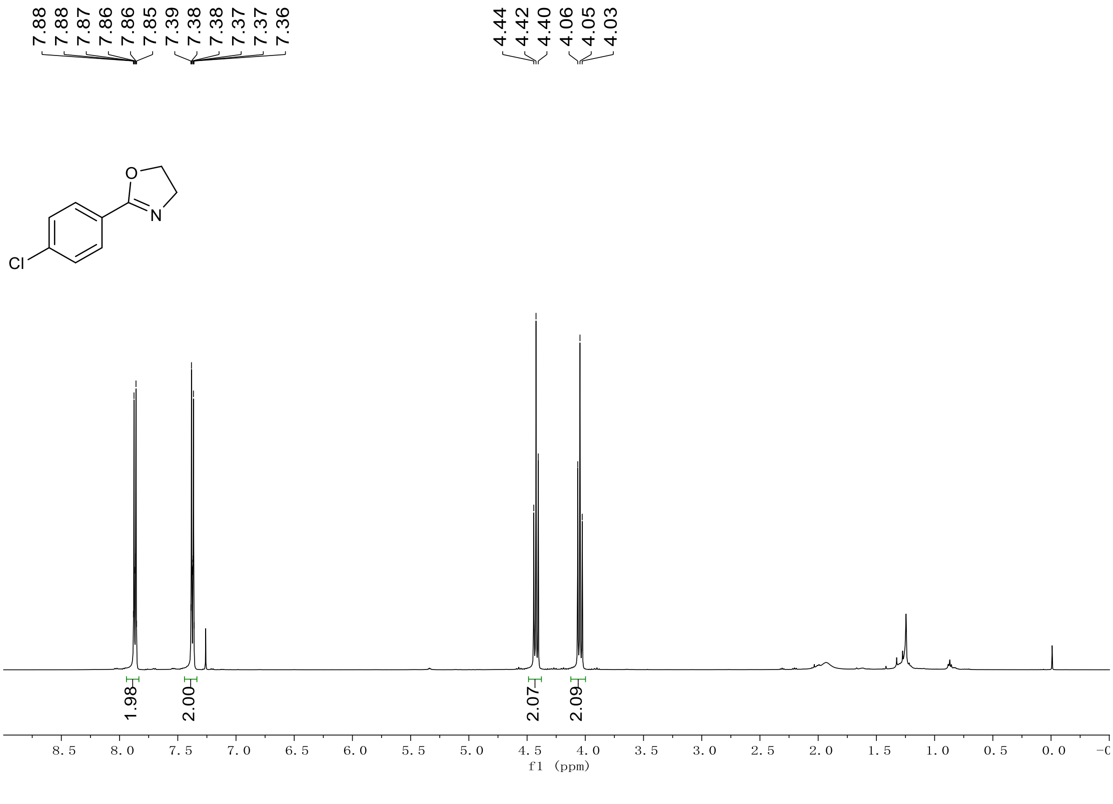

5, ^{13}C NMR (126 MHz, $CDCl_3$)

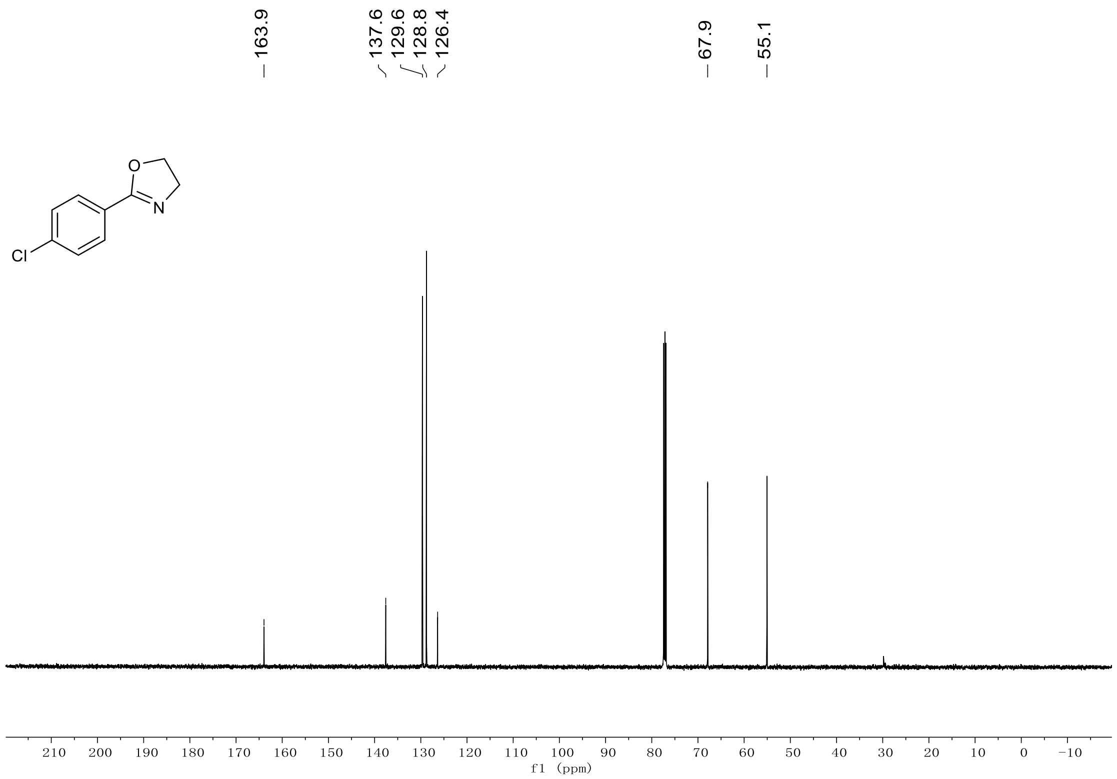

6, ^{1}H NMR (500 MHz, $CDCl_3$)

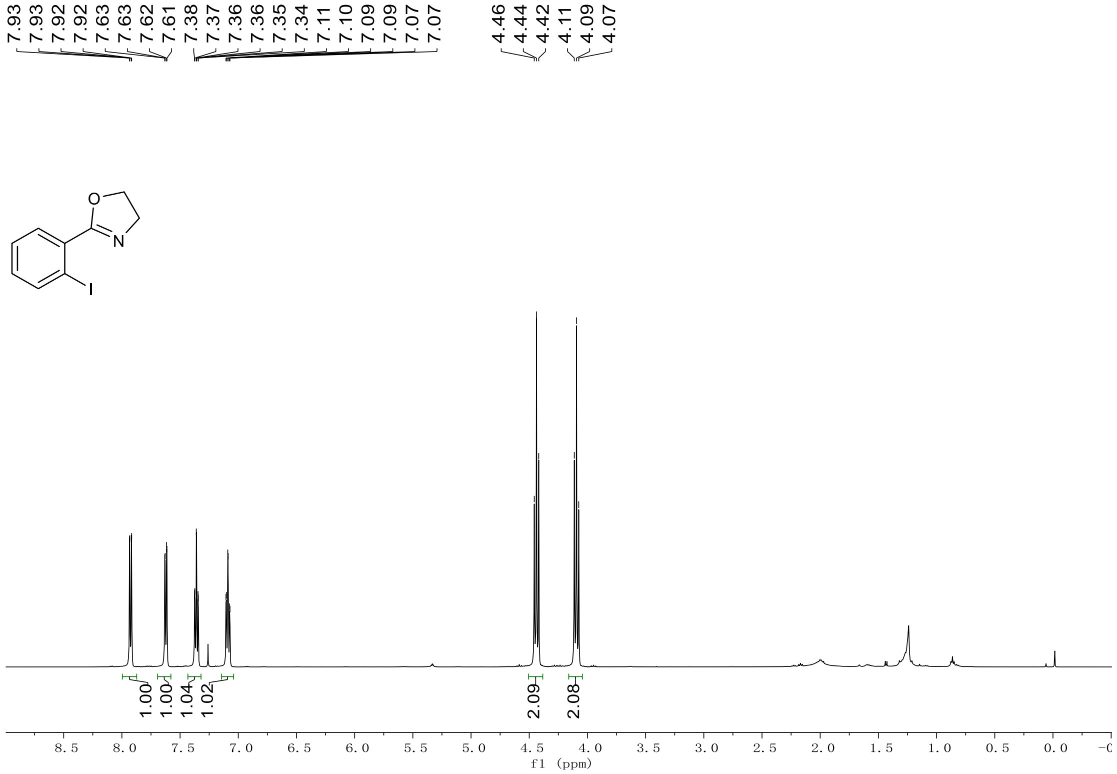

6, ^{13}C NMR (126 MHz, $CDCl_3$)

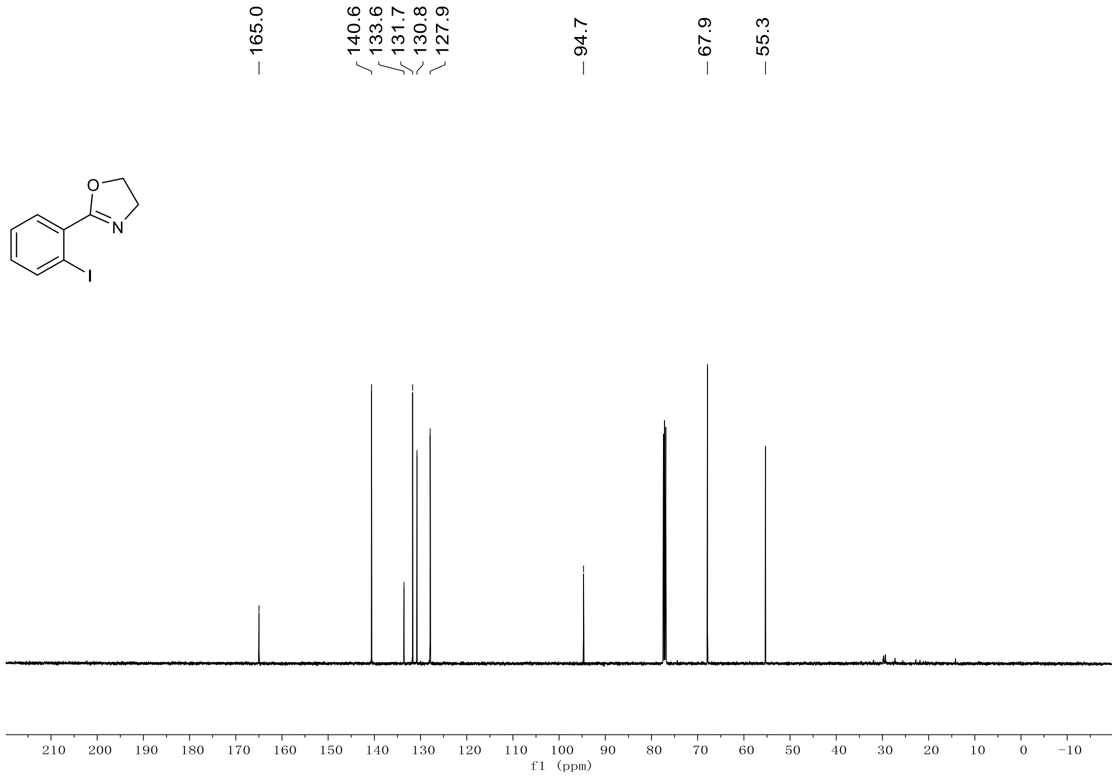

7, ^{1}H NMR (500 MHz, $CDCl_3$)

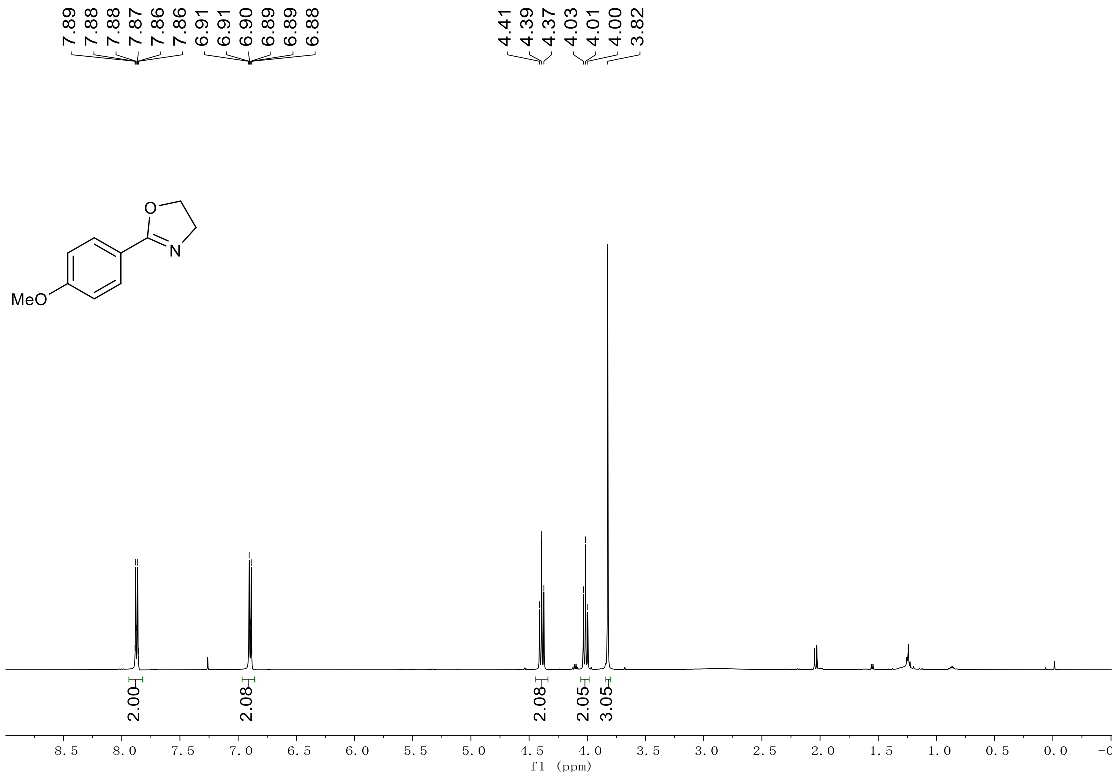

7, ^{13}C NMR (126 MHz, $CDCl_3$)

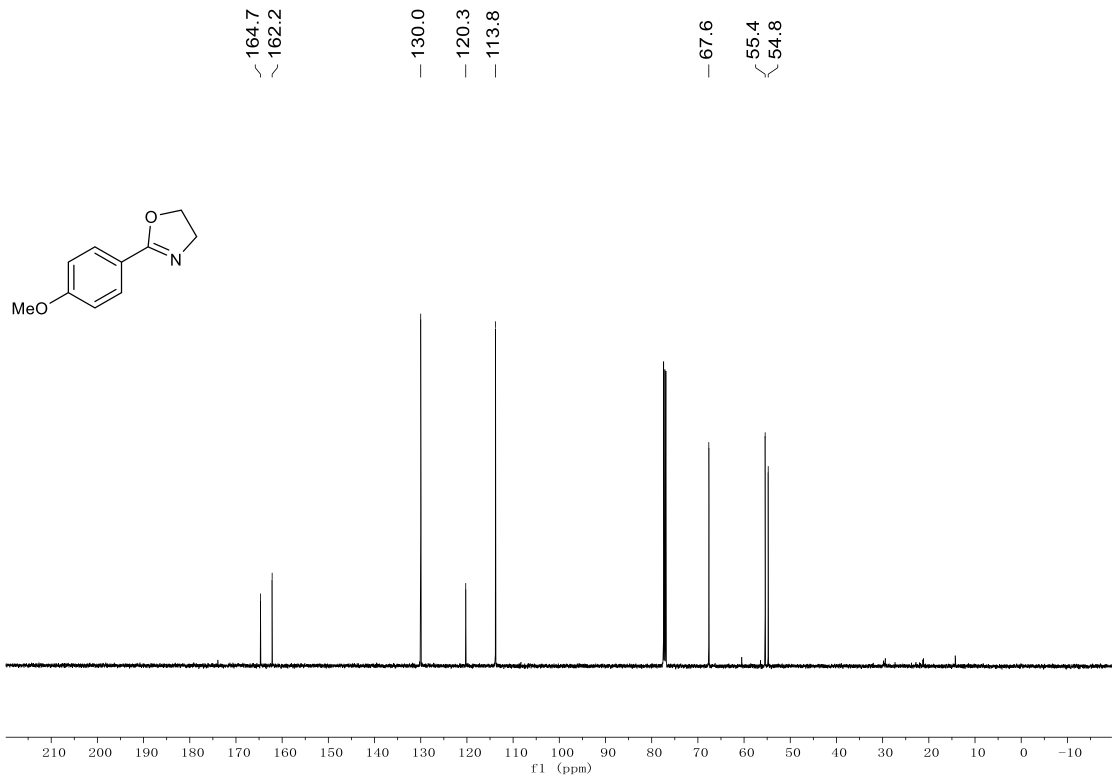

8, ^{1}H NMR (500 MHz, $CDCl_3$)

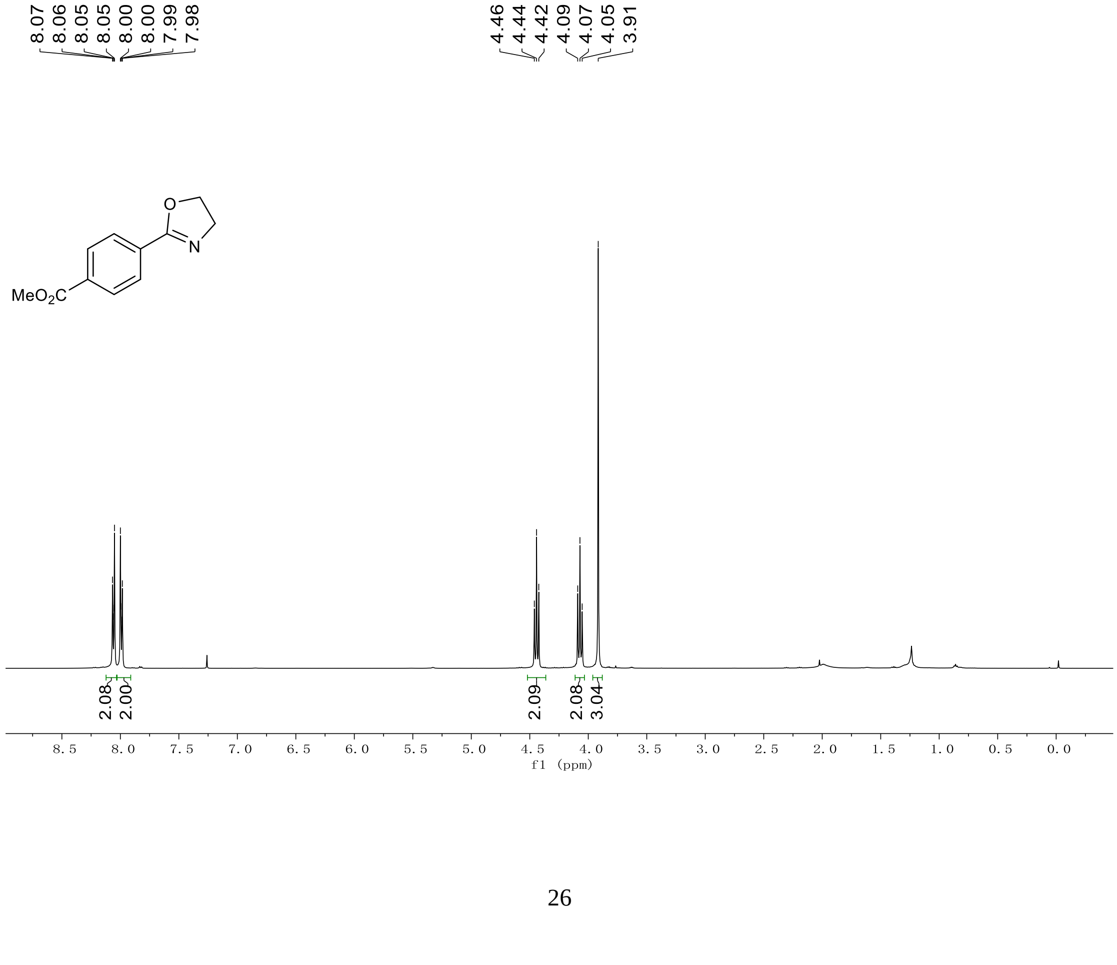

8, ^{13}C NMR (126 MHz, $CDCl_3$)

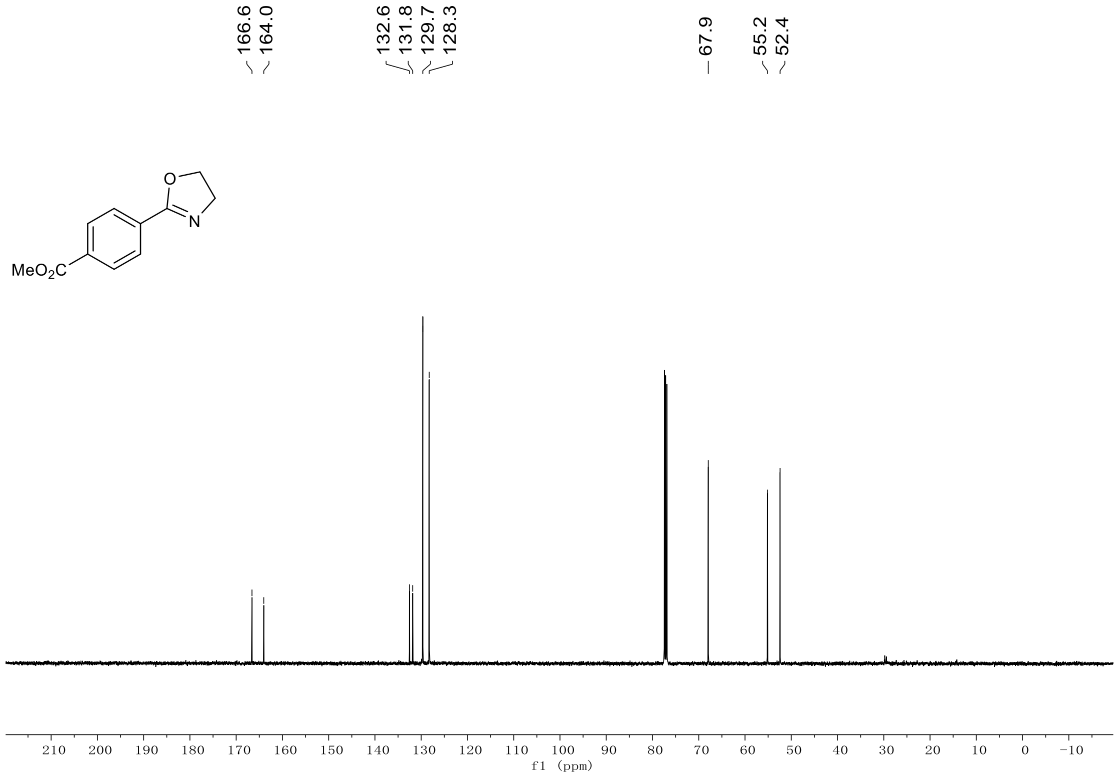

9, ^{1}H NMR (500 MHz, $CDCl_3$)

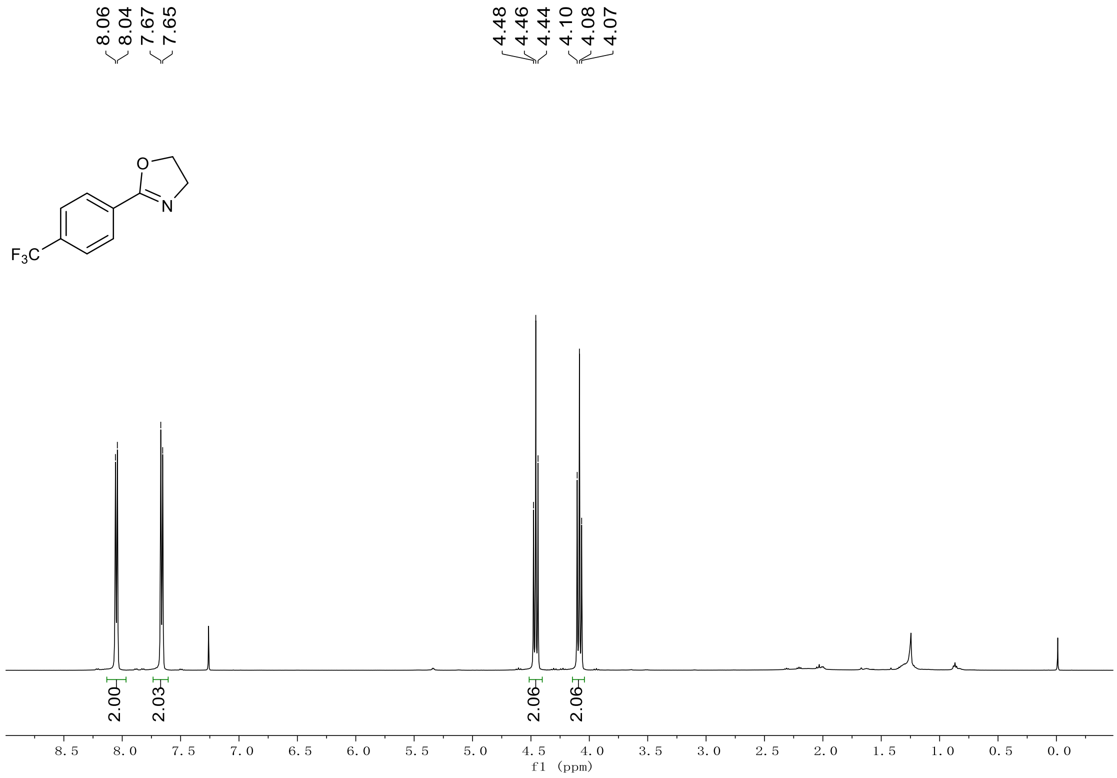

9, ^{13}C NMR (126 MHz, $CDCl_3$)

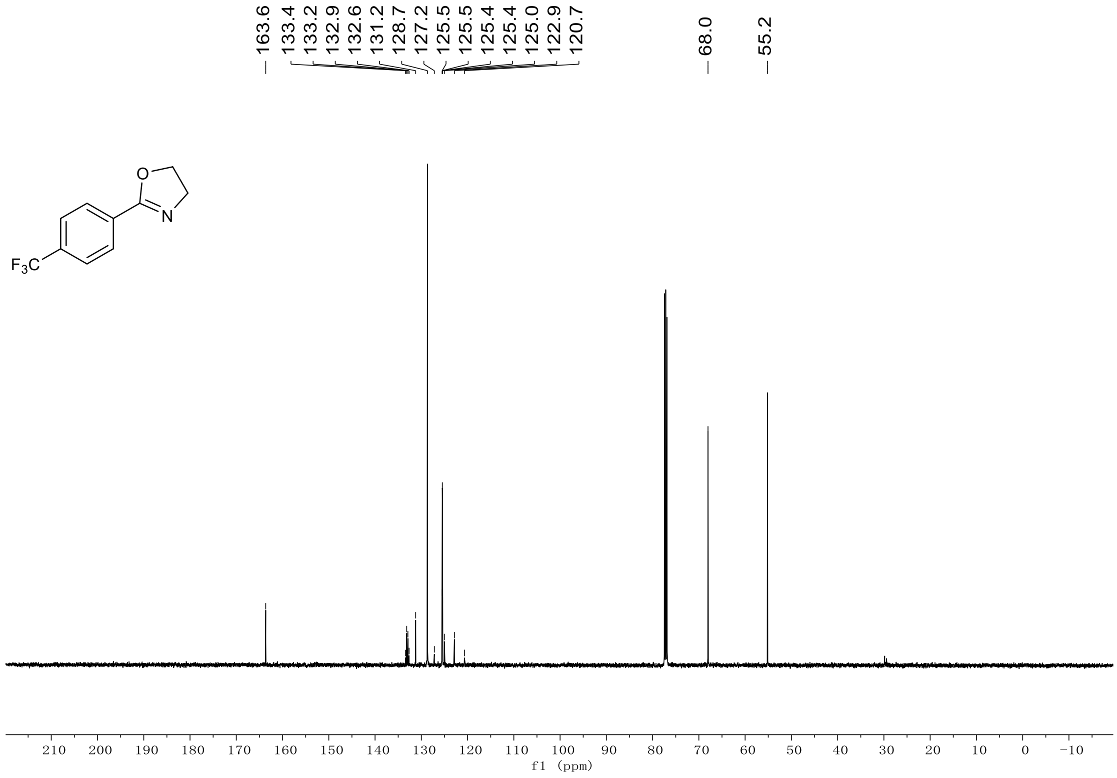

9, ^{19}F NMR (471 MHz, $CDCl_3$)

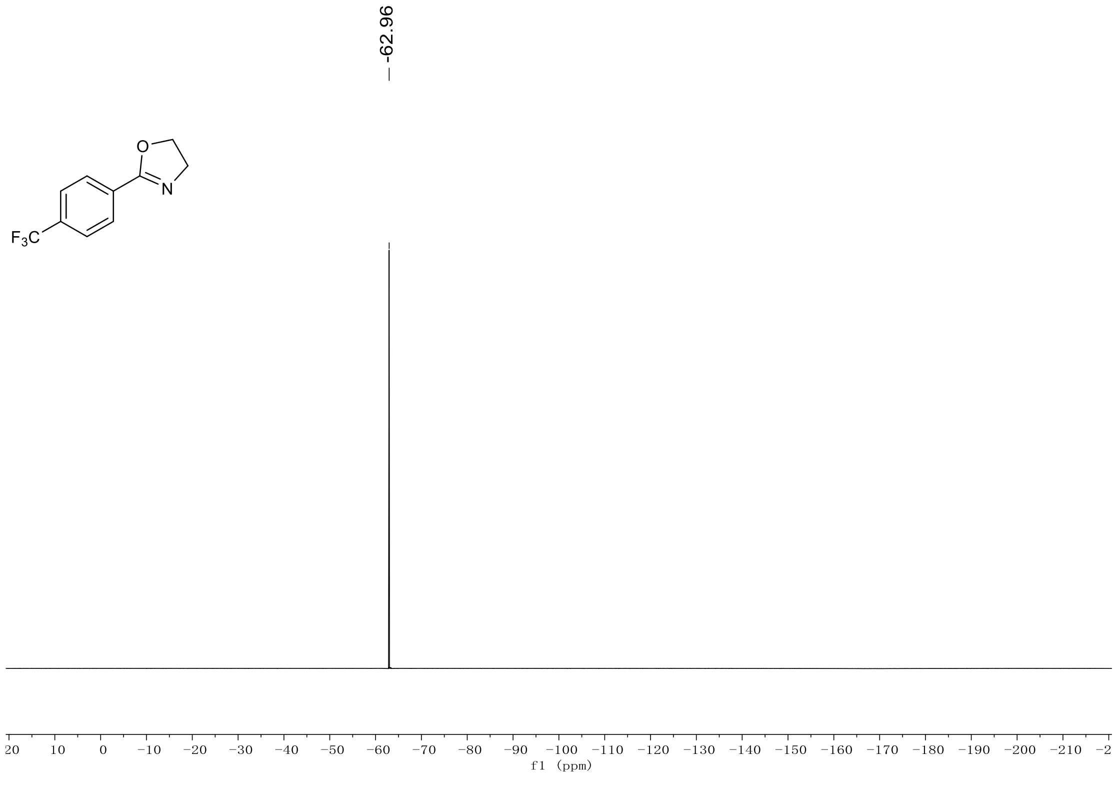

10, ^{1}H NMR (500 MHz, $CDCl_3$)

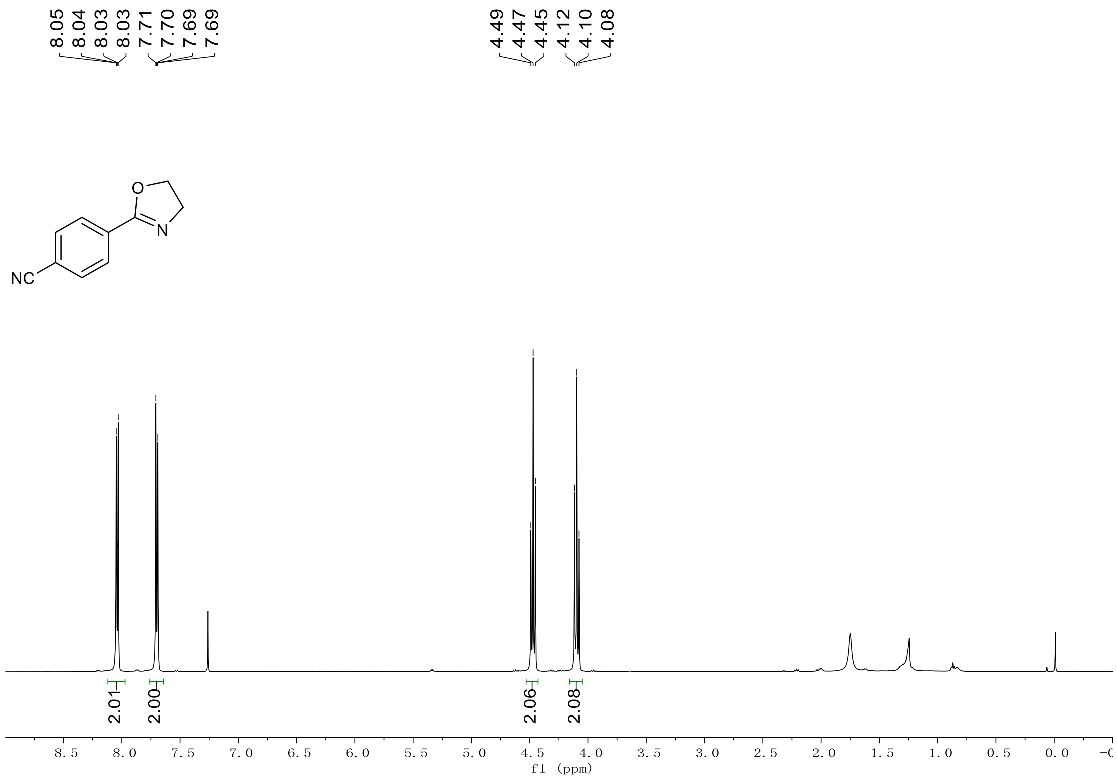

10, ^{13}C NMR (126 MHz, $CDCl_3$)

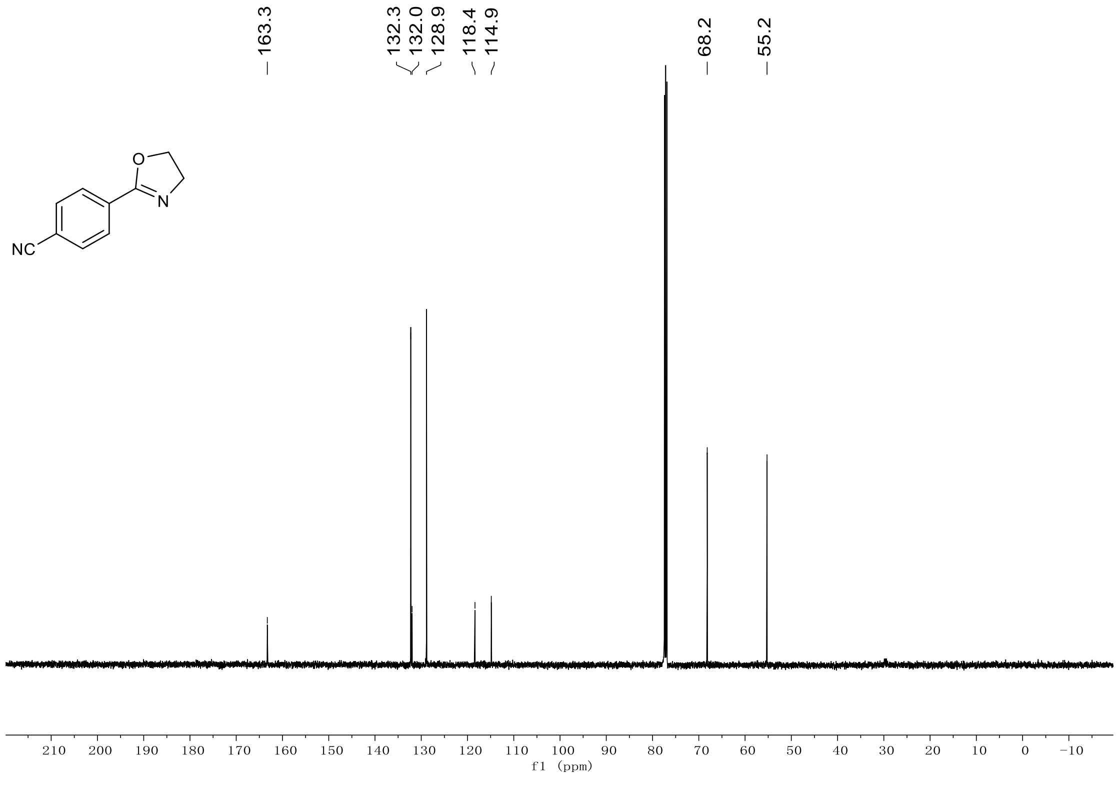

11, ^{1}H NMR (500 MHz, $CDCl_3$)

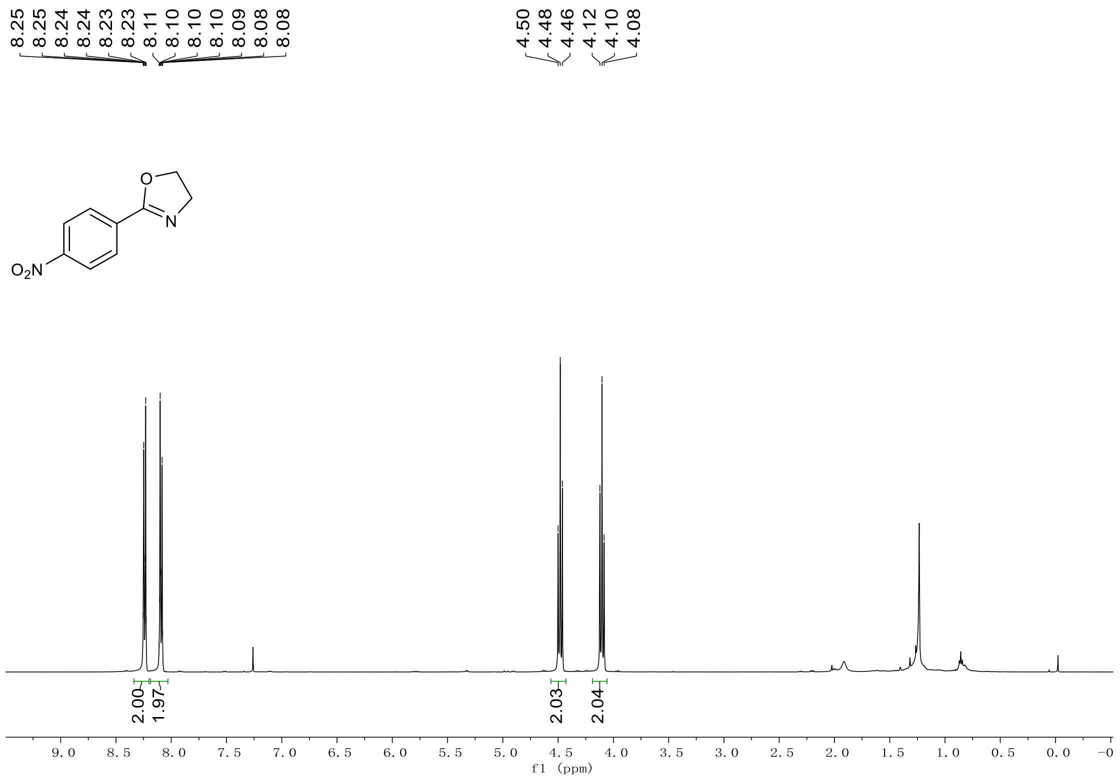

11, ^{13}C NMR (126 MHz, $CDCl_3$)

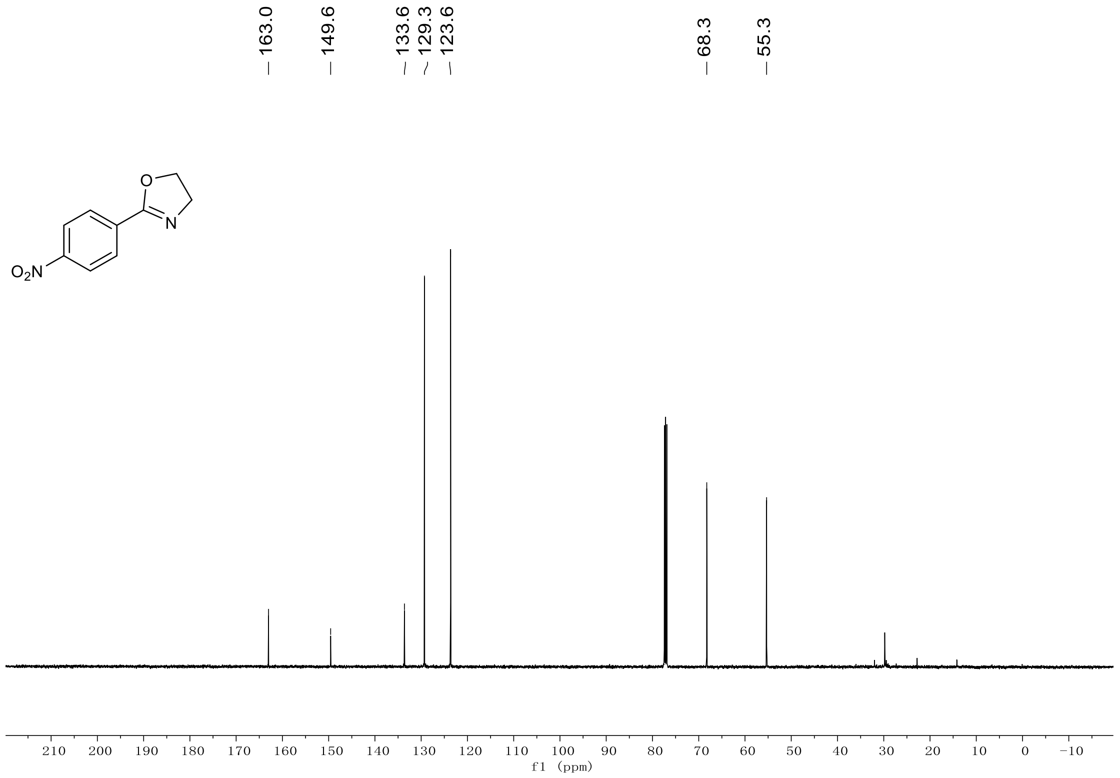

12, ^{1}H NMR (500 MHz, $CDCl_3$)

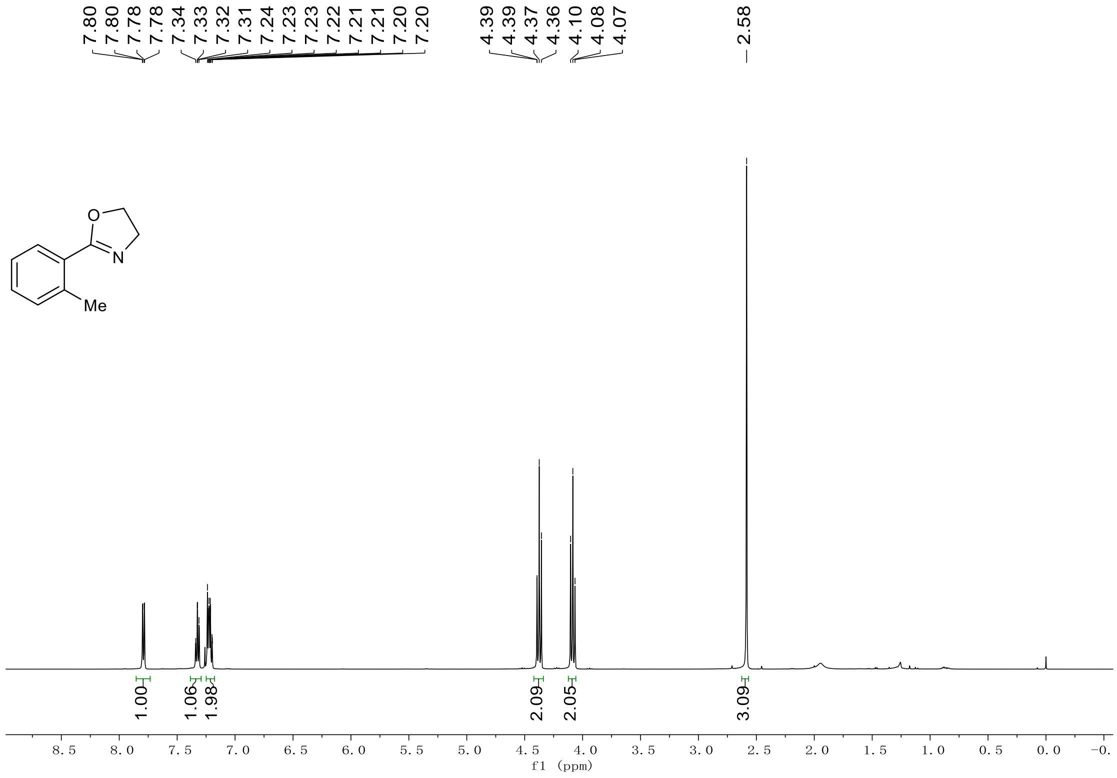

12, ^{13}C NMR (126 MHz, $CDCl_3$)

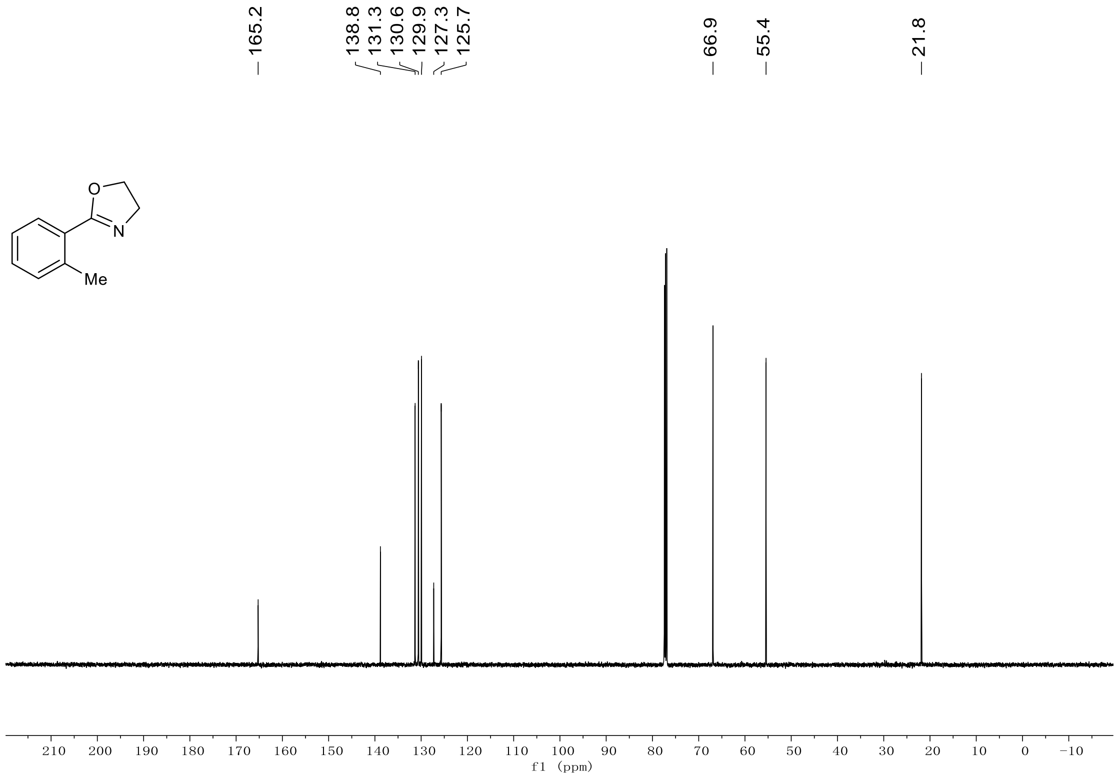

13, ^{1}H NMR (500 MHz, $CDCl_3$)

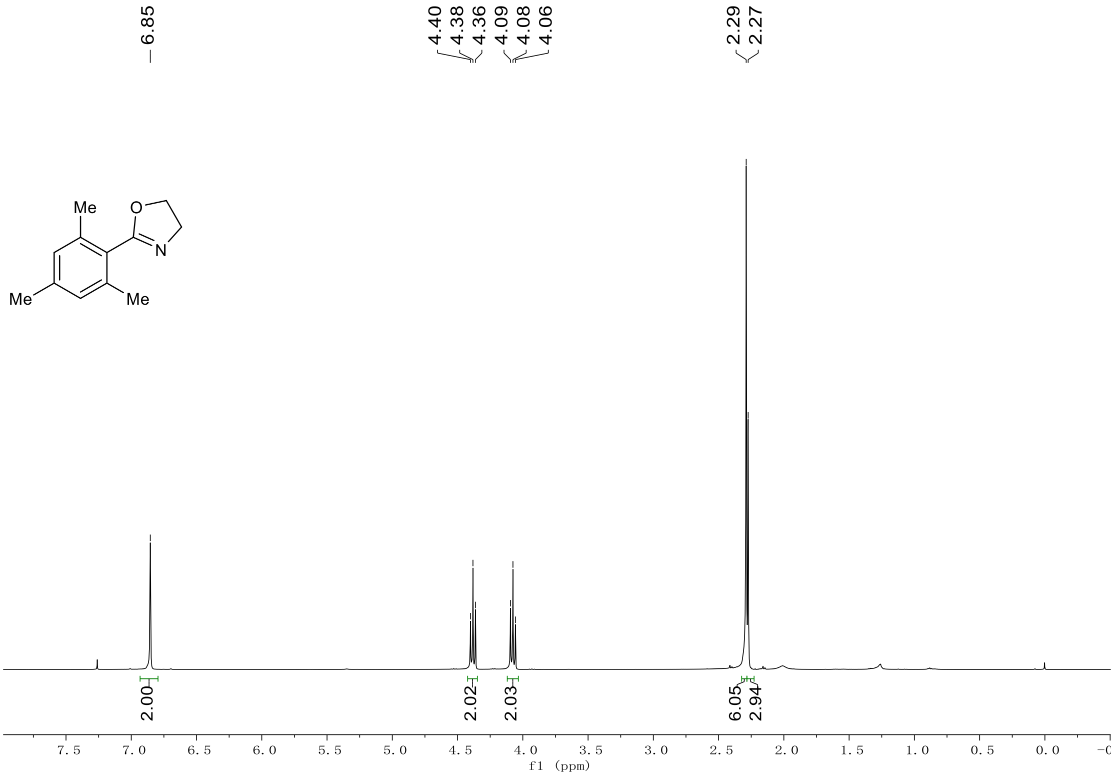

13, ^{13}C NMR (126 MHz, $CDCl_3$)

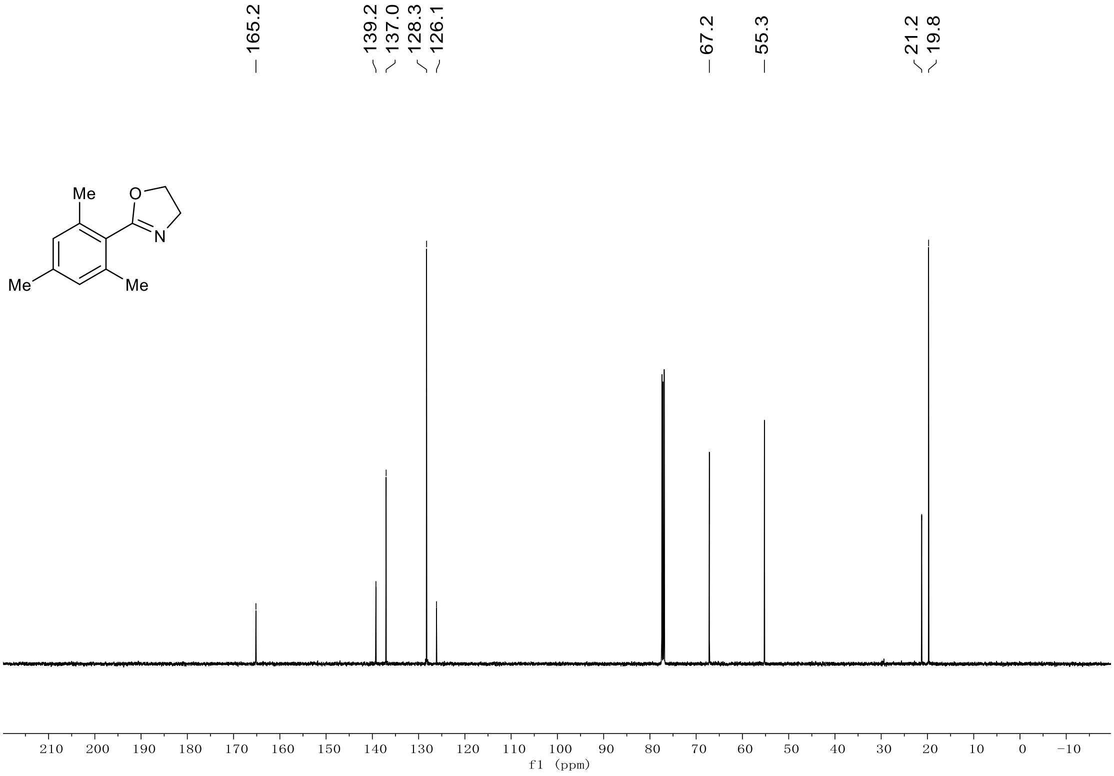

14, ^{1}H NMR (500 MHz, $CDCl_3$)

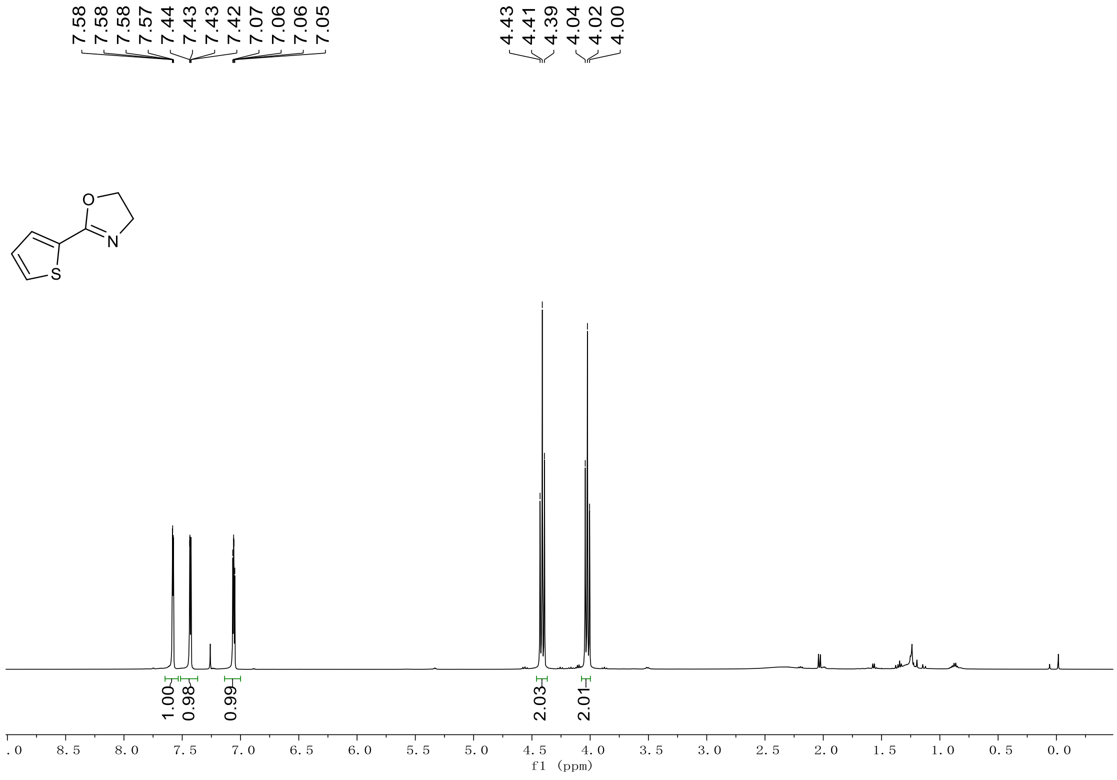

14, ^{13}C NMR (126 MHz, $CDCl_3$)

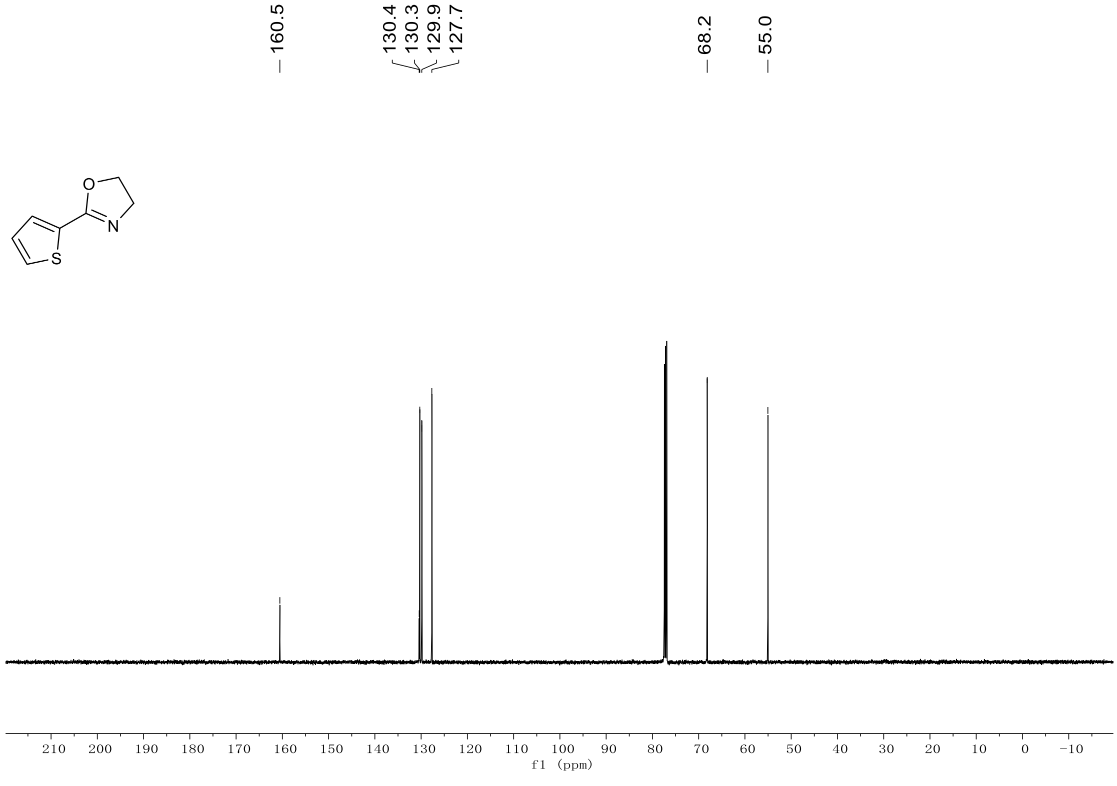

15, ^{1}H NMR (500 MHz, $CDCl_3$)

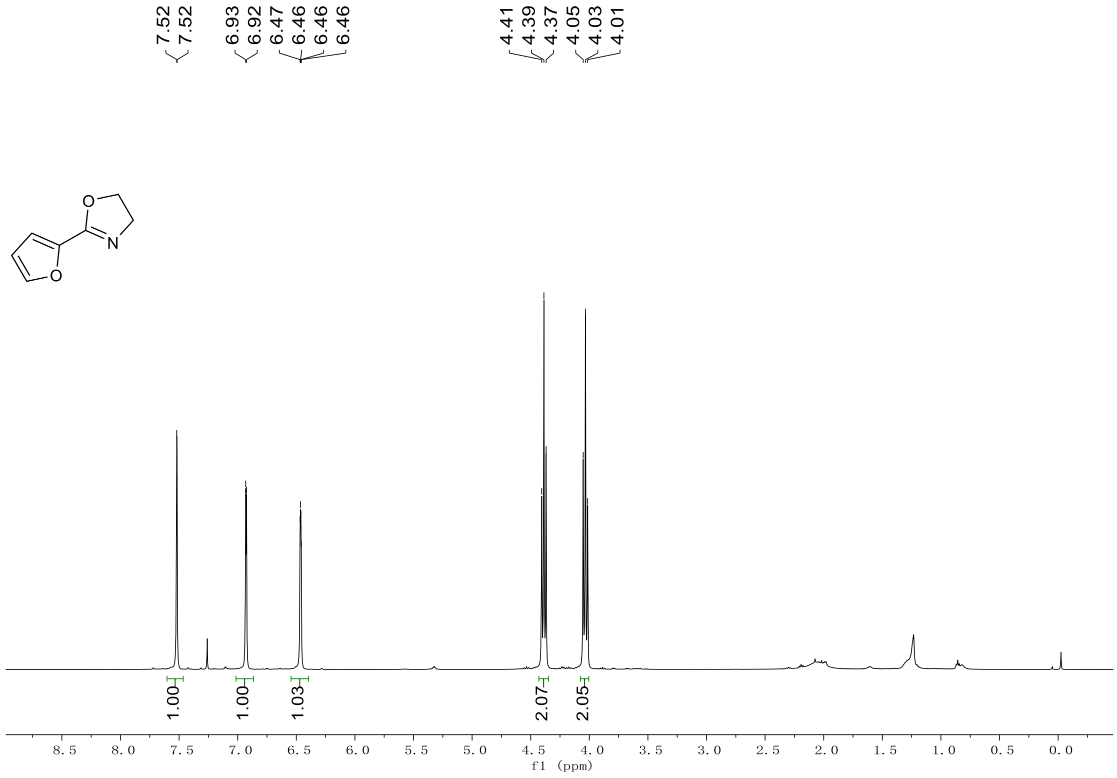

15, ^{13}C NMR (126 MHz, $CDCl_3$)

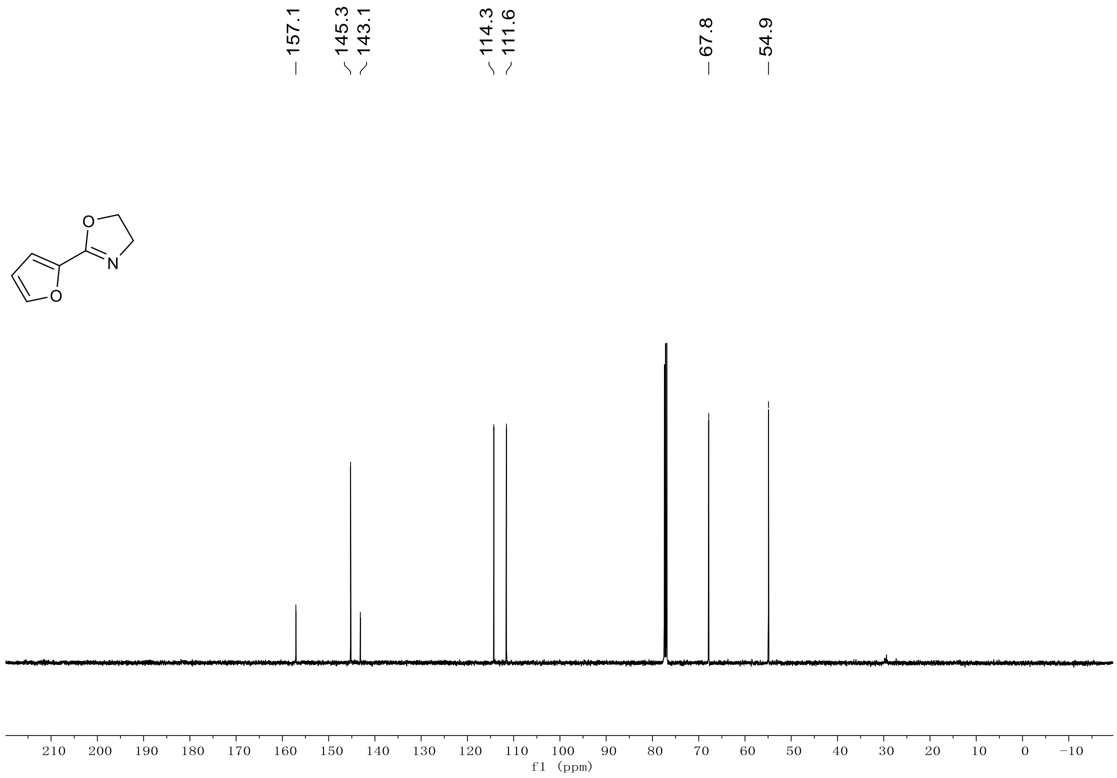

16, ^{1}H NMR (500 MHz, $CDCl_3$)

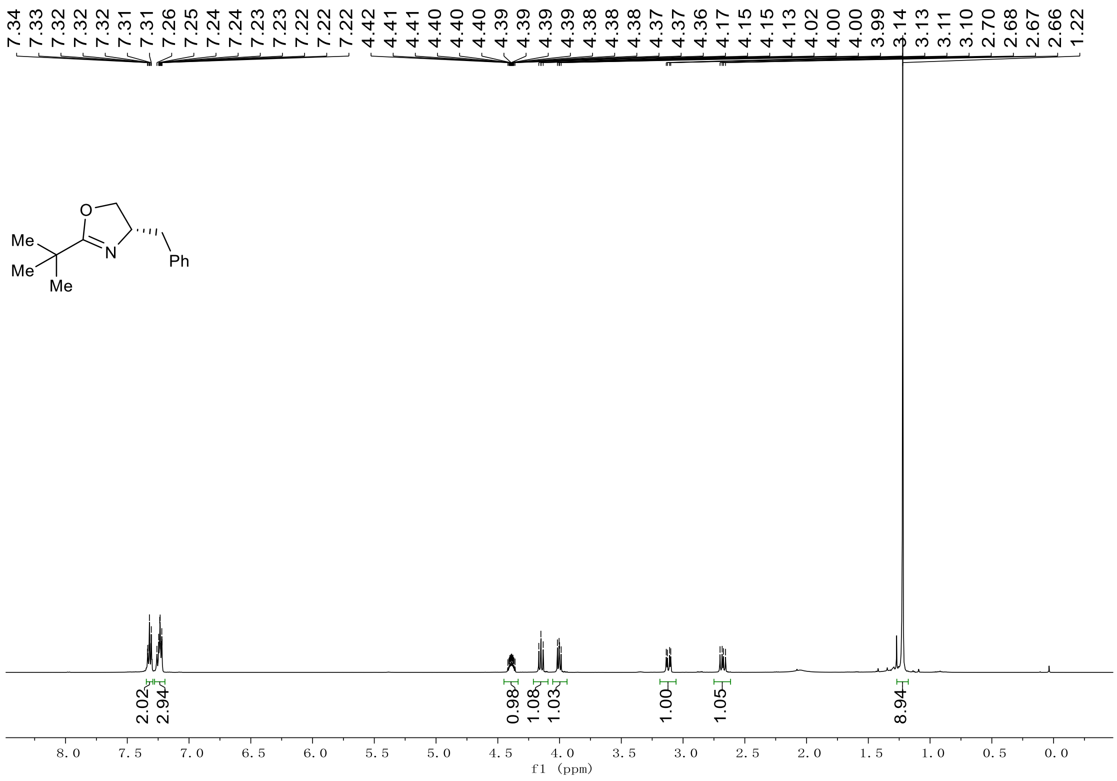

16, ^{13}C NMR (126 MHz, $CDCl_3$)

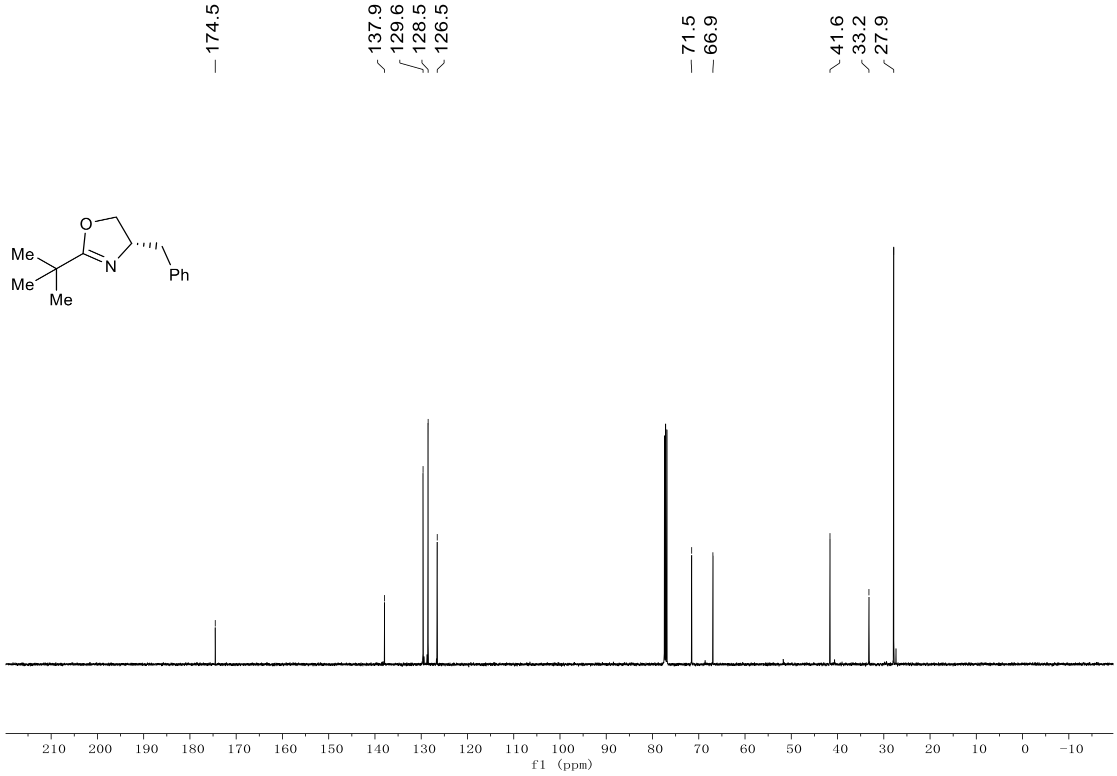

17, ^{1}H NMR (500 MHz, $CDCl_3$)

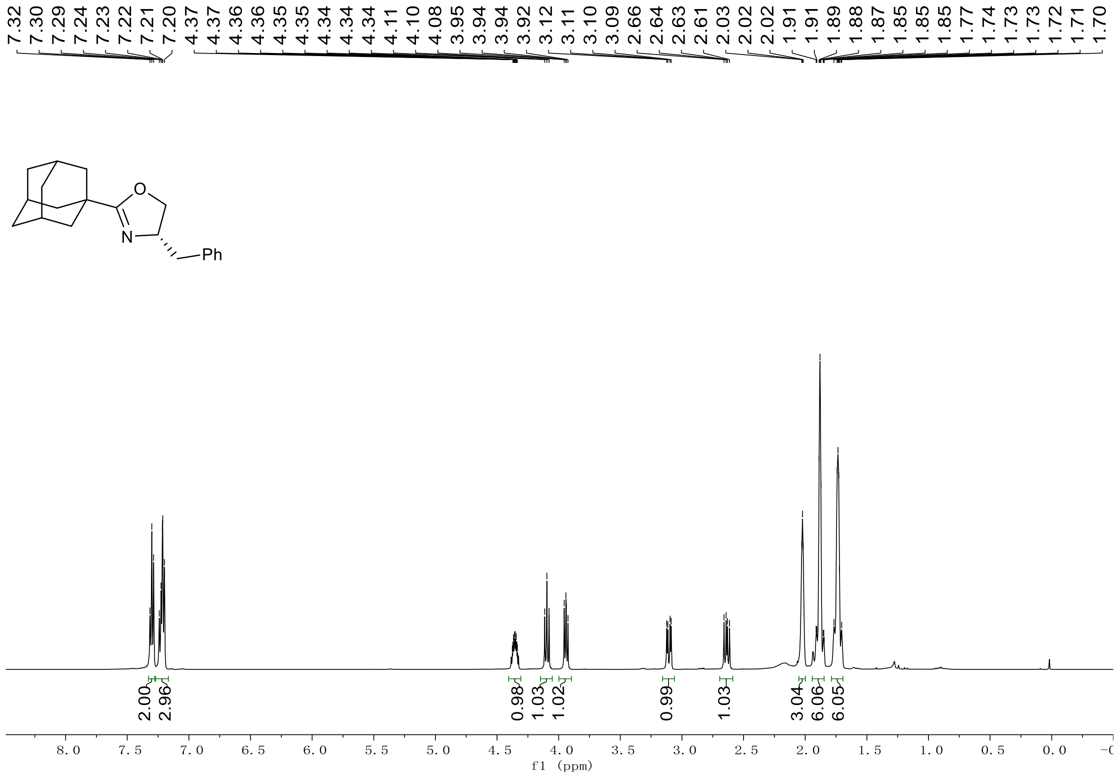

17, ^{13}C NMR (126 MHz, $CDCl_3$)

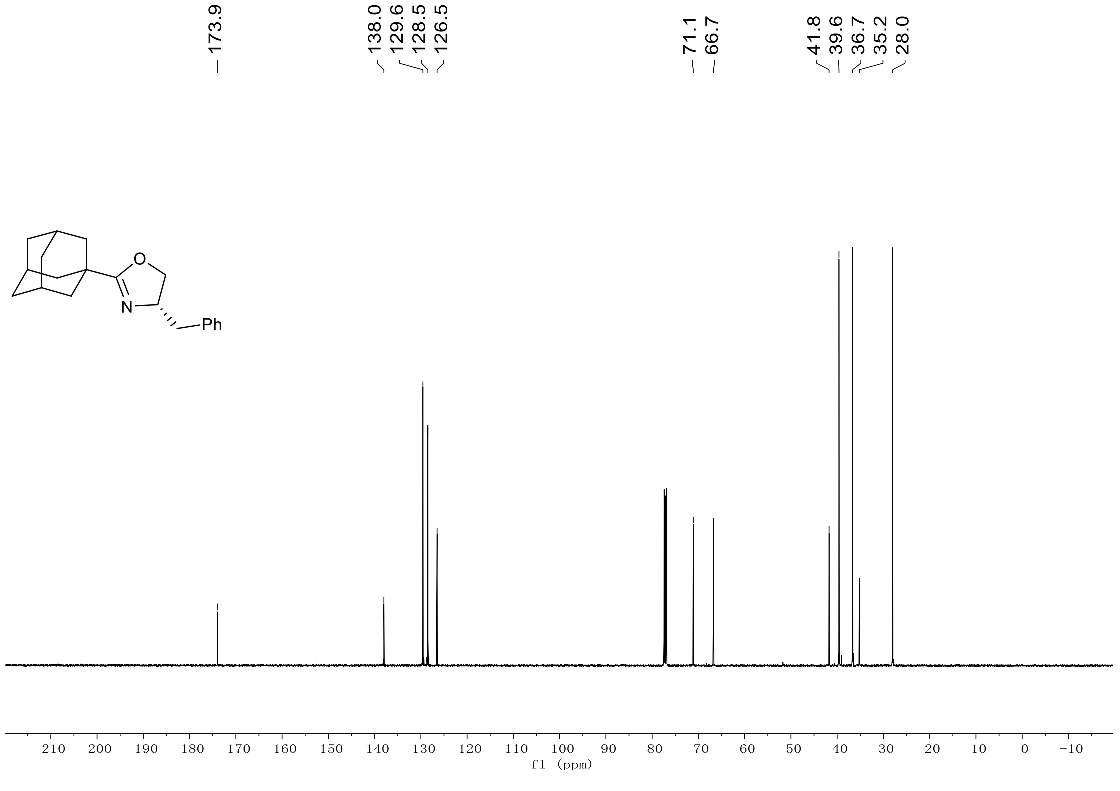

18, ^{1}H NMR (500 MHz, $CDCl_3$)

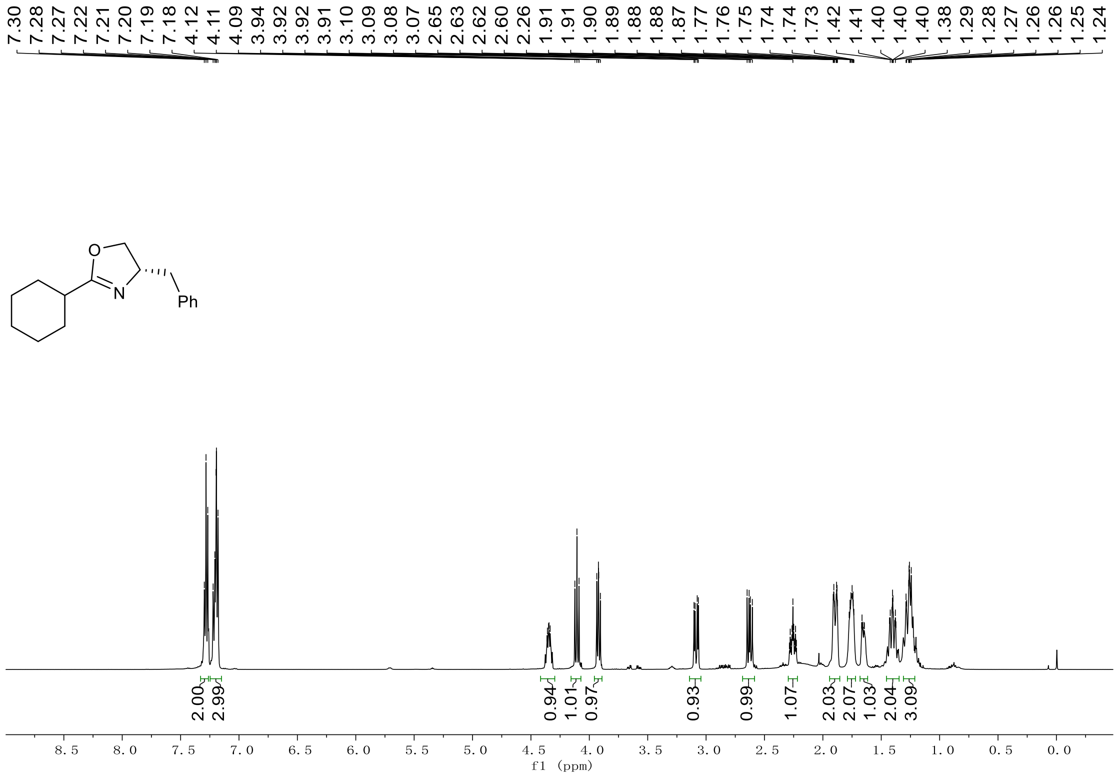

18, ^{13}C NMR (126 MHz, $CDCl_3$)

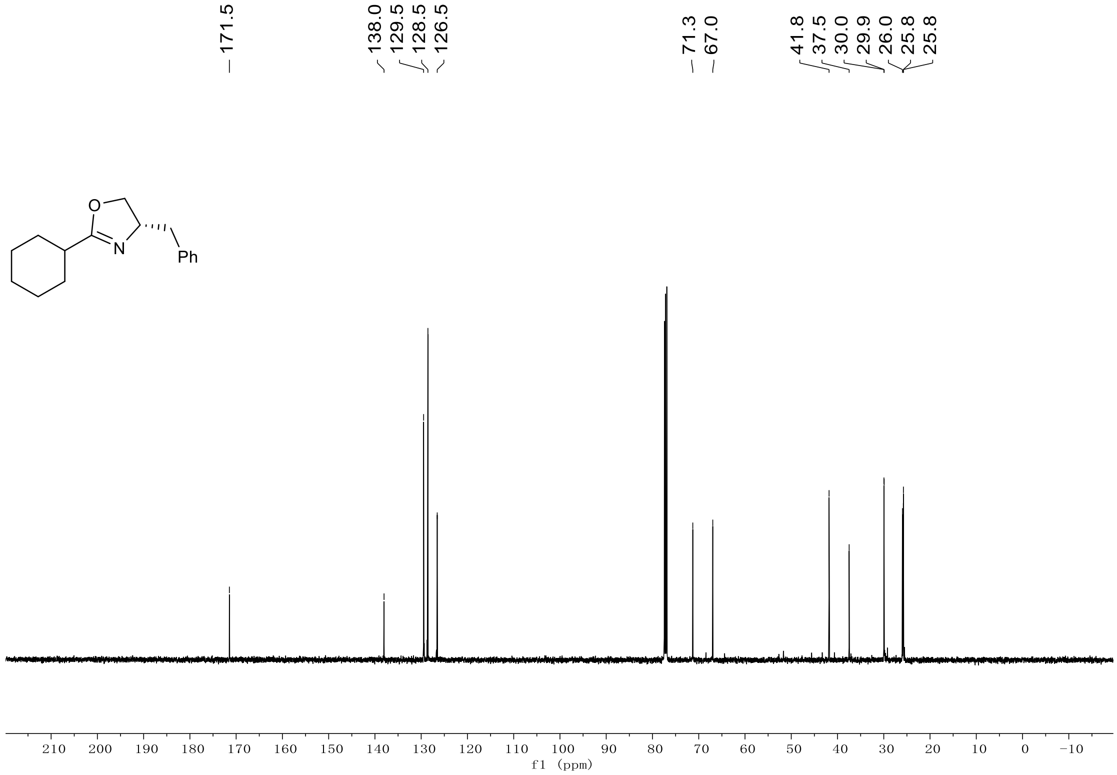

19, ^{1}H NMR (500 MHz, CDCl$_3$)

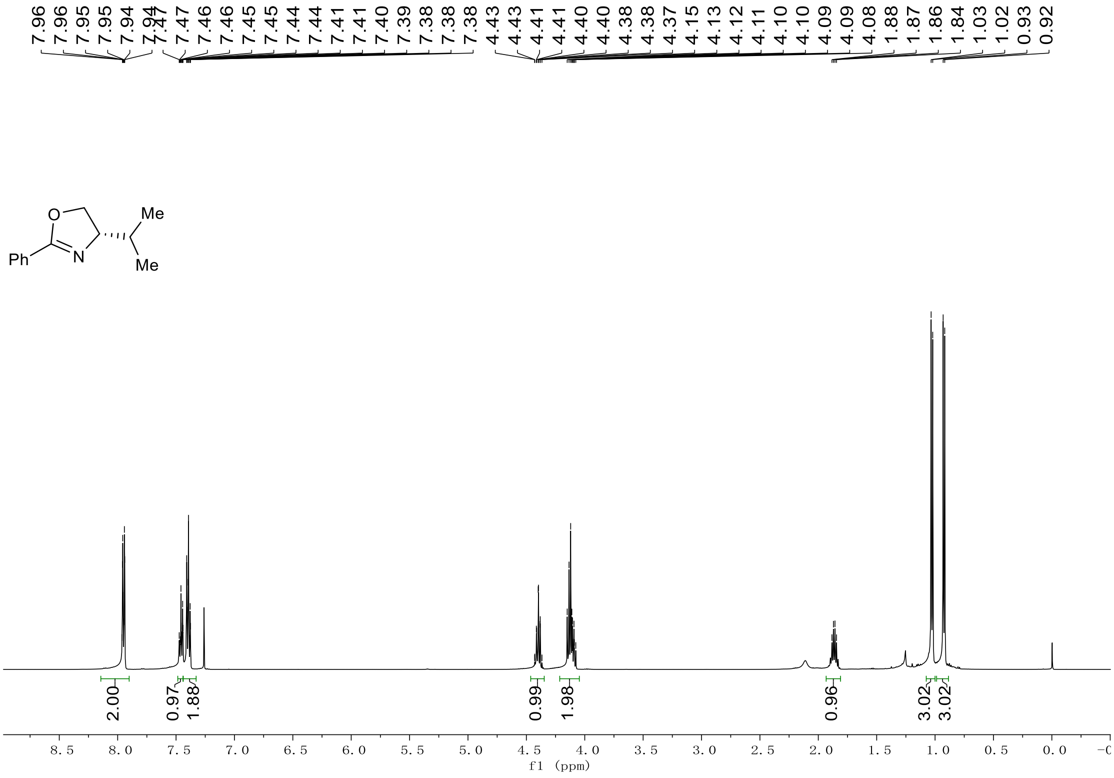

19, ^{13}C NMR (126 MHz, CDCl$_3$)

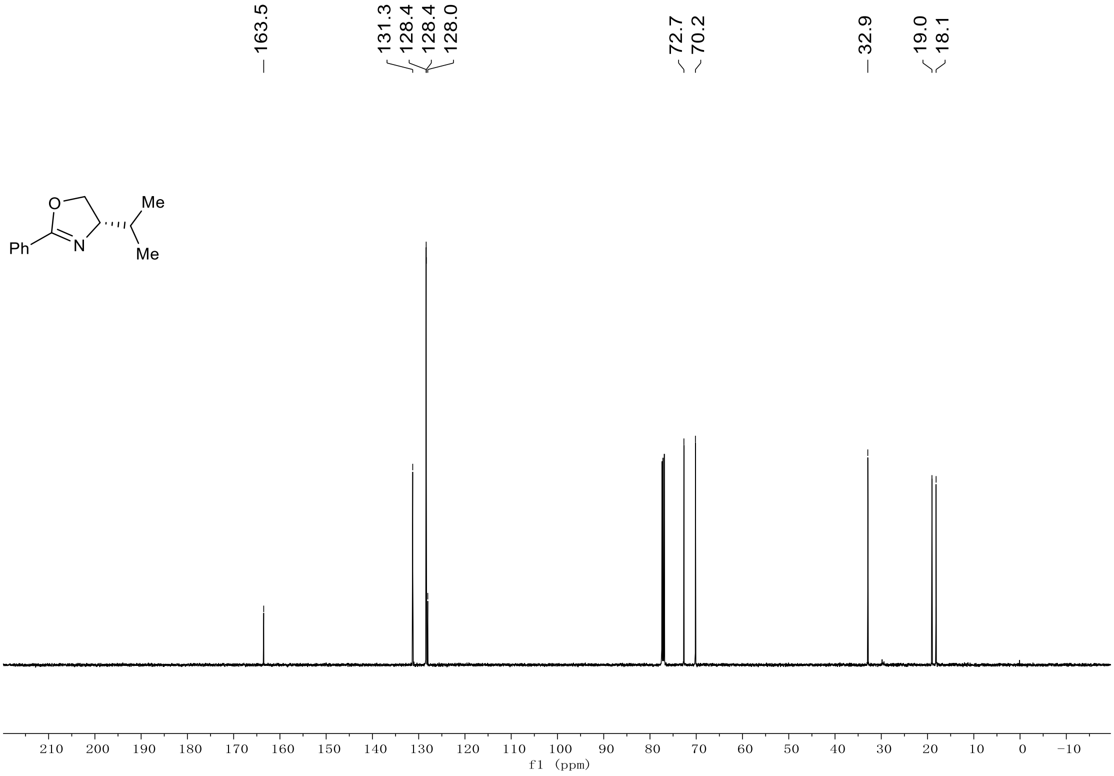

20, ^{1}H NMR (500 MHz, $CDCl_3$)

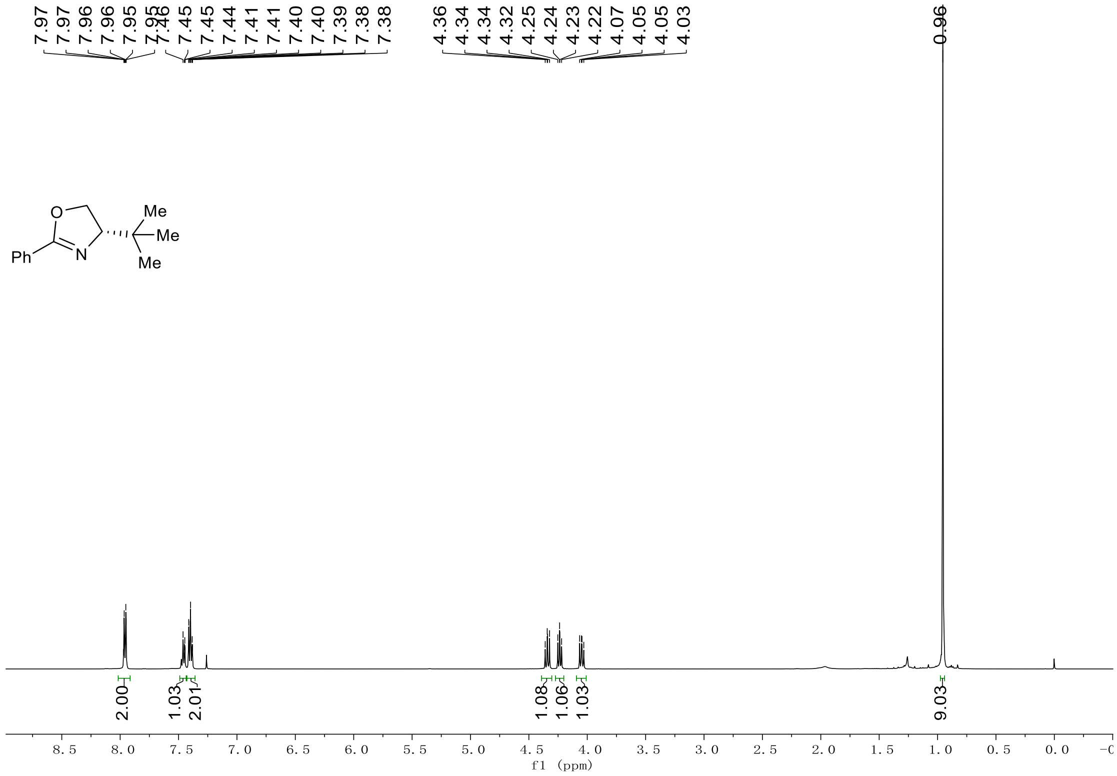

20, ^{13}C NMR (126 MHz, $CDCl_3$)

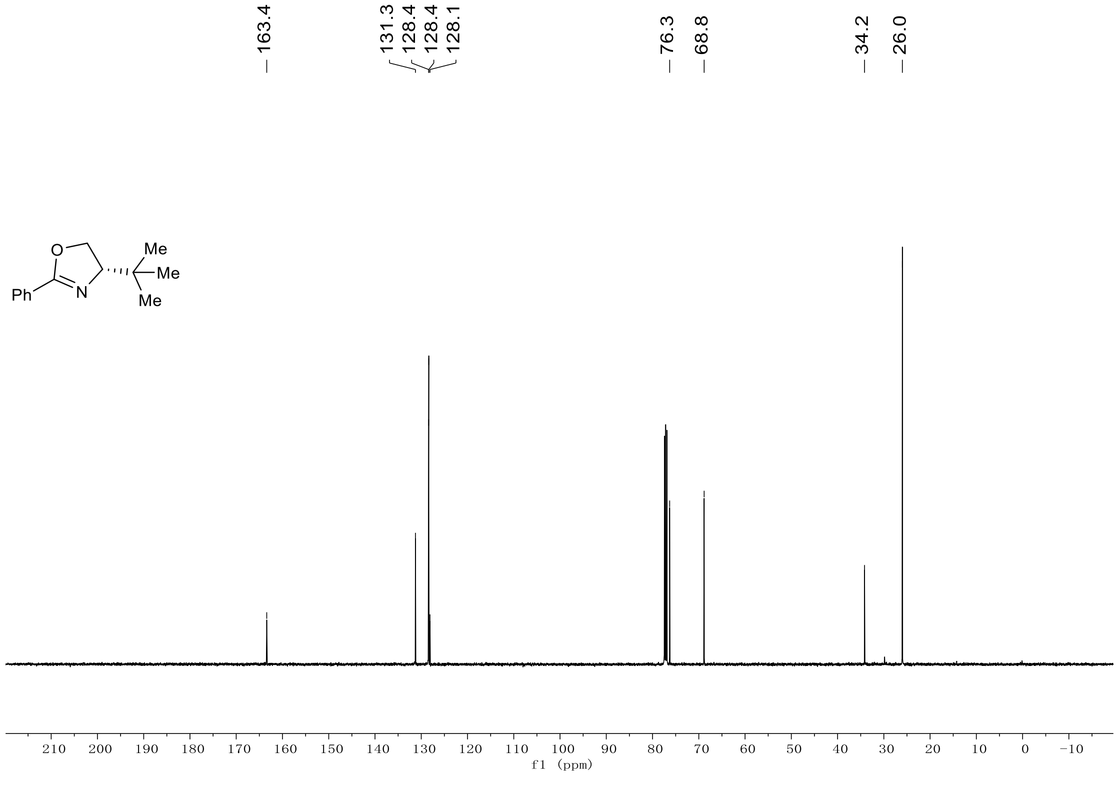

21, ^{1}H NMR (500 MHz, CDCl$_3$)

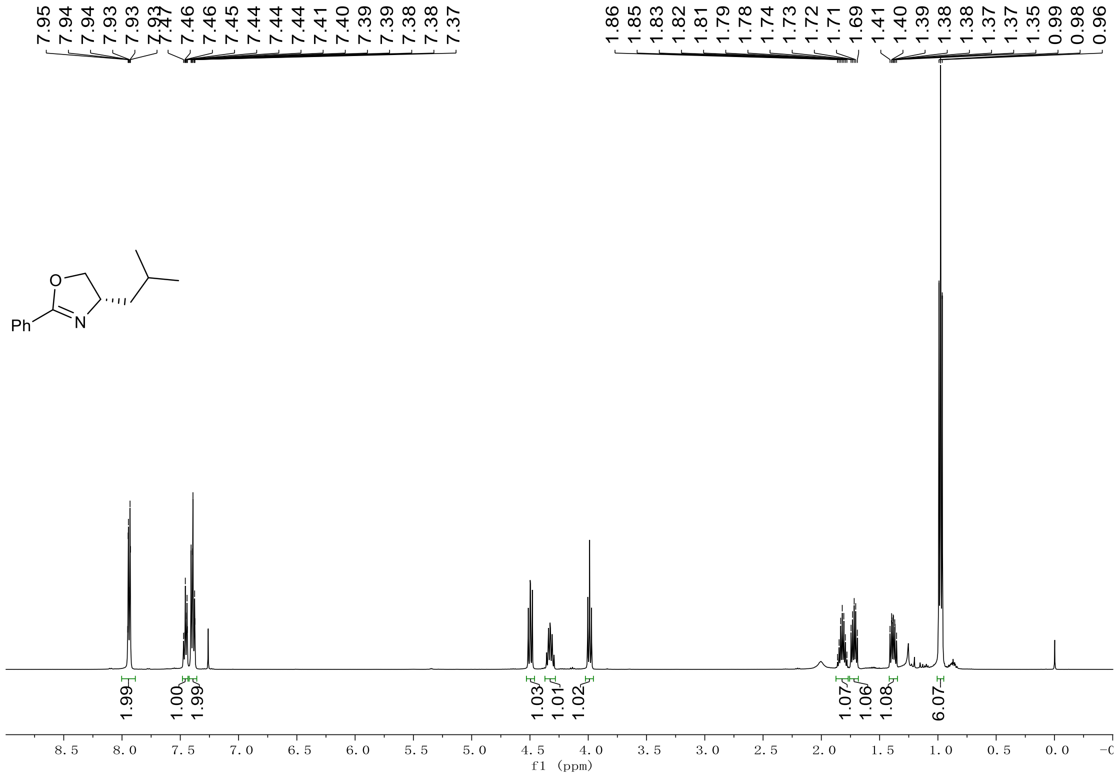

21, ^{13}C NMR (126 MHz, CDCl$_3$)

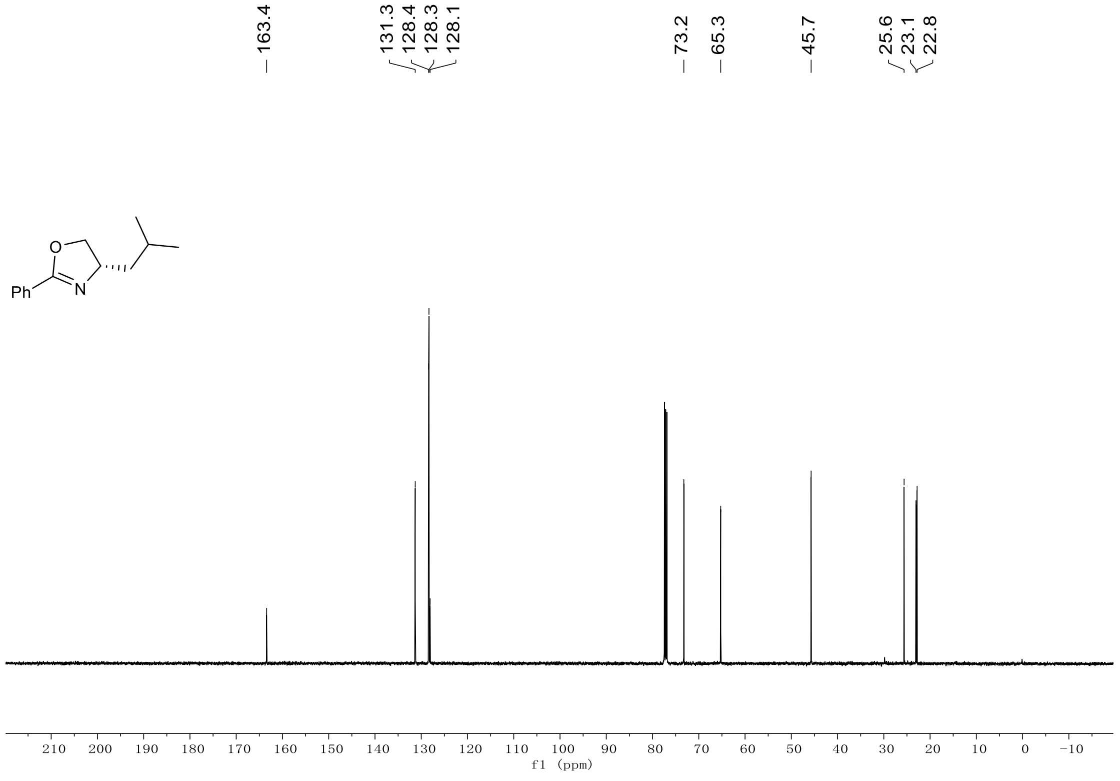

22, ^{1}H NMR (500 MHz, $CDCl_3$)

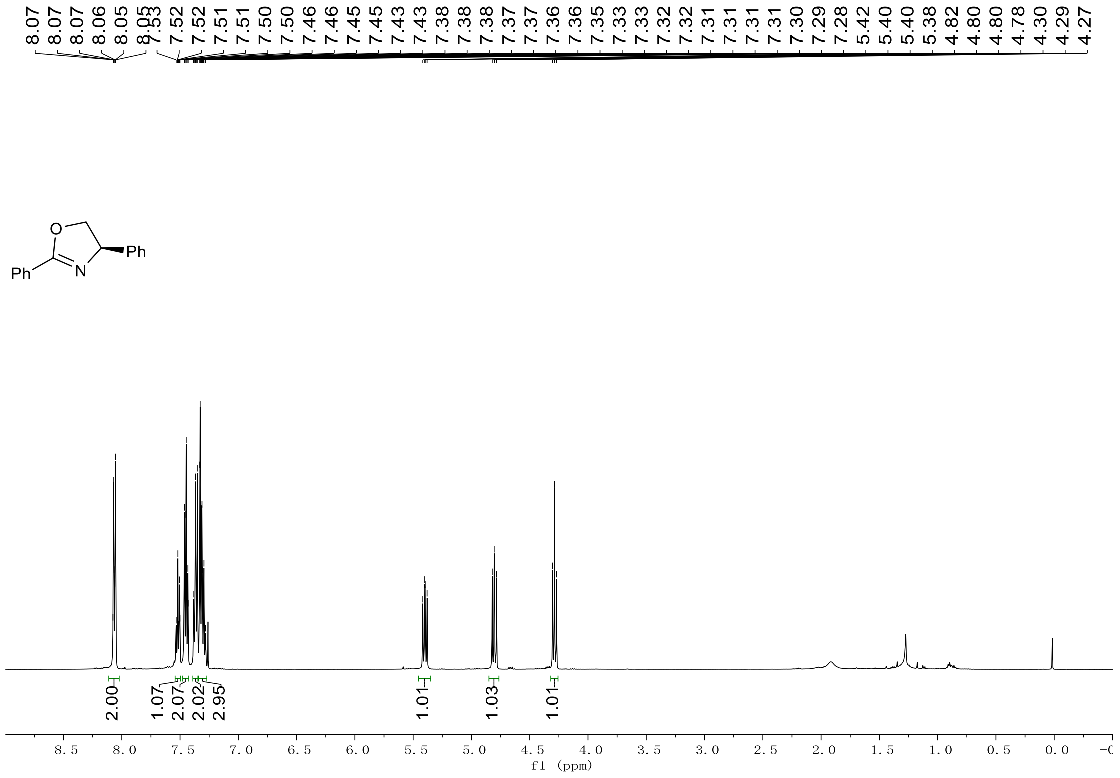

22, ^{13}C NMR (126 MHz, $CDCl_3$)

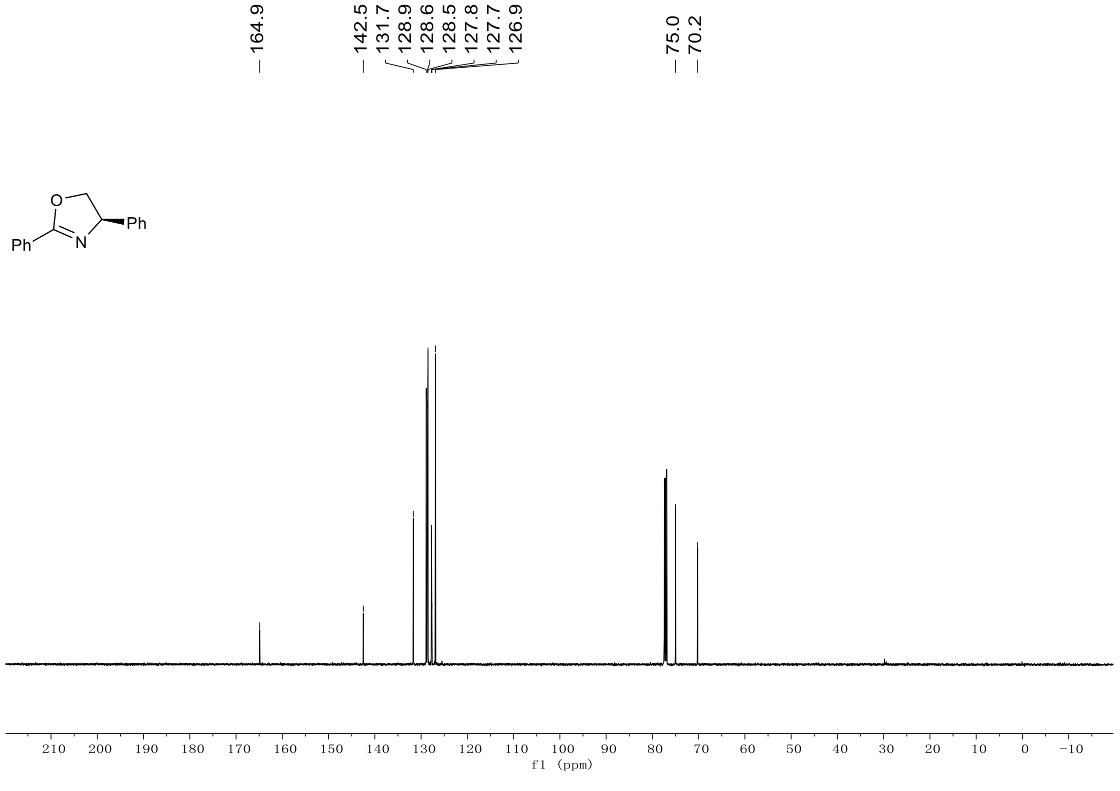

23, ^{1}H NMR (500 MHz, $CDCl_3$)

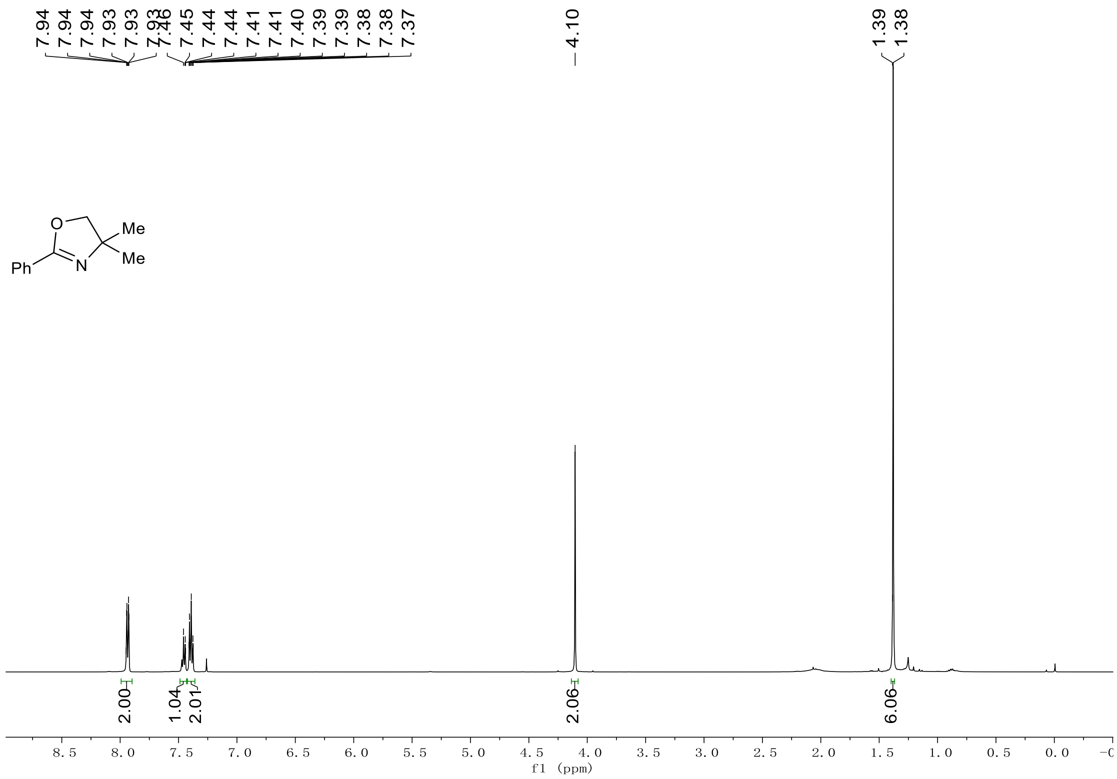

23, ^{13}C NMR (126 MHz, $CDCl_3$)

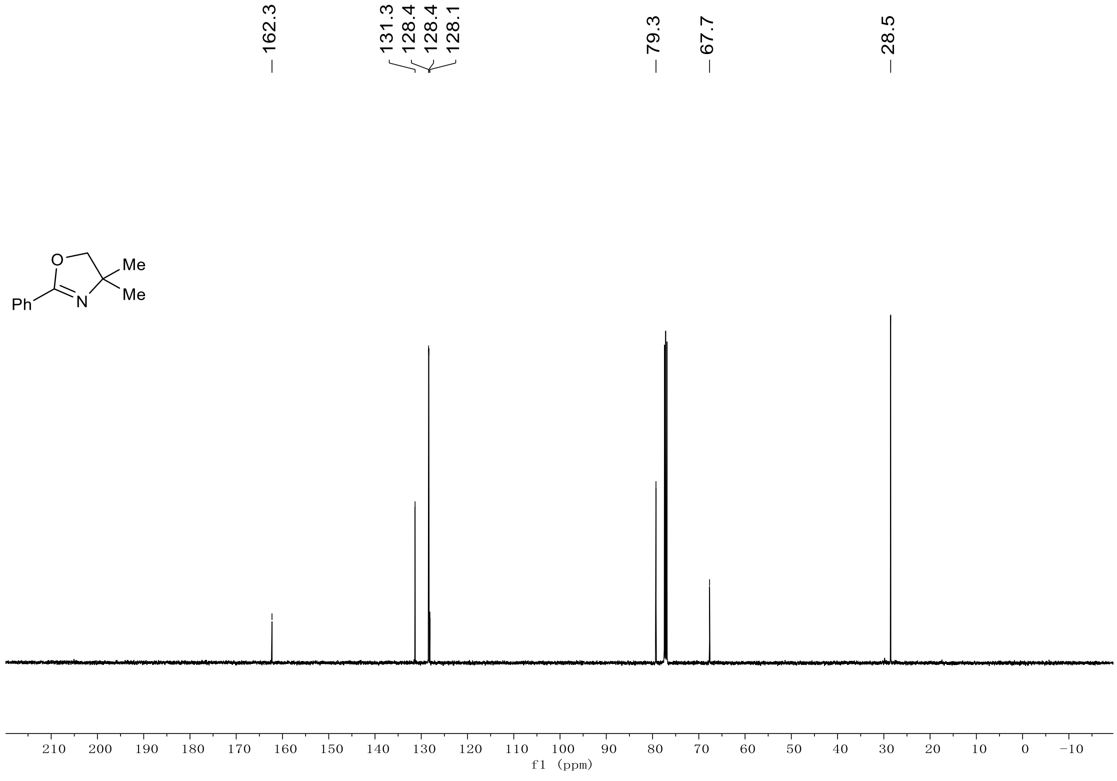

24, ^{1}H NMR (500 MHz, $CDCl_3$)

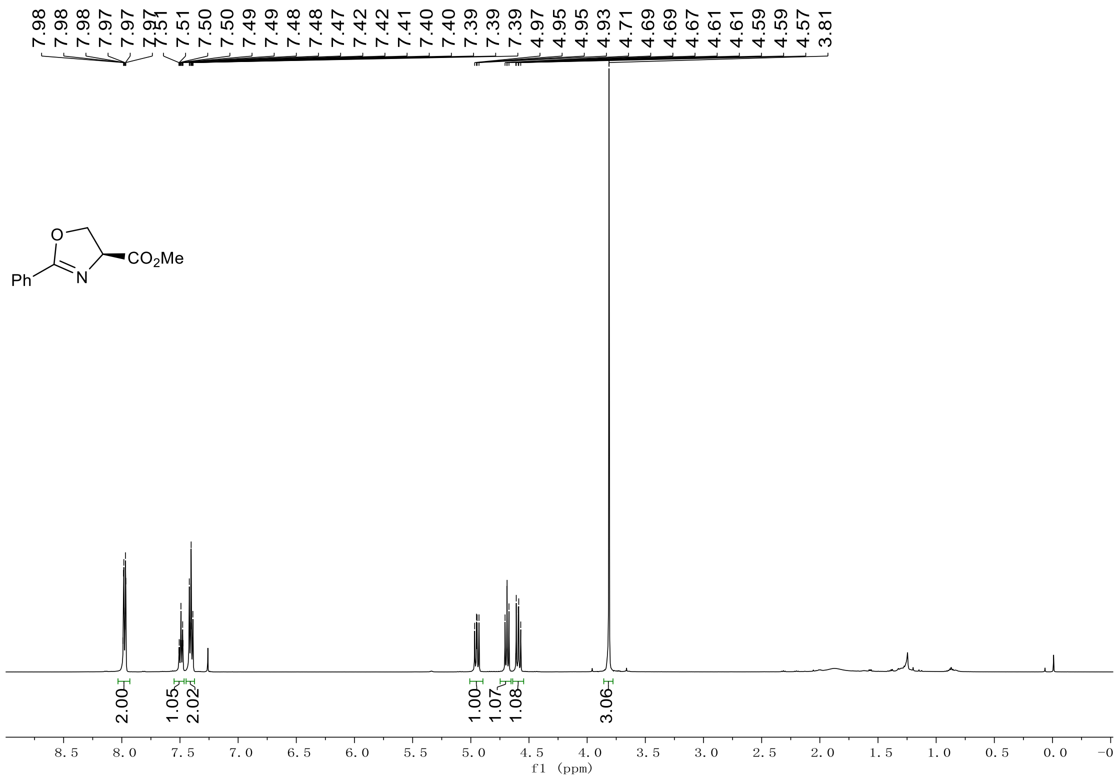

24 ^{13}C NMR (126 MHz, $CDCl_3$)

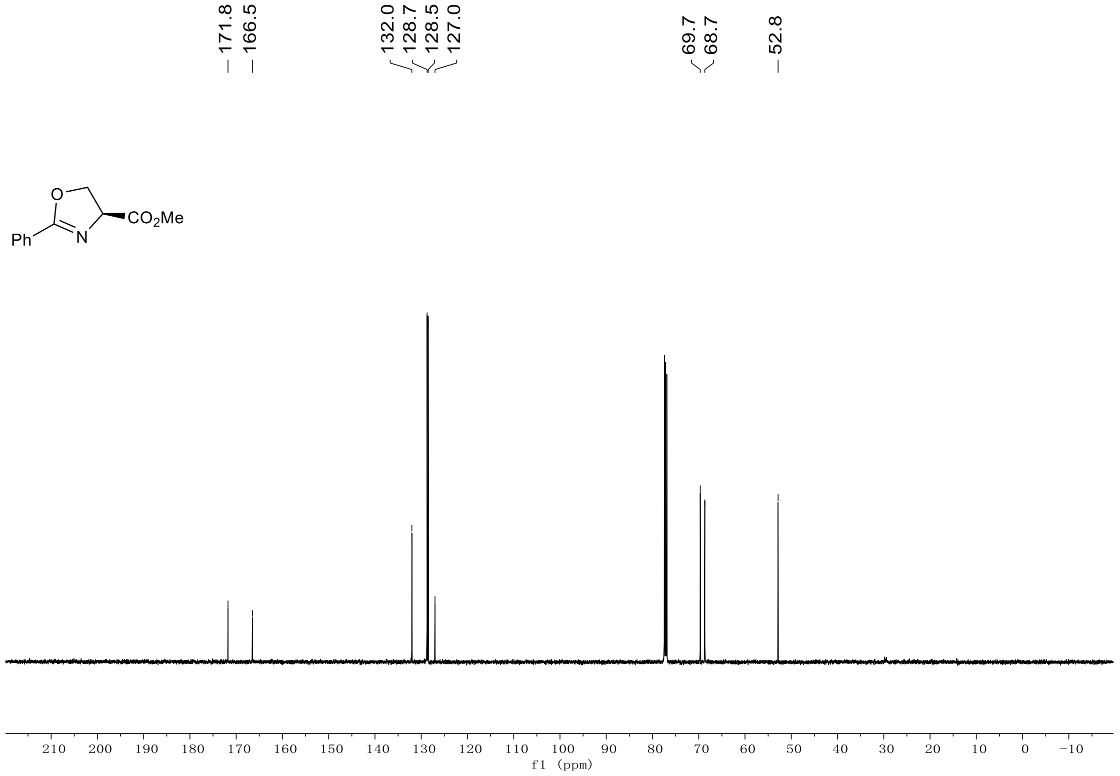

25, ^{1}H NMR (500 MHz, $CDCl_3$)

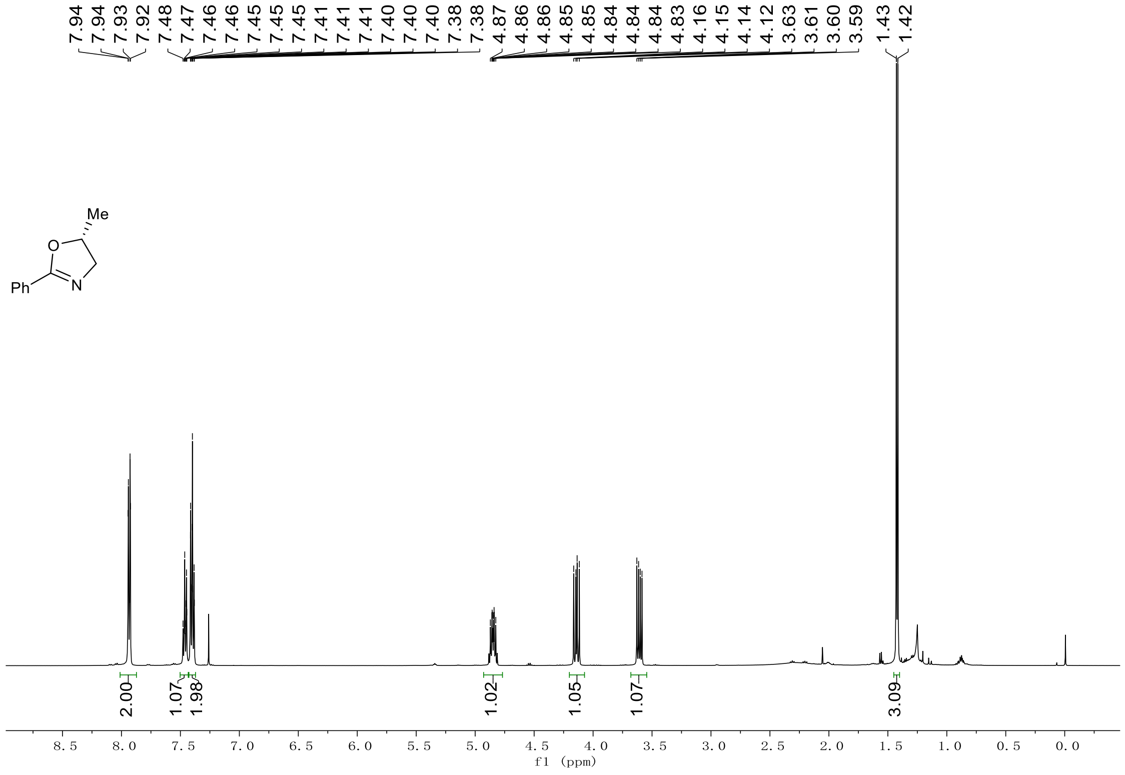

25, ^{13}C NMR (126 MHz, $CDCl_3$)

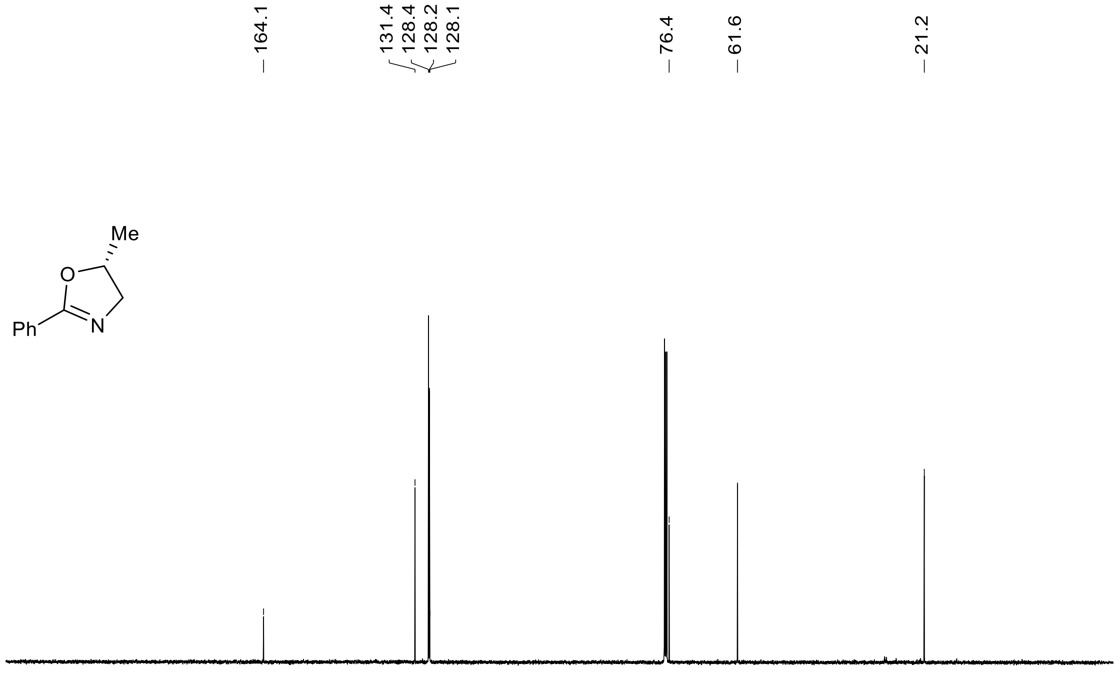

26, ^{1}H NMR (500 MHz, $CDCl_3$)

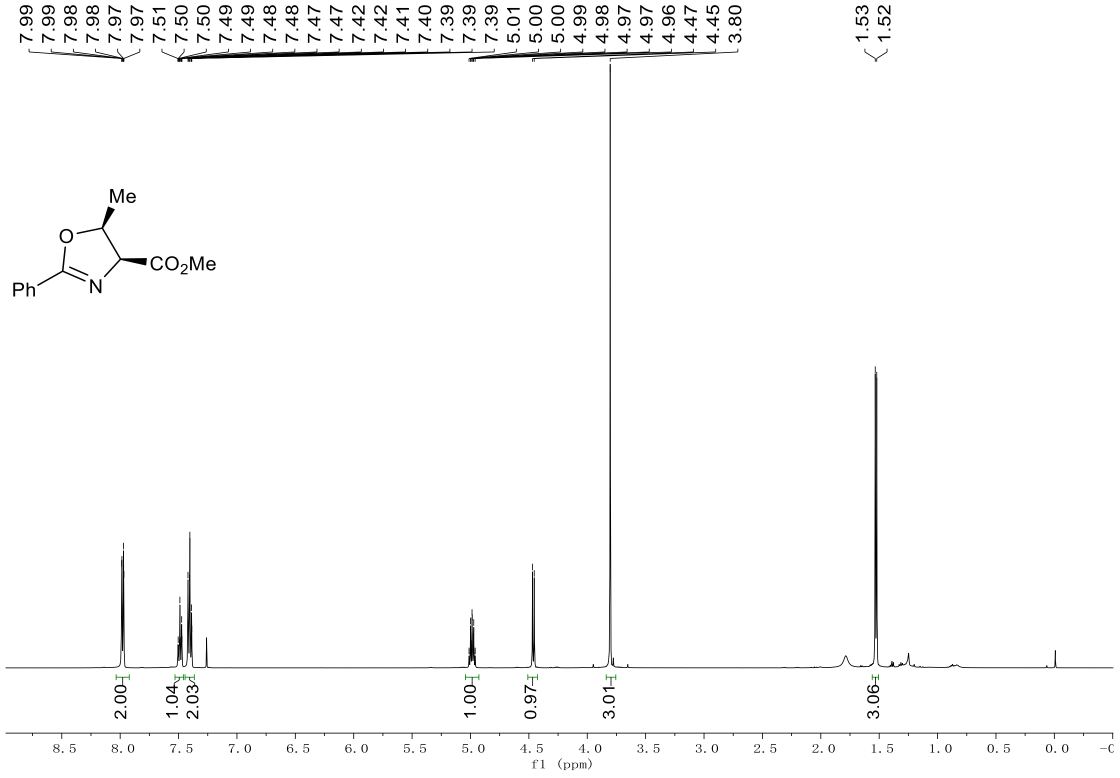

26, ^{13}C NMR (126 MHz, $CDCl_3$)

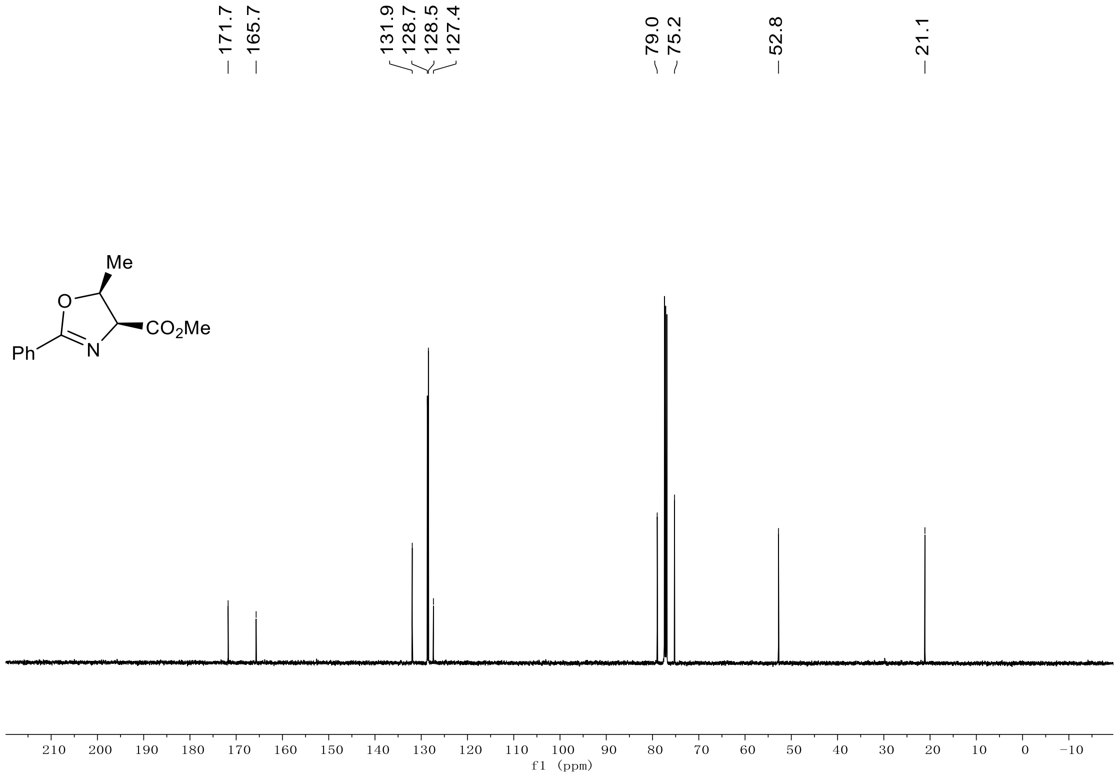

27, ^{1}H NMR (500 MHz, $CDCl_3$)

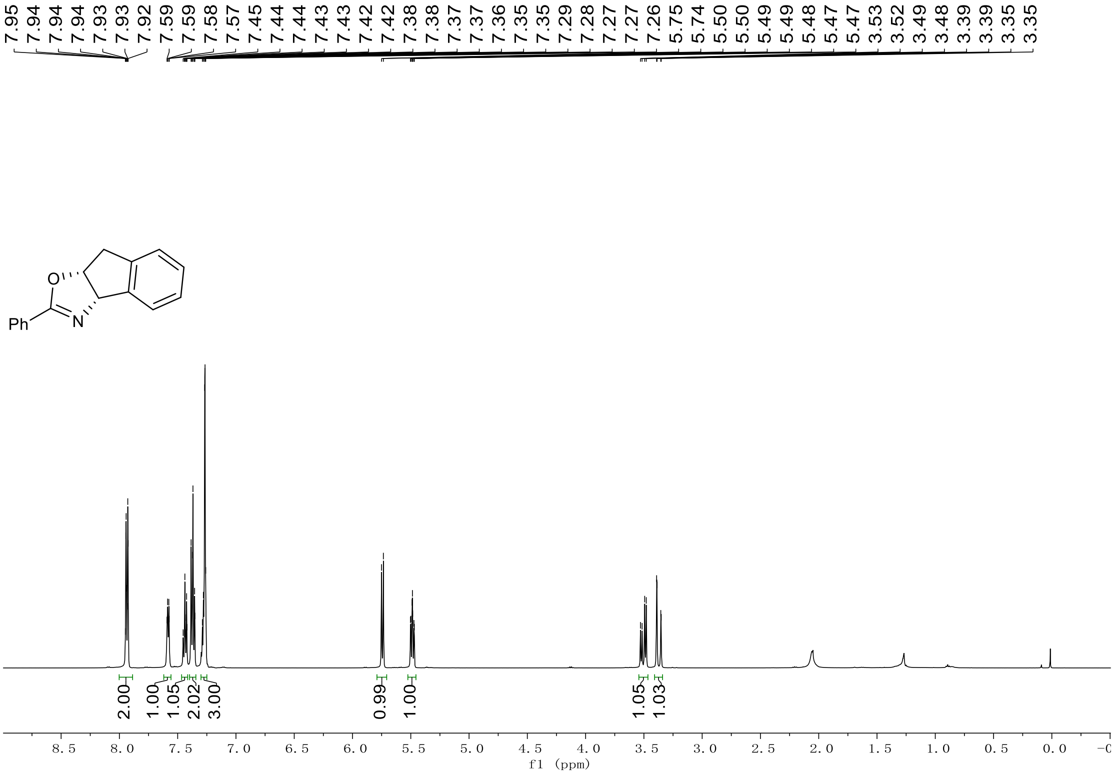

27, ^{13}C NMR (126 MHz, $CDCl_3$)

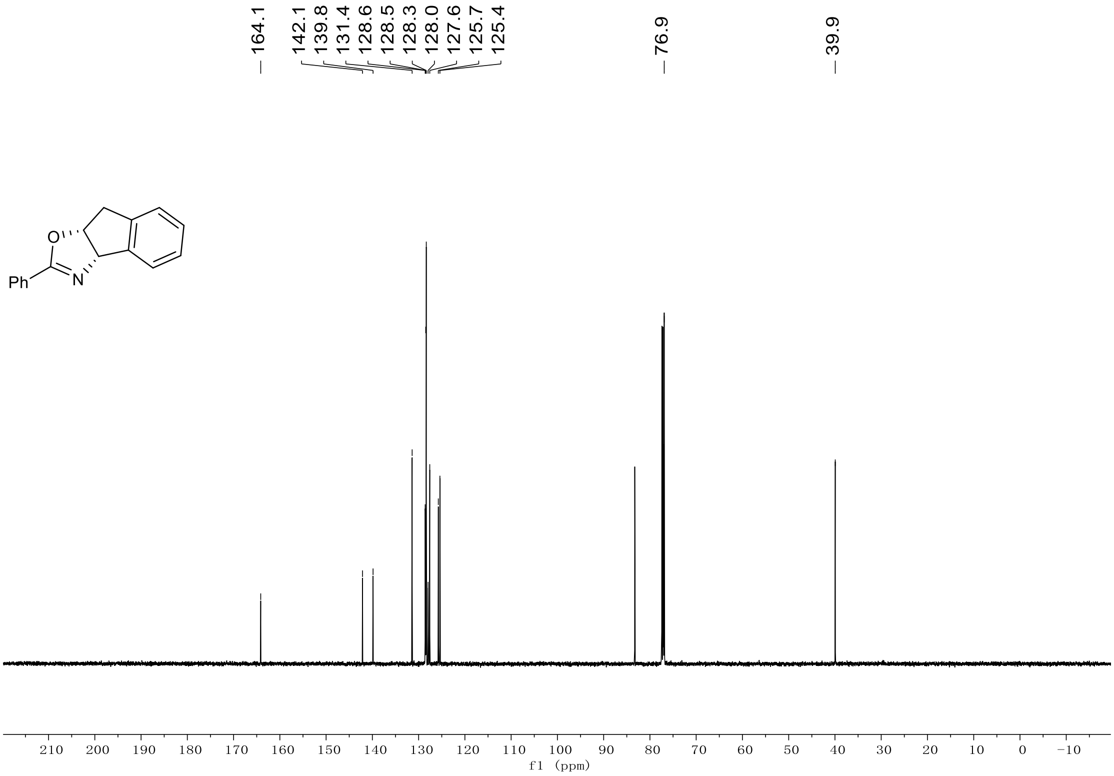

28, ^{1}H NMR (500 MHz, $CDCl_3$)

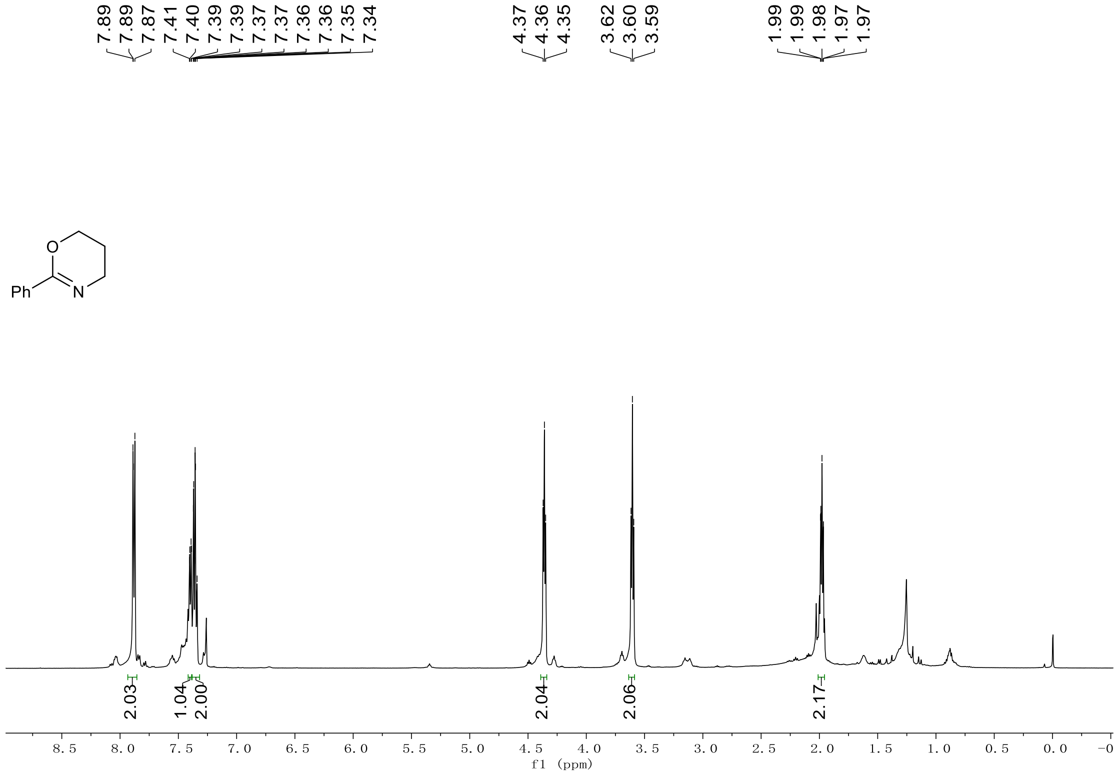

28, ^{13}C NMR (126 MHz, $CDCl_3$)

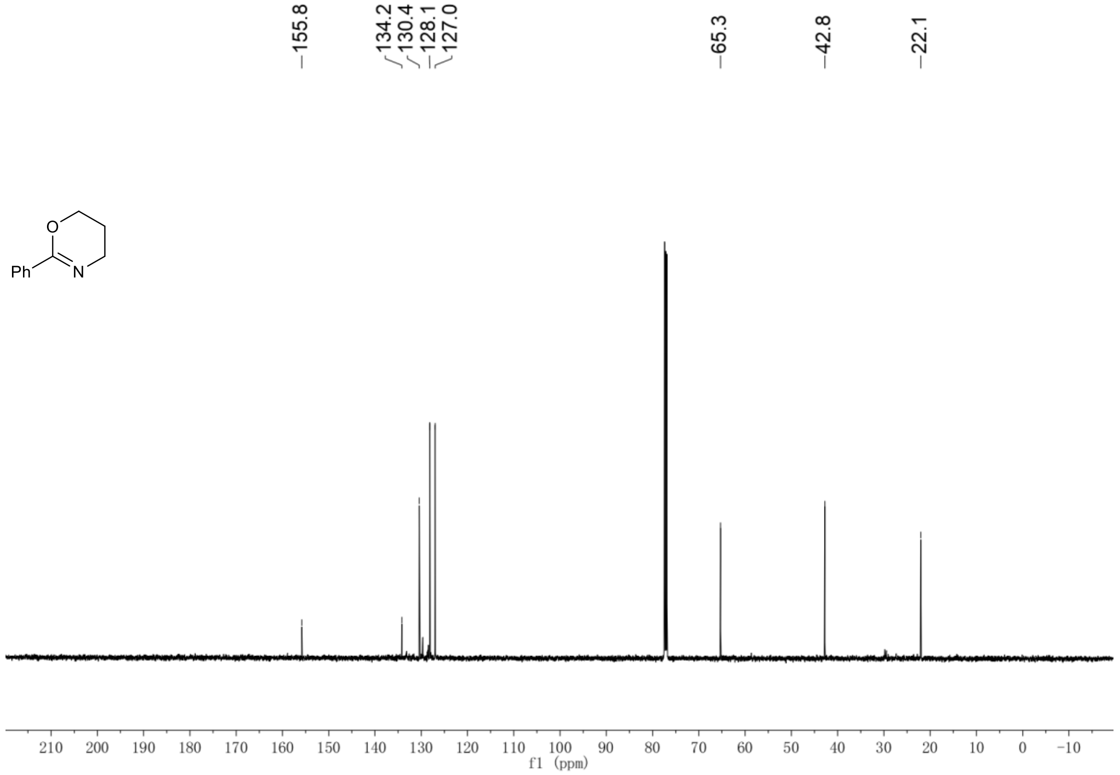

29, ^{1}H NMR (500 MHz, $CDCl_3$)

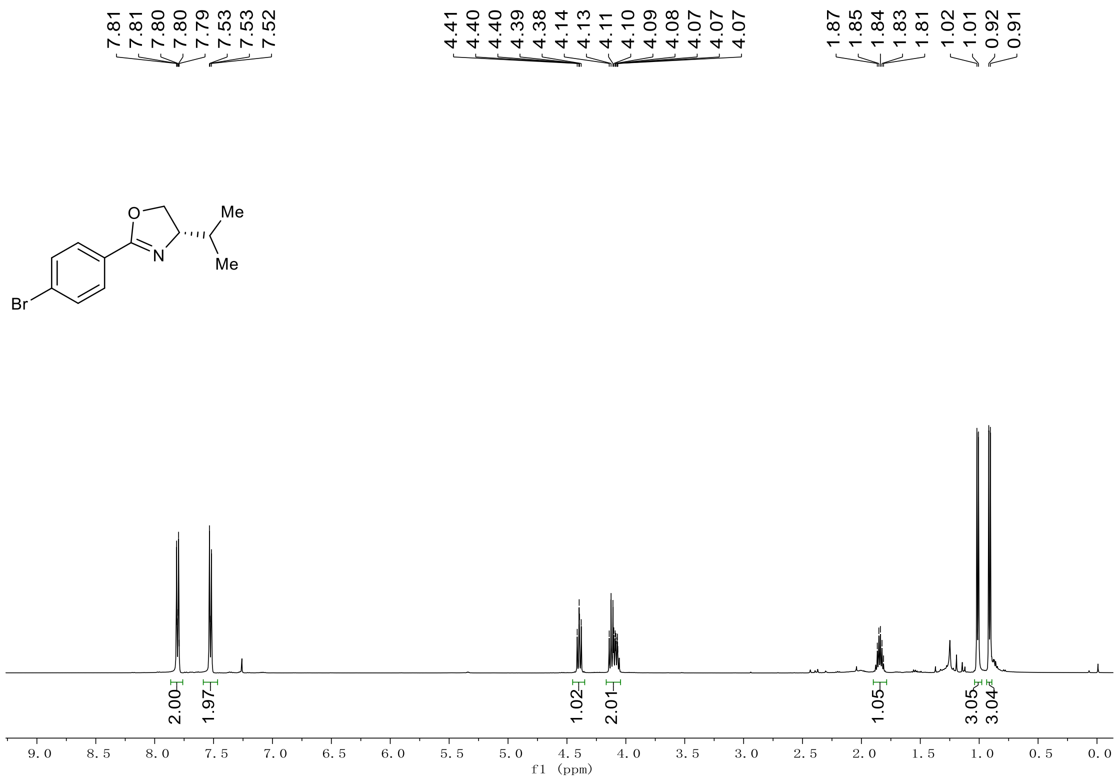

29, ^{13}C NMR (126 MHz, $CDCl_3$)

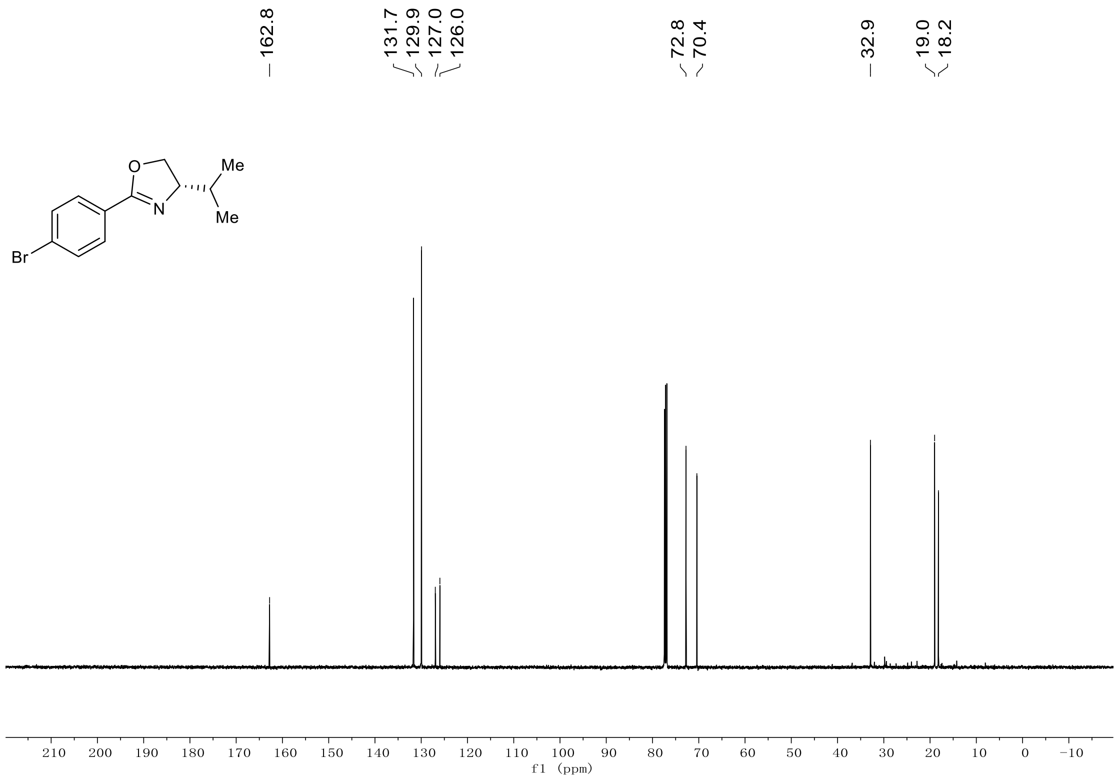

30, ^{1}H NMR (500 MHz, $CDCl_3$)

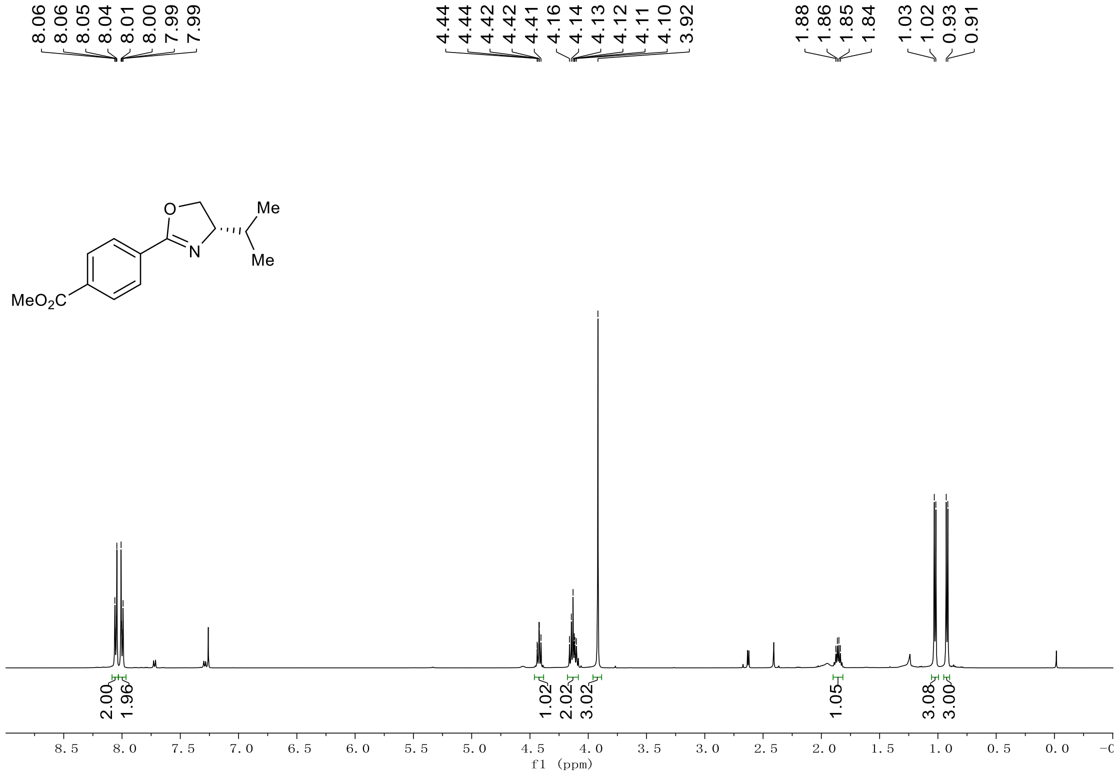

30, ^{13}C NMR (126 MHz, $CDCl_3$)

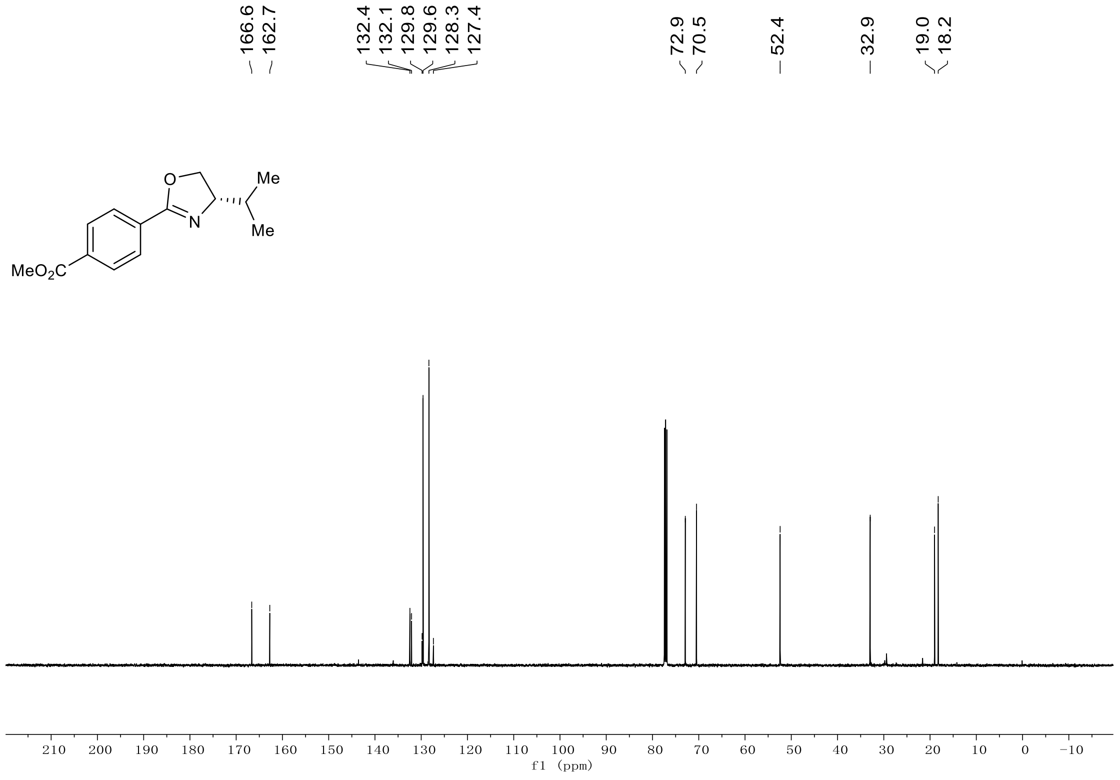

31, ^{1}H NMR (500 MHz, $CDCl_3$)

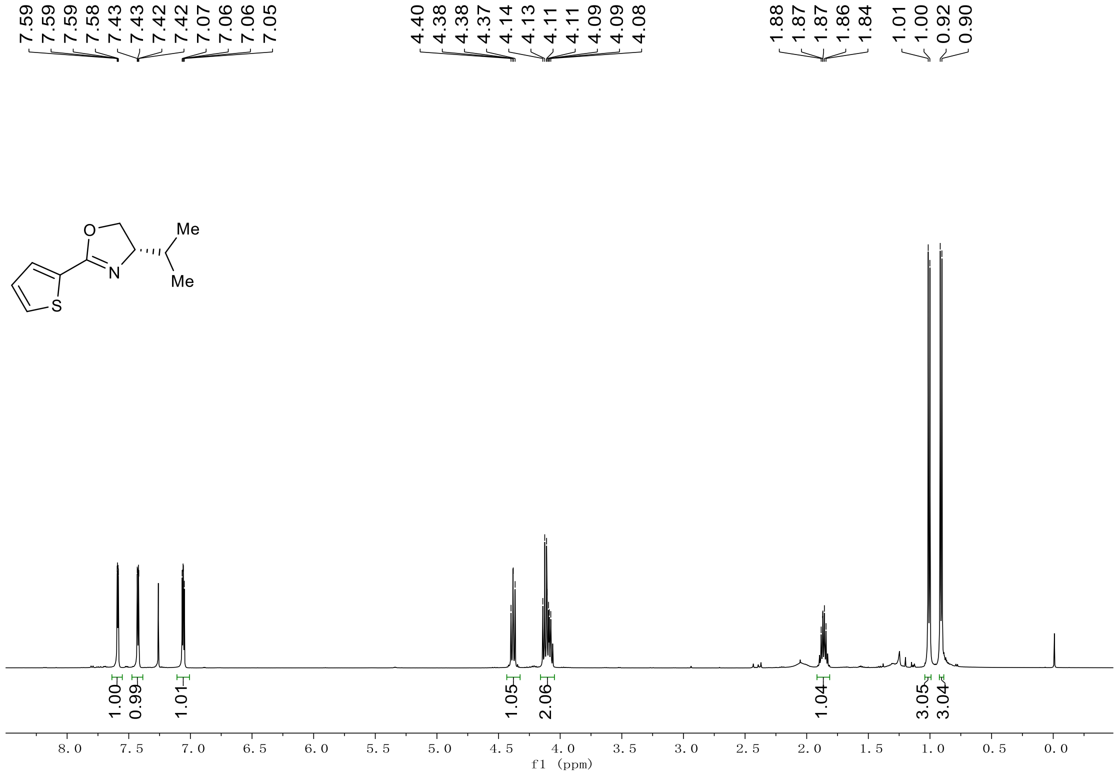

31, ^{13}C NMR (126 MHz, $CDCl_3$)

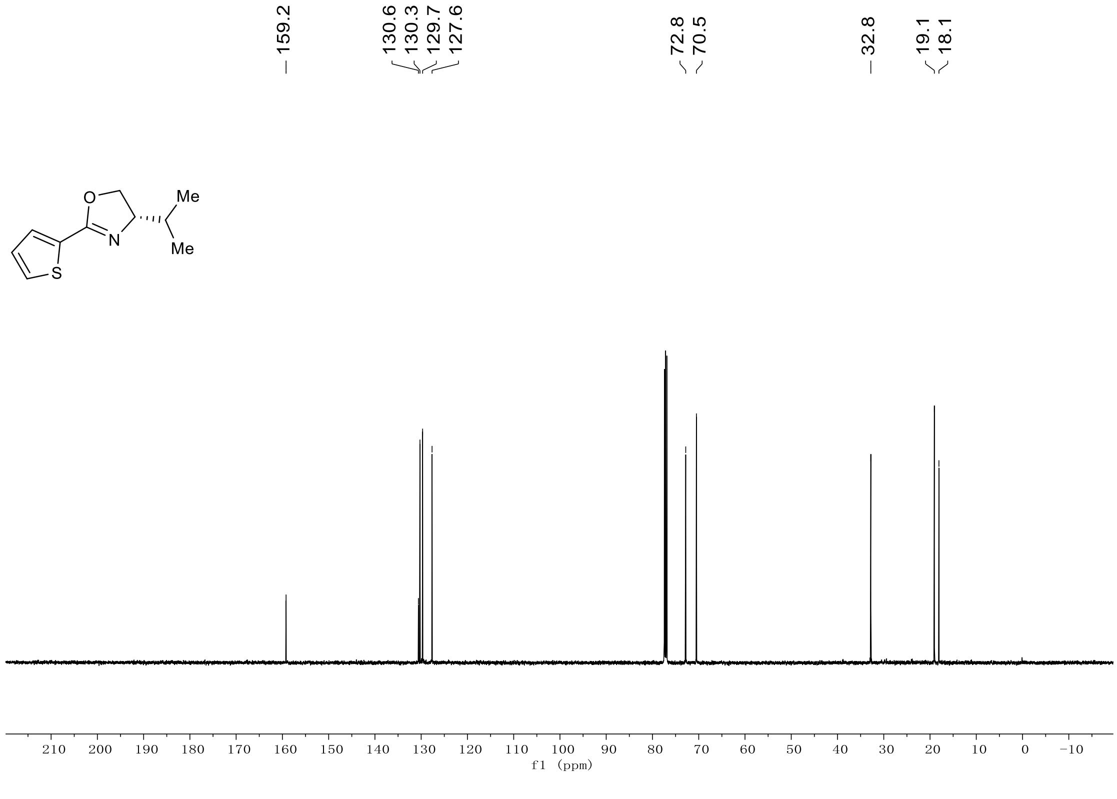

32, ^{1}H NMR (500 MHz, $CDCl_3$)

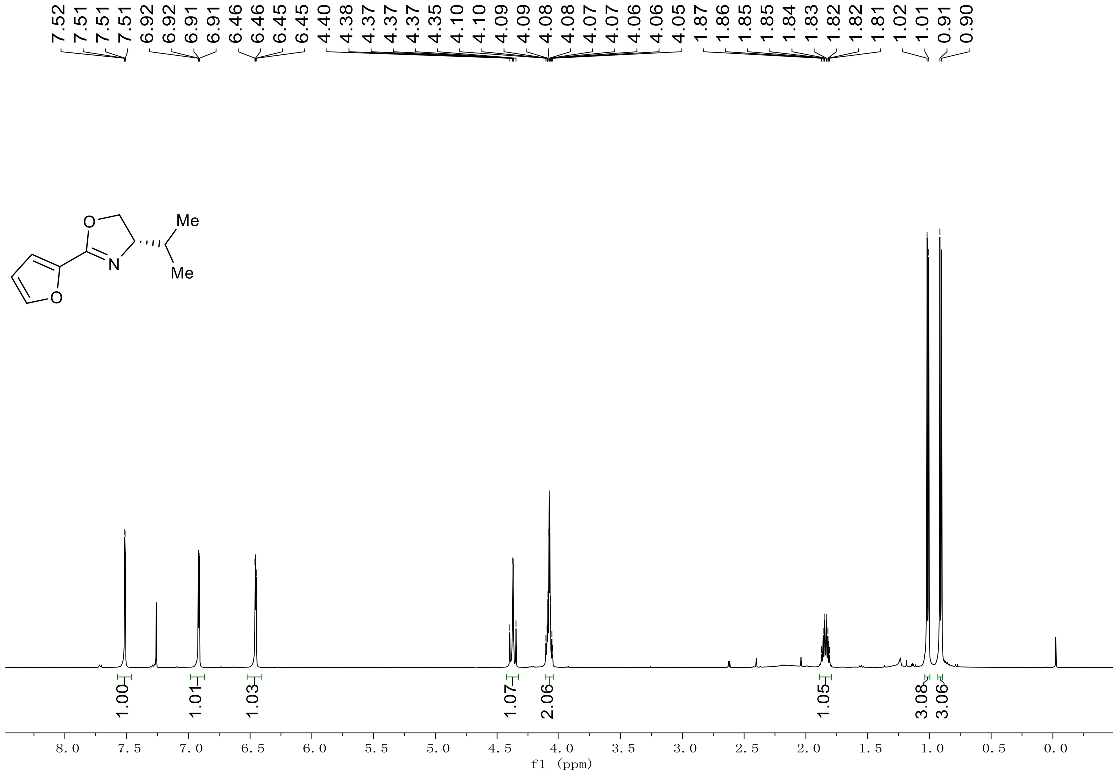

32, ^{13}C NMR (126 MHz, $CDCl_3$)

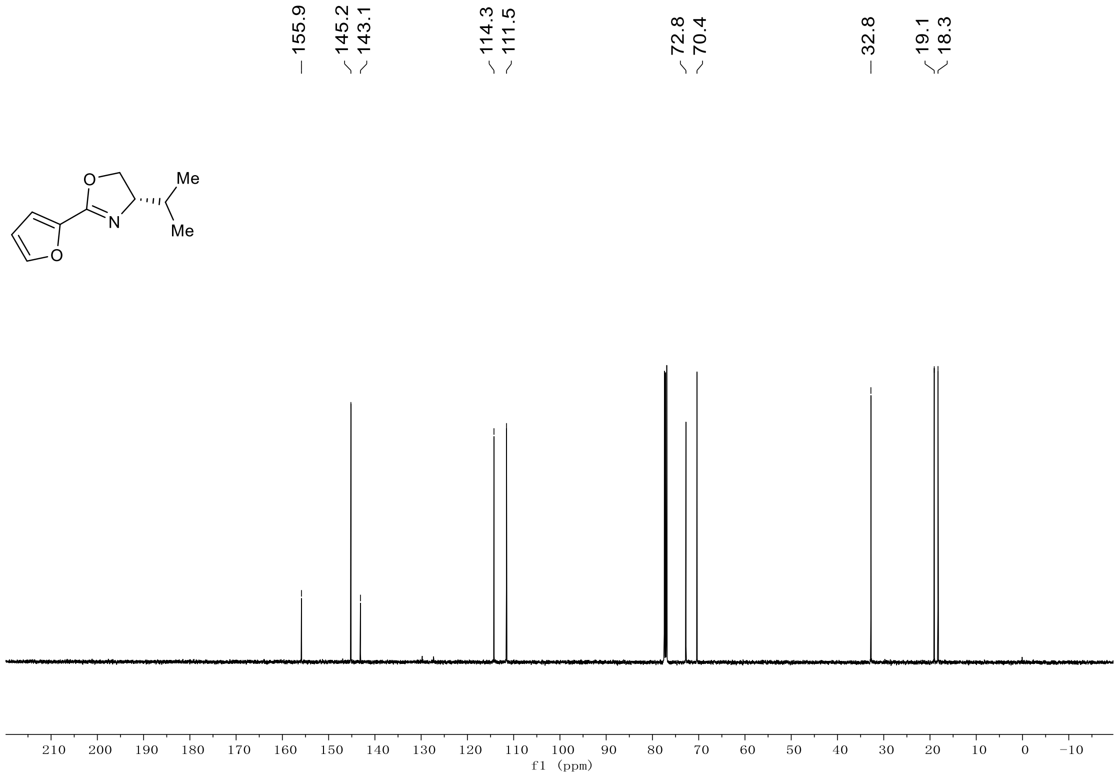

Supplement: Supplementary file 1 [file molecules-27-09042-s001.zip › molecules-2030271-supplementary.pdf]
